# Decoupling Redox Potentials and Excited State Energies in Substituted Chromium(III) Chromophores

**DOI:** 10.1002/chem.202502668

**Published:** 2025-09-27

**Authors:** Steven Sittel, Dimitri Zorn, Alexandra König, Jonas Marcel Grenz, Christoph Förster, Robert Naumann, Katja Heinze

**Affiliations:** ^1^ Department of Chemistry Johannes Gutenberg University Mainz Duesbergweg 10–14 55128 Mainz Germany

**Keywords:** chromium, photophysics, redox chemistry, structure‐property relationships, trifluoromethylation

## Abstract

Redox and excited state properties of classical organic and precious metal charge‐transfer (CT) photocatalysts can be tuned by substituent effects—however, not independently, as redox and chromophore units are strongly entangled. In contrast, substituent effects should selectively address electrochemical properties of photoactive complexes based on metal‐centered spin‐flip (SF) excited states lacking CT character without compromising excited state properties. Yet, detailed structure‐activity relationships for SF chromophores are lacking. We demonstrate that the redox potentials of polypyridine chromium(III) complexes, that feature purely metal‐centered SF states, can be selectively tuned by incremental substituent effects, while the excited state energies and lifetimes remain unaffected. This delivers a unique series of chromophores with identical optical properties, but redox potentials tuned by increments. We believe, that such precisely designed chromophore series provide a unique tool for the systematic investigation of photoredox reactions and recombination processes relevant to photoredox catalytic cycles.

## Graphical abstract

Redox and excited state properties of organic and precious metal CT photocatalysts are strongly entangled. In contrast, redox potentials of polypyridine chromium(III) complexes, that feature purely metal‐centered SF states, are selectively tuned by incremental substituent effects of the CF_3_ group, while excited state energies and lifetimes remain unaffected. Such precisely designed chromophores provide a unique tool for the systematic investigation of elementary reaction steps relevant to photoredox catalytic cycles.

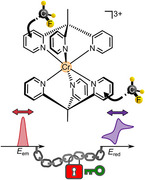

## 1 Introduction

**Scheme 1:**
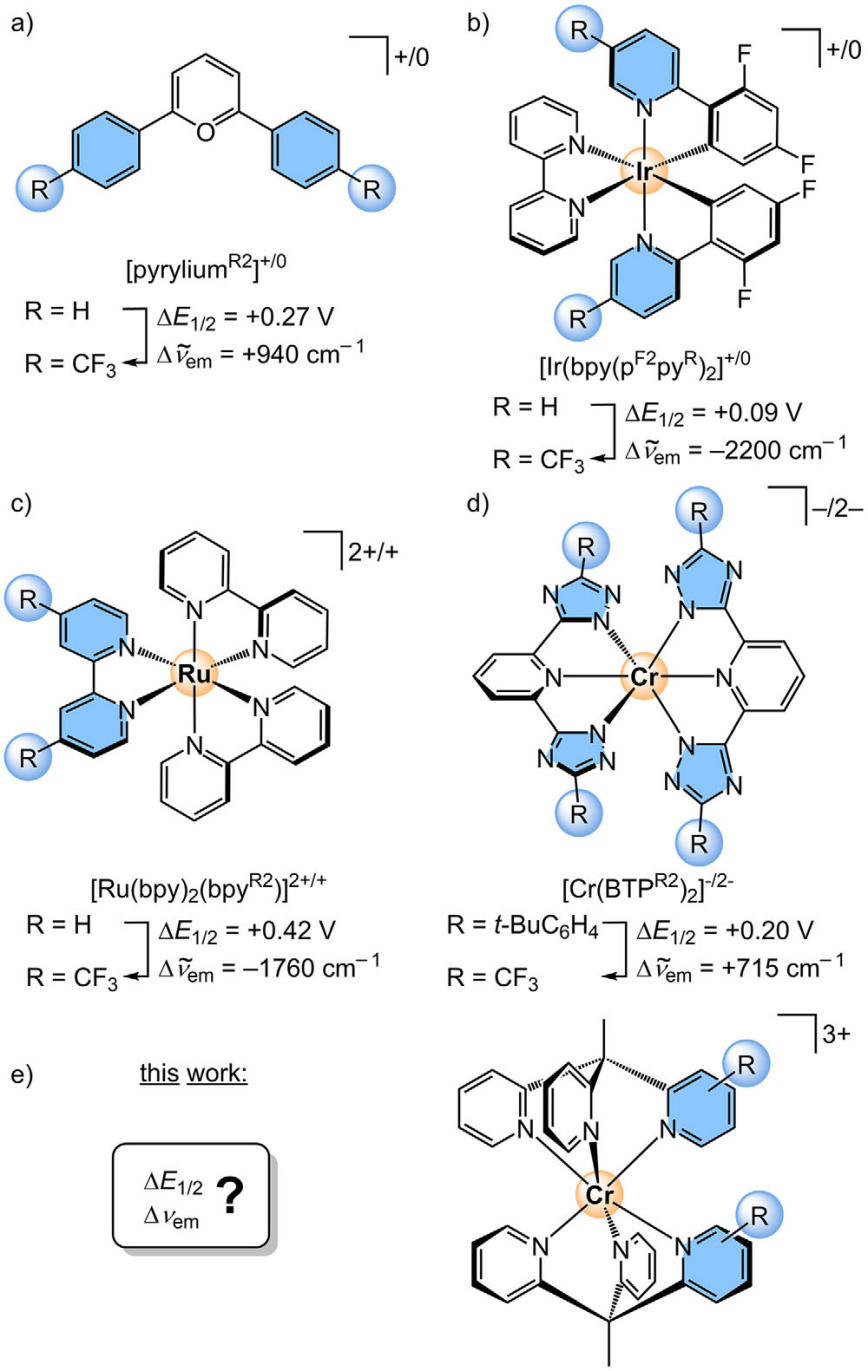
a)–d) Effects of CF_3_ substitution on redox potential *E*_1/2_ and emission energy $\tilde{\nu }$_em_ in typical organic and inorganic chromophores^[^ ^10^, ^11^, ^12^, ^13^, ^14^, ^17^ ^]^ and e) chromium(III) complexes studied in this work.

Long excited state lifetimes, tunable redox potentials and high photo‐ and redox stabilities mark desirable properties of photoactive materials. An electron‐withdrawing or donating substituent^[^ ^1^ ^]^ located close to the redox‐active moiety of a molecule, for example when a coordinated ligand is reduced or oxidized, will affect the redox potentials. Similarly, electron‐withdrawing or ‐donating substituents affect the wavefunction of the chromophoric moiety, so that excited state properties will be modified by substituent effects as well. Due to the entanglement of redox and chromophoric units of typical organic and metal complex charge‐transfer (CT) chromophores, substituents influence both properties concomitantly.^[^ ^2^, ^3^, ^4^, ^5^, ^6^ ^]^ This concept is often simply described by the respective HOMOs and LUMOs and their energies. This seemingly general finding has been amply demonstrated with organic emitters and CT metal complex emitters,^[^ ^2^, ^3^, ^4^, ^5^ ^]^ such as cyanoarenes,^[^ ^7^ ^]^ 9‐mesityl acridinium ions,^[^ ^8^, ^9^ ^]^ pyrylium ions,^[^ ^10^ ^]^ or ruthenium(II), iridum(III), or copper(I) derivatives (see Scheme 1 for selected examples of CF_3_ substitution).^[^ ^11^, ^12^, ^13^, ^14^, ^15^, ^16^, ^17^ ^]^

The group electronegativity of CF_3_ groups (EN_CF3_ = 3.0) is similar to the electronegativity of chlorine (EN_Cl_ = 3.0) on the Pauling scale.^[^ ^18^ ^]^ Hence, trifluoromethyl substituents are expected to substantially affect redox and excited state properties in organic and CT chromophores. For example, substituting pyrylium cations [pyrylium]^+^ with two CF_3_ groups increases the [pyrylium]^+/0^ potential by Δ*E*_1/2_ = +0.27 V and shifts the fluorescence band by $\Delta {\tilde{\nu }}_{\mathrm{em}}$ = +940 cm^−1^ to higher energies (Scheme 1a).^[^ ^10^ ^]^ Installation of CF_3_ substituents at the pyridine donors in 5‐position in [Ir(bpy)(ppy^F2,R^)_2_]^+^ complexes increases the [Ir(bpy)(ppy^F2,R^)_2_]^+/0^ potential by + 0.09 V and lowers the emission energy by −2200 cm^−1^ (Scheme 1b, bpy = 2,2′‐bipyridine; Hppy = 2‐phenylpyridine).^[^ ^14^ ^]^ Strong effects on both redox potentials and excited state energies (absorption or emission) were observed by CH_3_ → CF_3_ substitution on β‐diketiminato ancillary ligands in copper(I) and cyclometalated iridium(III) complexes.^[^ ^15^, ^16^ ^]^ Two CF_3_ substituents in a 4,4′‐substituted bipyridine ligand shift the potential of the [Ru(bpy)_3_]^2+/+^ couple by + 0.42 V to higher values and the emission band maximum by −1760 cm^−1^ (Scheme 1c).^[^ ^13^ ^]^

Tripodal pyridine ligands can impart favorable photophysical properties to metal ions, in particular earth‐abundant metal ions,^[^ ^19^, ^20^, ^21^, ^22^ ^]^ including vanadium(II),^[^ ^23^ ^]^ chromium(III),^[^ ^24^, ^25^, ^26^, ^27^ ^]^ iron(II),^[^ ^28^, ^29^ ^]^ nickel(II)^[^ ^30^ ^]^ and molybdenum(0).^[^ ^31^, ^32^ ^]^ This includes long excited state photoluminescence (PL) lifetimes thanks to increased ligand field strengths^[^ ^23^, ^24^, ^25^, ^26^, ^27^, ^28^, ^29^, ^30^ ^]^ and/or high local symmetry^[^ ^23^, ^24^, ^25^, ^26^, ^27^ ^]^ as well as high (photo)chemical stability due to the *trans* effect.^[^ ^31^, ^32^ ^]^ In particular, the extremely long PL lifetime of the spin‐flip (SF)^[^ ^33^, ^34^, ^35^, ^36^ ^]^ excited state of the chromium(III) complex [Cr(tpe)_2_]^3+^ (tpe = 1,1,1‐tris(pyrid‐2‐yl)ethane) with several milliseconds at 293 K,^[^ ^24^ ^]^ a useful excited redox potential and exceptional photo and redox stability enables challenging photoredox catalytic reactions, including uphill processes, reusing and even recycling of the photocatalyst with over 90 % yield.^[^ ^24^, ^25^, ^26^ ^]^ Yet, the distinct influence of substituents on the redox and excited state properties of such SF chromophores is unknown.^[^ ^35^ ^]^

Substituent effects^[^ ^1^ ^]^ on the PL energy and lifetime of purely metal‐centered SF states without significant ligand contributions, as found in many chromium(III) complexes, are expected to be much smaller than in CT complexes,^[^ ^6^ ^]^ while ligand‐based redox potentials should be significantly affected. However, installation of two CF_3_ units per tridentate ligand in anionic chromium(III) complexes [Cr(BTP^R2^)_2_]^−^− both shifts the redox potential (by + 0.20 V) and the emission energy (by + 715 cm^−1^), likely because of ligand admixture to the SF excited states (Scheme 1d).^[^ ^17^ ^]^ Hence, the predicted decoupling of redox and excited state properties in pure SF emitters has not been experimentally confirmed.^[^ ^6^, ^33^ ^]^ Yet, such a series could be very useful for systematically studying photoinduced electron transfer and competing charge recombination processes^[^ ^37^ ^]^ (cage escape)^[^ ^38^ ^]^ with tuned ground and excited state redox potentials, but identical excited state energies.

In the present study, we leverage the redox potential of tripodal polypyridine chromium(III) chromophores with very pure metal‐centered SF states by installing electron‐withdrawing trifluoromethyl substituents^[^ ^1^, ^18^ ^]^ at the ligand without compromising the favorable photophysical properties (Scheme 1e). First, we describe the synthesis of a CF_3_‐substituted tripodal pyridine ligand tpe^CF3^ and delineate a synthetic pathway to coordinate such a very electron‐deficient pyridine ligand to already electron‐poor chromium(III) ions to give the CF_3_‐substituted complexes with one or two trifluoromethyl substituents. Then we discuss the PL and redox properties as well as the stabilities of the reduced CF_3_‐substituted chromium(III) complexes. Finally, quantum chemical calculations (density functional theory, DFT; complete‐active‐space self‐consistent field‐N‐electron valence perturbation theory to second order, CASSCF‐NEVPT2) underpin the experimental findings, which will allow future *in silico* design of redox‐active SF chromophores.

## 2 Results and Discussion

### 2.1 Synthesis and Purification

**Scheme 2:**
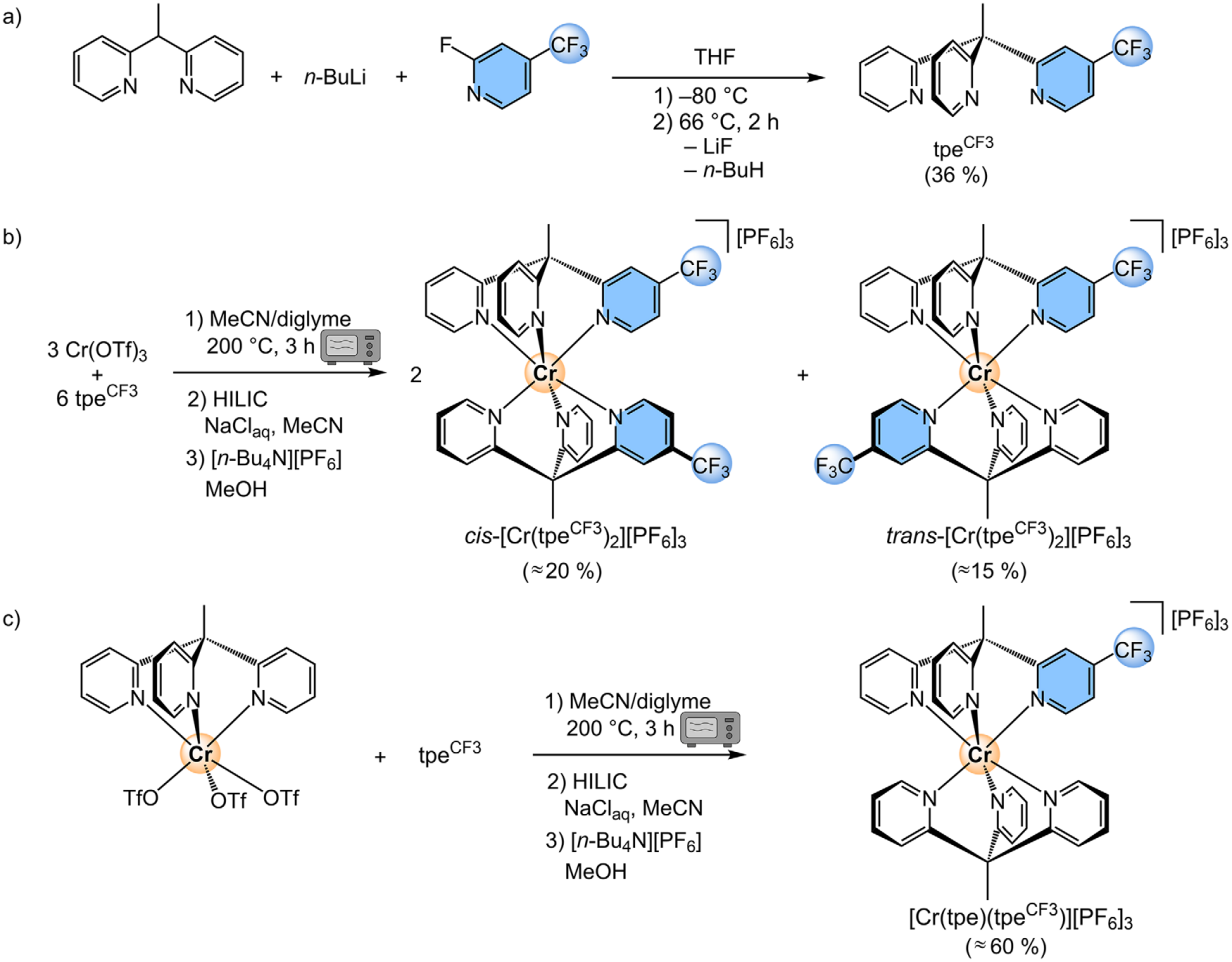
Synthesis of a) the ligand tpe^CF3^, b) the homoleptic *cis*‐ and *trans*‐complex isomers *cis*‐[Cr(tpe^CF3^)_2_][PF_6_]_3_ and *trans*‐[Cr(tpe^CF3^)_2_][PF_6_]_3_ and c) the heteroleptic monosubstituted complex [Cr(tpe)(tpe^CF3^)][PF_6_]_3_.

The novel trifluoromethylated tripodal ligand tpe^CF3^ was obtained in a two‐step procedure from commercially available starting materials. 1,1‐Bis(2‐pyridyl)ethane, prepared from 2‐ethylpyridine and 2‐fluoropyridine according to a literature procedure,^[^ ^39^, ^40^ ^]^ was treated with *n*‐butyl lithium and subsequently with 2‐fluoro‐4‐(trifluoromethyl)pyridine to give crystalline tpe^CF3^ in 38 % isolated yield (Scheme 2a). This new ligand was fully characterized by heteronuclear NMR and IR spectroscopy and mass spectrometry (Figures S1–S8). The bands for the symmetric and asymmetric C–F stretching vibrations of the CF_3_ substituent are found at 1329 and 1131 cm^−1^, respectively.^[^ ^41^ ^]^

Due to the electron‐poor nature of the CF_3_‐substituted ligand, classical syntheses using substitutionally labile chromium(II) salts^[^ ^25^, ^42^ ^]^ or via in situ reduction of chromium(III) to chromium(II) with zinc^[^ ^43^ ^]^ that worked well for the unsubstituted ligand tpe,^[^ ^25^ ^]^ are not feasible, as chromium(II) ions reduce the tpe^CF3^ ligand to its radical anion thus preventing complex formation. To coordinate the very electron‐poor CF_3_‐substituted ligand directly to electron‐deficient chromium(III) ions, Cr(OTf)_3_^[^ ^44^ ^]^ with labile triflato ligands^[^ ^45^, ^46^ ^]^ was treated with two equivalents of tpe^CF3^ in a microwave‐assisted reaction at 200 °C to give the homoleptic complexes *cis*‐[Cr(tpe^CF3^)_2_]^3+^ and *trans*‐[Cr(tpe^CF3^)_2_]^3+^ (Scheme 2b). HPLC analysis confirmed the presence of a statistical 2:1 mixture of the two isomers (Figure S9). Hydrophilic interaction liquid chromatography (HILIC, Figure S10) succeeded in separating the isomers. Salt metathesis with [*n*‐Bu_4_N][PF_6_] finally delivered the *cis*‐ and *trans*‐isomers with two CF_3_ substituents as yellow, crystalline PF_6_ salts (Scheme 2b). Assignment of the *cis*/*trans* configuration to the two isomeric complexes is first based on the initial statistical *cis*:*trans* ratio of 2:1 obtained in the synthesis. As further confirmation, an HPLC experiment using a chiral column and detection via circular dichroism spectroscopy was conducted. While the achiral *trans*‐isomer is unaffected by the chiral column, the chiral racemic *cis*‐isomers split into the enantiomers in a 1:1 ratio on the chiral column (Figure S11) allowing a definitive assignment of the configuration.

The heteroleptic complex [Cr(tpe)(tpe^CF3^)]^3+^ required a sequential synthetic approach^[^ ^47^, ^48^, ^49^ ^]^ via the intermediate tpe triflato complex Cr(OTf)_3_(tpe) (Scheme 2c). Cr(OTf)_3_(tpe) was obtained from CrCl_3 _× 6H_2_O and tpe^[^ ^25^ ^]^ followed by halide abstraction with trimethylsilyl trifluoromethanesulfonate as purple crystalline material, suitable for single crystal X‐ray analysis (Figure S12, Table S1, CCDC 2 479 151). All triflates are coordinated to the chromium(III) center with Cr–O distances below 2 Å (Table S1), similar to other polypyridine triflato chromium(III) complexes, such as *mer*‐Cr(OTf)_3_(tpy) and *mer*‐Cr(ddpd)(OTf)_3_ (tpy = 2,2“:6”,2“‐terpyridine, ddpd = *N*,*N*”‐dimethyl‐*N*,*N*'‐dipyridine‐2‐yl‐pyridine‐2,6‐diamine),^[^ ^47^, ^48^, ^49^ ^]^ yet in a facial fashion due to the tripodal tpe ligand. Again, coordination of tpe^CF3^ to chromium(III) in Cr(OTf)_3_(tpe) substituting the triflato ligands required microwave activation at 200 °C. These harsh conditions, however, lead to partial ligand scrambling, so that the resulting material consists of the desired heteroleptic [Cr(tpe)(tpe^CF3^)]^3+^ complex (major product, 67 %) in addition to the homoleptic complexes [Cr(tpe)_2_]^3+^ and (likely) [Cr(tpe^CF3^)_2_]^3+^ (minor products) according to HPLC analysis (Figure S13). To separate the heteroleptic complex from the homoleptic ones, the mixture was subjected to HILIC (Figure S14). Yellow, crystalline [Cr(tpe)(tpe^CF3^)][PF_6_]_3_ was finally obtained after salt metathesis with [*n*‐Bu_4_N][PF_6_] and recrystallization (Scheme 2c).

In all complex syntheses, the yields of the raw products before HILIC are much higher than for the purified ones. The purity of all CF_3_‐substituted complexes was confirmed by analytical HPLC analysis (Figures S10, S14). All CF_3_‐substituted complexes were furthermore characterized by ESI^+^ mass spectrometry in MeCN (Figures S15–S17) showing peaks for the respective trication and corresponding ion clusters with one and two counter ions, respectively. No peaks for dissociated tpe^CF3^ were observed, suggesting appreciable complex stability in spite of the electron deficiency of the tpe^CF3^ ligand. IR bands for the symmetric and asymmetric C–F stretching vibrations of the CF_3_ groups^[^ ^41^ ^]^ of the complexes [Cr(tpe)(tpe^CF3^)][PF_6_]_3_, *cis*‐[Cr(tpe^CF3^)_2_][PF_6_]_3_ and *trans*‐[Cr(tpe^CF3^)_2_][PF_6_]_3_ are found at 1335/1159 cm^−1^, 1334/1144 cm^−1^ and 1337/1149 cm^−1^, respectively, in addition to the intense characteristic P–F vibrational bands of the counter ions (Figures S18–S20).

### 2.2 Photophysical Properties

The UV/vis/near‐infrared (NIR) absorption spectra of all CF_3_‐substituted complexes very much resemble that of the unsubstituted complex [Cr(tpe)_2_]^3+ [^ ^24^ ^]^ with the low‐energy spin‐allowed ligand field transitions (^4^A_2_ → ^4^T_2_) appearing at *λ*_abs_ = 431, 433, 432 and 432 nm ($\tilde{\nu }$_abs_ = 23 200, 23 100, 23 150, 23 150 cm^−1^) for [Cr(tpe)_2_]^3+^,^[^ ^24^ ^]^ [Cr(tpe)(tpe^CF3^)]^3+^, *cis*‐[Cr(tpe^CF3^)_2_]^3+^ and *trans*‐[Cr(tpe^CF3^)_2_]^3+^, respectively (Figures S21–S23). The molar absorption coefficients of all complexes are with *ε* = 35 M^−1^ cm^−1^ identical as well. This finding suggests that the local center of symmetry of the Cr(py)_6_ coordination is not disturbed in the monosubstituted complex [Cr(tpe)(tpe^CF3^)]^3+^ and in the *cis*‐complex *cis*‐[Cr(tpe^CF3^)_2_]^3+^, so that Laporte's rule^[^ ^50^ ^]^ applies strongly for all complexes. Consequently, the metal‐centered quartet states are unaffected by the CF_3_ substituents in the Franck‐Condon geometry with respect to energy and symmetry‐breaking.

**Figure 1:**
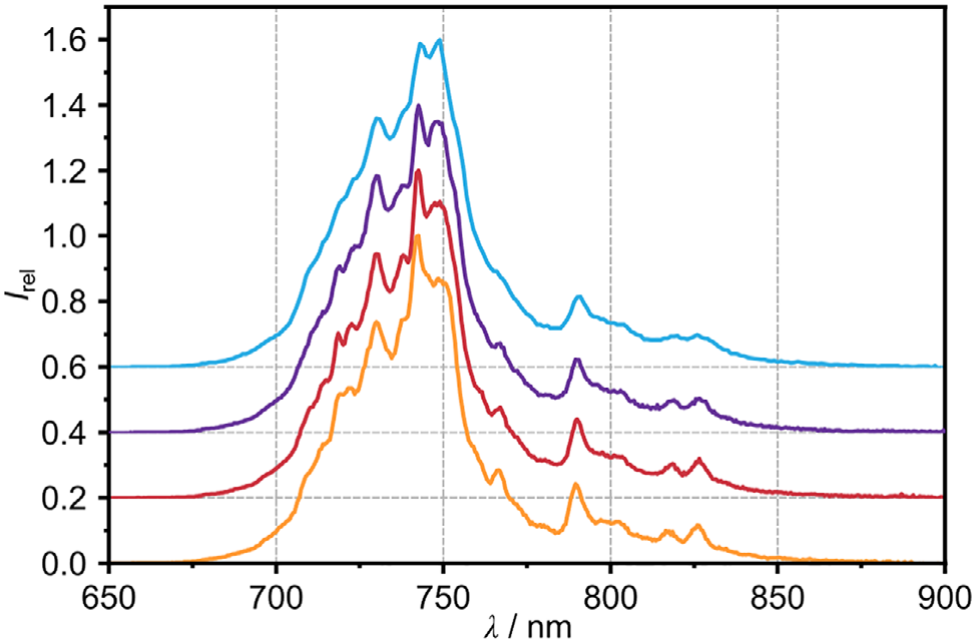
Stacked normalized PL spectra of [Cr(tpe)_2_][PF_6_]_3_ (orange),^[^ ^24^, ^25^, ^27^ ^]^ [Cr(tpe)(tpe^CF3^)][PF_6_]_3_ (red, offset by 0.2), *cis*‐[Cr(tpe^CF3^)_2_][PF_6_]_3_ (purple, offset by 0.4) and *trans*‐[Cr(tpe^CF3^)_2_][PF_6_]_3_ (blue, offset by 0.6) in MeCN at 293 K.

All complexes emit in the NIR spectral region in solution at 293 K from their lowest‐energy doublet states (^2^E/^2^T_1_) with the highest peak maxima at *λ*_em_ = 748, 743, 743, and 748 nm ($\tilde{\nu }$_em_ = 13 370, 13 460, 13 460, and 13 370 cm^−1^) for [Cr(tpe)_2_]^3+^,^[^ ^24^, ^25^, ^27^ ^]^ [Cr(tpe)(tpe^CF3^)]^3+^, *cis*‐[Cr(tpe^CF3^)_2_]^3+^ and *trans*‐[Cr(tpe^CF3^)_2_]^3+^, respectively (Figures 1 and S21–S23). The excitation spectra closely follow the absorption spectra, confirming that the PL arises from the complexes (Figures S21–S23). The largest energy difference of the PL maxima amounts to Δ$\tilde{\nu }$_em_ = 90 cm^−1^ close to the spectral resolution. This confirms our expectation that the energy of the emissive doublet states in these polypyridine chromium(III) complexes is essentially independent from the number (one/two) and relative position (*cis*/*trans*) of the trifluoromethyl substituents at the tripodal ligands. Furthermore, the characteristic emission band pattern of the centrosymmetric complex [Cr(tpe)_2_]^3+^ (representing progressions of ungerade Cr–N enabling modes)^[^ ^24^ ^]^ is fully mirrored by the CF_3_‐substituted complexes substantiating that symmetry‐breaking is absent in these complexes (Figure 1). This electronic symmetry‐conservation of the electronic states in spite of the symmetry‐breaking imposed by the nuclei had been already inferred from the quartet absorption bands (Figures S21–S23).

The PL quantum yields (*Φ* = 8.2 (different solvent: deaerated D_2_O/DClO_4_^[^ ^24^ ^]^), 4.9, 6.0 and 6.2 %) and PL lifetimes (*τ* = 3.5, 2.6, 3.0 and 3.3 ms) are very high and very similar^[^ ^51^, ^52^ ^]^ as well for the complexes [Cr(tpe)_2_]^3+^,^[^ ^24^, ^25^, ^27^ ^]^ [Cr(tpe)(tpe^CF3^)]^3+^, *cis*‐[Cr(tpe^CF3^)_2_]^3+^, and *trans*‐[Cr(tpe^CF3^)_2_]^3+^, respectively (Figures S21–S23). The small differences in *Φ* and *τ* are very likely due to the presence of traces of quenching molecules.^[^ ^51^, ^52^ ^]^ The resulting radiative and nonradiative rate constants are very small and similar as well (*k*_r_ = 19 – 23 s^−1^ and *k*_nr_ = 260 – 370 s^−1^).

In air‐saturated MeCN solution, the excited state lifetimes are reduced from the millisecond range (2.6 – 3.5 ms) to *τ*_O2_ = 320,^[^ ^24^ ^]^ 333, 360, and 360 µs (Figures S21–S23) suggesting quenching of the emission by triplet oxygen as often observed for chromium(III) complexes with long‐lived excited doublet states.^[^ ^17^, ^24^, ^25^, ^36^, ^44^, ^53^, ^54^, ^55^ ^]^ The quenching efficiency is very similar for all complexes as well.

In contrast to bulky mesityl or 2,4,6‐triisopropylphenyl substituents attached to molecular rubies that protect the chromium(III) center from oxygen quenching,^[^ ^36^ ^]^ the CF_3_ substituents in 4‐positions of the pyridines in the present complexes exert no shielding effect.

In summary, the excited state properties ($\tilde{\nu }$_abs_, *ε*, $\tilde{\nu }$_em_, *τ*, *Φ*, *k*_r_, *k*_nr_, *τ*_O2_) of the metal‐centered excited quartet and doublet states of the chromium(III) chromophores are essentially unaffected by the presence, number, and position of CF_3_ substituents. This confirms our expectation that purely metal‐centered excited states are only weakly affected by substituent effects.

### 2.3 Redox Properties

**Figure 2:**
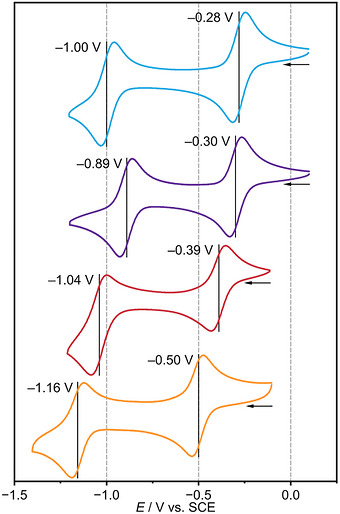
Cyclic voltammograms of [Cr(tpe)_2_][PF_6_]_3_ (orange), [Cr(tpe)(tpe^CF3^)][PF_6_]_3_ (red), *cis*‐[Cr(tpe^CF3^)_2_][PF_6_]_3_ (purple) and *trans*‐[Cr(tpe^CF3^)_2_][PF_6_]_3_ (blue) 1 mM in a 100 mM solution of [*n*‐Bu_4_N][PF_6_] in MeCN.

All tricationic complexes are reduced in two reversible one‐electron reduction steps [CrL_2_]^3+/2+^ and [CrL_2_]^2+/+^ (Figure 2 and S24–S26). The [CrL_2_]^3+/2+^ and [CrL_2_]^2+/+^ reduction potentials (vs. SCE) amount to *E*_1/2_([CrL_2_]^3+/2+^) = −0.50, −0.39, −0.30, −0.28 V, and *E*_1/2_([CrL_2_]^2+/+^) = −1.16, −1.04, −0.89, −1.00 V for [Cr(tpe)_2_]^3+^,^[^ ^24^, ^25^, ^27^ ^]^ [Cr(tpe)(tpe^CF3^)]^3+^, *cis*‐[Cr(tpe^CF3^)_2_]^3+^ and *trans*‐[Cr(tpe^CF3^)_2_]^3+^, respectively.

Clearly, the number of CF_3_ substituents significantly affects the [CrL_2_]^3+/2+^ and [CrL_2_]^2+/+^ processes. The reduction potentials are indeed incrementally shifted to higher values by ca. +0.1 V per CF_3_ substituent with the exception of the *trans*‐[Cr(tpe^CF3^)_2_]^2+/+^ couple. This confirms the expectation that substituent effects are additive,^[^ ^56^, ^57^ ^]^ even for processes that are mixed metal/ligand‐centered due to the redox noninnocence of pyridine ligands^[^ ^24^, ^58^, ^59^, ^60^, ^61^ ^]^ (see section Quantum Chemical Assessment). Only the *trans*‐[Cr(tpe^CF3^)_2_]^2+/+^ couple deviates from the incremental correlation, likely because the *trans*‐coordinated CF_3_‐substituted pyridines are strongly conjugated (see section Quantum Chemical Assessment).

The [CrL_2_]^3+/2+^ redox processes were additionally probed by chemical reduction of the tricationic complexes in MeCN using cobaltocene CcH as a reductant. CcH selectively addresses the first reduction process of the complexes, avoiding over‐reduction (*E*_1/2_(CcH^+/0^) = −0.93 V ^[^ ^62^, ^63^ ^]^). Upon addition of CcH, the pale‐yellow MeCN solutions of the tricationic complexes immediately turn deep green.

**Figure 3:**
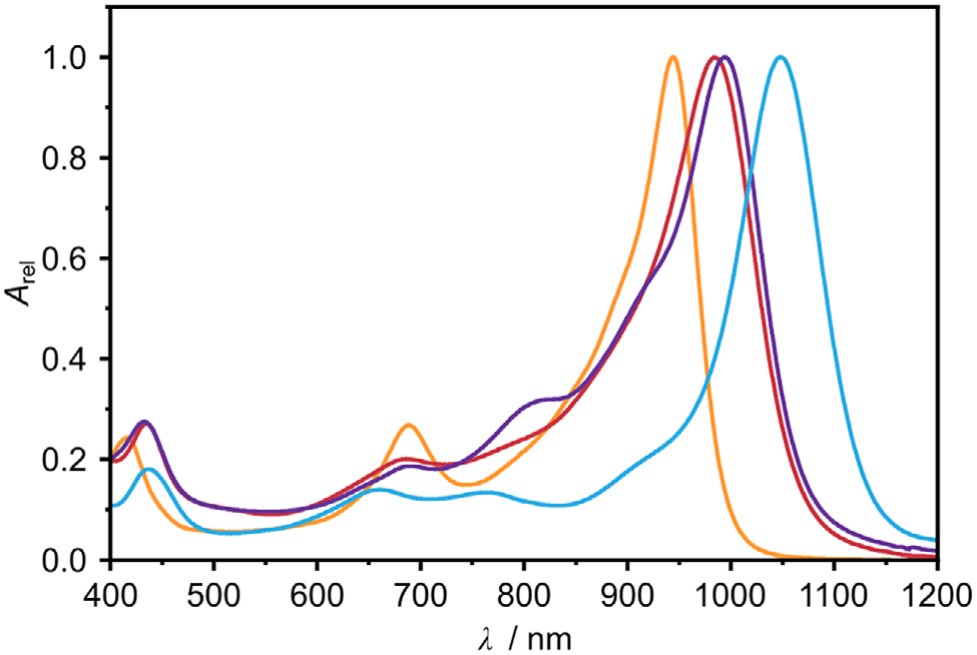
Normalized vis/NIR absorption spectra of [Cr(tpe)_2_]^2+^ (orange), [Cr(tpe)(tpe^CF3^)]^2+^ (red), *cis*‐[Cr(tpe^CF3^)_2_]^2+^ (purple), and *trans*‐[Cr(tpe^CF3^)_2_]^2+^ (blue) in MeCN obtained by quantitative reduction of the respective tricationic complexes with cobaltocene. The resulting cobaltocenium cation does not absorb significantly in this spectral region,^[^ ^64^ ^]^ so that the spectra correspond to the dicationic chromium complexes.

The characteristic color arises from intense bands spanning the entire visible spectral region up to 1100 nm in the NIR (Figures 3 and S27–S30). The most prominent NIR bands of the dicationic complexes peak at *λ*_abs_ = 944, 983, 994, and 1048 nm ($\tilde{\nu }$_abs_ = 10 590, 10 170, 10 060, 9540 cm^−1^, Figures S27–S30). Clearly, the number and position of CF_3_ substituents affect the energies of these absorption bands. This finding suggests that CF_3_‐substituted pyridines significantly contribute to the involved wavefunctions of the NIR transitions of the dicationic complexes. This contrasts the purely metal‐centered excited states of the tricationic complexes (see above) and will be further elaborated in the quantum chemistry section.

Treatment of the dark‐colored solutions of the dications [CrL_2_]^2+^ with ferrocenium hexafluorophosphate [FcH][PF_6_] immediately leads to discoloration, quantitatively regenerating the tricationic complexes [CrL_2_]^3+^ (Figures S31–S34). Consequently, the [CrL_2_]^3+/2+^ redox process is fully reversible not only on the electrochemical, but even on the much longer chemical time scale. This stability confirms that the redox process is not entirely metal‐centred, as chromium(II) would be very labile, but that the additional electron with respect to [CrL_2_]^3+^ is also delocalized on the pyridine ligands—likely preferring the electron‐deficient CF_3_‐substituent ones. Oxidation of the dications with oxygen from air is also possible, but the redox process is very slow due to the unfavorable driving force (Figures S35–S38). In particular, the CF_3_‐substituted complexes [CrL_2_]^2+^ are almost inert toward air (Figures S37–S38).

### 2.4 Quantum Chemical Assessment

All complex trications (quartet ground state; lowest doublet state) and dications (triplet ground state; quintet state) were optimized by DFT methods on the B3LYP/TZVPP level of theory (Figures S39–S42 and Tables S2–S5). Cr─N(pyCF3) bond lengths are only slightly elongated with respect to Cr─N(py) bond lengths, by ca. 0.01, 0.01, and 0.005 Å in the [Cr(tpe)(tpe^CF3^)]^3+^, *cis*‐[Cr(tpe^CF3^)_2_]^3+^ and *trans*‐[Cr(tpe^CF3^)_2_]^3+^ complexes, respectively (Tables S2–S5).

**Figure 4:**
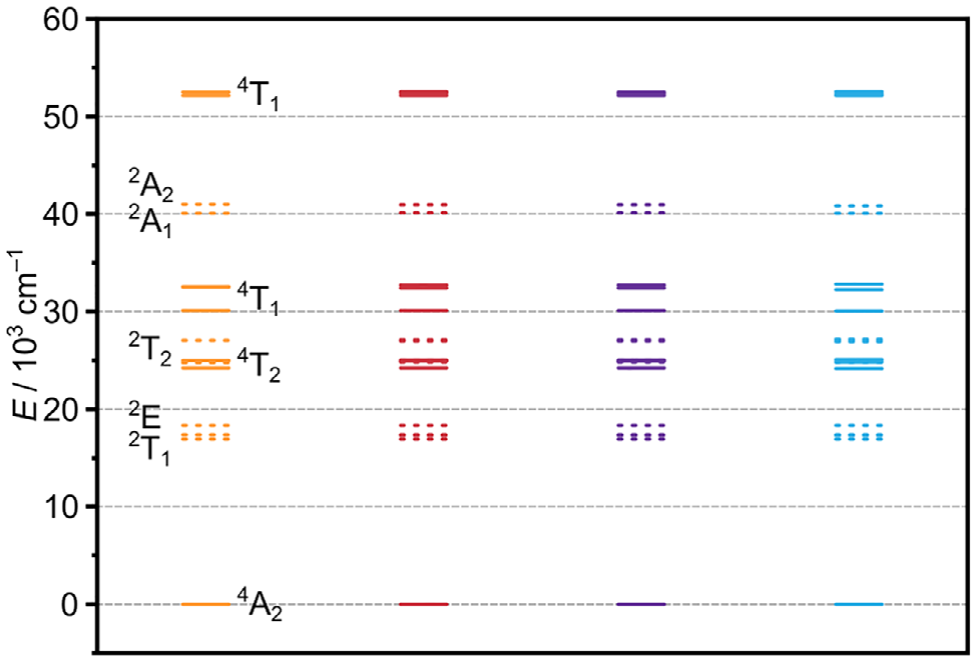
CASSCF(7,12)‐SC/NEVPT2 calculated quartet (solid lines) and doublet (dashed lines) energies (doublet states scaled by 0.89^[^ ^44^, ^49^ ^]^) of [Cr(tpe)_2_]^3+^ (orange), [Cr(tpe)(tpe^CF3^)]^3+^ (red), *cis*‐[Cr(tpe^CF3^)_2_]^3+^ (purple), and *trans*‐[Cr(tpe^CF3^)_2_]^3+^ (blue). Term symbols are given for [Cr(tpe)_2_]^3+^ (*O* point group notation).

As ligand field states, in particular SF states of chromium(III) complexes, are often poorly described by DFT methods, we resorted to multireference CASSCF‐NEVPT2 calculations calculated at the DFT‐optimized ground state geometry for these properties. The active space comprised the dominant bonding/antibonding orbitals formed between chromium and the ligand, namely the five 3d orbitals, two occupied Cr─N σ bonding orbitals, and five 4d orbitals giving an active space of (7,12) (Tables S6–S9).^[^ ^35^, ^44^, ^49^ ^]^ The energies of the thus calculated metal‐centered quartet and doublet states are displayed in Figure 4 (Tables S10–S11).

The calculated energies of the quartet and doublet excited states of all complexes are essentially identical (Figure 4 and Tables S10–S11), confirming the experimentally determined constant ^4^A_2_–^4^T_2_ and ^4^A_2_–^2^E/^2^T_1_ energy differences throughout the series. The high complex symmetry for all complexes is reflected in the weak splitting of the ^4^T_1_ and ^4^T_2_ states (Figure 4 and Tables S10–S11). The ligand field splitting in the ground state geometry is very large, separating the emissive doublet states (^2^E/^2^T_1_) from the lowest‐energy quartet state ^4^T_2_(1). The calculated energies of the lowest‐energy doublet states match the experimental ones reasonably well. The agreement increases further, when a scaling factor of 0.89, that has been determined for a series of pyridine chromium(III) complexes on this level of theory, is applied (15 225 cm^−1^ → 13 550 cm^−1^; 656 nm → 738 nm).^[^ ^44^, ^49^ ^]^ This good agreement confirms that CT states (which are not considered in the active space) do not significantly mix with the doublet states.^[^ ^17^, ^23^, ^65^, ^66^, ^67^, ^68^, ^69^, ^70^ ^]^ Hence, the calculations underpin our assumption that the metal‐centered states, including the SF states, in these chromium(III) complexes are unaffected by substituent effects and symmetry‐breaking.

**Figure 5:**
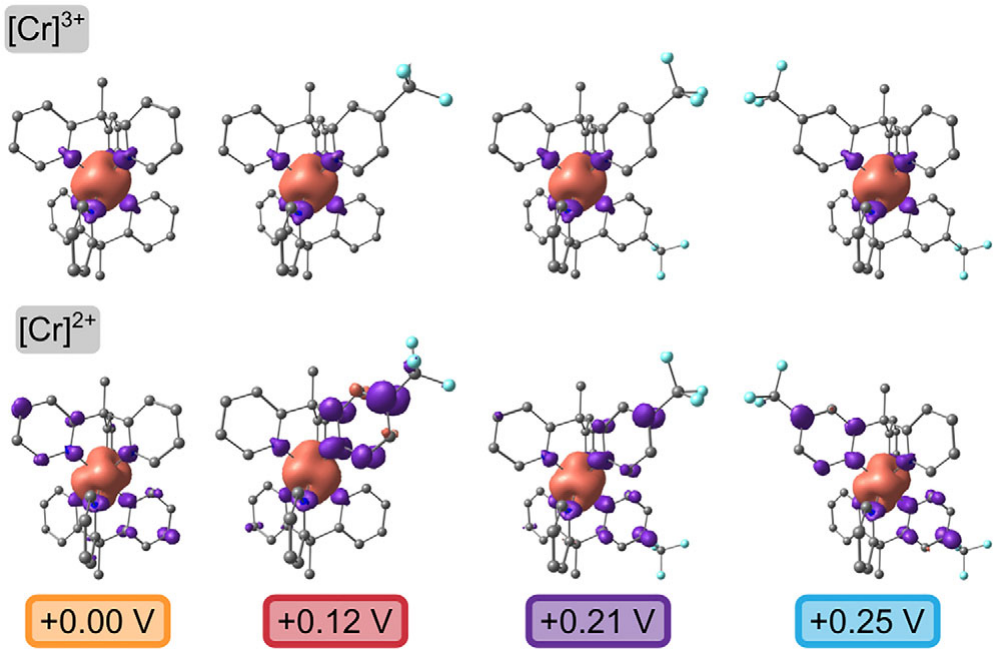
DFT‐optimized geometries and spin‐densities of the [Cr(tpe)_2_]^3+/2+^, [Cr(tpe)(tpe^CF3^)]^3+/2+^, *cis*‐[Cr(tpe^CF3^)_2_]^3+/2+^ and *trans*‐[Cr(tpe^CF3^)_2_]^3+/2+^ complexes and DFT‐calculated redox potentials given relative to the [Cr(tpe)_2_]^3+/2+^ couple set to *E*_½_ = 0.00 V. Orange: α spin density, purple: β spin density; isosurface value 0.003 a.u.; hydrogen atoms omitted for clarity.

The reduced complexes [Cr(tpe)_2_]^2+^,^[^ ^24^ ^]^ [Cr(tpe)(tpe^CF3^)]^2+^, *cis*‐[Cr(tpe^CF3^)_2_]^2+^, and *trans*‐[Cr(tpe^CF3^)_2_]^2+^ were optimized by DFT methods as well. The optimized triplet states were found to be more stable than the quintet states on this level of theory (Tables S6–S9). Hence, we will discuss the dications in their respective triplet states. The Mulliken spin densities at the chromium center are reduced from 3.2 in the trications to only 2.5 in [Cr(tpe)_2_]^2+^ and 2.6 in the CF_3_‐substituted dications (Tables S6–S9). This suggests an appreciable redox noninnocence of the pyridine ligands,^[^ ^24^, ^58^, ^59^, ^60^, ^61^ ^]^ which is more pronounced in the complexes with electron‐poor tpe^CF3^ ligand. The triplet states can thus largely be described as chromium(III) with a ligand radical that is antiferromagnetically coupled to the chromium center. The β spin density distribution in [Cr(tpe)_2_]^2+^ is delocalized over two *trans*‐coordinated coplanar pyridines of different tpe ligands (Figure 5).^[^ ^24^ ^]^ The Cr─N bonds to these reduced pyridines are shortened by 0.03 Å relative to those of the trication (Tables S6–S9). In the heteroleptic complex [Cr(tpe)(tpe^CF3^)]^2+^, the β spin density is localized on the CF_3_‐substituted pyridine (Figure 5), fully agreeing with the expectation. The Cr─N bond to this pyridine radical anion reduces by 0.08 Å relative to that of the trication (Tables S6–S9). In the doubly substituted complexes *cis*‐[Cr(tpe^CF3^)_2_]^2+^ and *trans*‐[Cr(tpe^CF3^)_2_]^2+^, the β spin density is distributed over both CF_3_‐substituted pyridines (Figure 5) and the Cr─N bond lengths to these are compressed by 0.04 Å (Tables S6–S9).

The strongly colored dicationic complexes [CrL_2_]^2+^ possess numerous strong absorption bands in the vis‐NIR spectral region (Figures S27–S30). These are qualitatively well reproduced by time‐dependent DFT (TD‐DFT) calculations. The intense transitions correspond to metal‐to‐ligand transitions according to the electron density difference maps (Figures S27–S30).

Gratifyingly, the DFT‐calculated [CrL_2_]^3+/2+^ redox potentials of the CF_3_‐substituted complexes relative to that of [Cr(tpe)_2_]^3+/2+^ (set to zero) correctly reproduce the observed experimental trend and are with Δ*E*_1/2_(DFT) = +0.12, +0.21 and + 0.25 V even very close to the experimentally determined ones with Δ*E*_1/2_(exp) = +0.11, +0.20 and + 0.22 V (see above, Figure 5 and Tables S6–S9). Consequently, our DFT calculations on the [CrL_2_]^3+/2+^ complexes are very reasonable.

In summary, the DFT and CASSCF‐NEVPT2 calculations reproduce the experimental data (redox potentials and excited state energies) in this series of CF_3_‐substituent complexes very well. This finding gives confidence that future quantum chemical calculations on substituted chromium(III) complexes will predict redox potentials and excited state energies of chromium(III) complexes sufficiently well. This will allow systematic computational screenings of chromium(III) chromophores.

## 3 Conclusion

The redox‐active polypyridine chromium(III) complex [Cr(tpe)_2_]^3+^ possesses favorable properties for photocatalysis, such as reversible one‐electron reduction, millisecond excited state lifetime and high stability.

Substituting [Cr(tpe)_2_]^3+^ with one or two CF_3_ groups in [Cr(tpe)(tpe^CF3^)]^3+^, *cis*‐[Cr(tpe^CF3^)_2_]^3+^ and *trans*‐[Cr(tpe^CF3^)_2_]^3+^ incrementally shifts the [Cr(tpe^R^)_2_]^3+/2+^ redox potential to more positive values. The energy and high lifetime of the emissive SF excited states remain, however, unchanged. This unusual decoupling of redox potential and excited state energy was achieved by localizing the redox process largely onto the CF_3_‐substituented pyridine ligands and confining the electronic excited state to the chromium center. Computational modeling of redox potentials and excited state energies confirmed the experimentally determined properties. The structure‐activity relationships identified here for chromium(III) complexes with tripodal ligands lay the foundation for computational property prediction and experimental realization of chromium(III)‐based photoredox catalysts.

## Supporting Information

The Supporting Information contains the employed methods, the synthesis procedures, processed spectroscopic, analytical, and computational data (pdf) and the Cartesian coordinates of DFT optimized geometries (xyz) available via the DOI https://doi.org/10.1002/chem.202502668. Deposition Number(s) 2479151 contain(s) the supplementary crystallographic data for this paper. These data are provided free of charge by the joint Cambridge Crystallographic Data Centre and Fachinformationszentrum Karlsruhe Access Structures service. All the raw data from this manuscript have been uploaded to Zenodo and are freely available via the DOI https://doi.org/10.5281/zenodo.16963566. The authors have cited additional references within the Supporting Information.^[^ ^71^, ^72^, ^73^, ^74^, ^75^, ^76^, ^77^, ^78^, ^79^, ^80^, ^81^, ^82^, ^83^, ^84^, ^85^, ^86^, ^87^, ^88^, ^89^, ^90^, ^91^, ^92^, ^93^, ^94^, ^95^, ^96^, ^97^, ^98^, ^99^ ^]^

## Conflict of Interest

The authors declare no conflict of interest.

## Supporting information



Supporting Information

Supporting Information

## Data Availability

The data that support the findings of this study are available in the supplementary material of this article and the raw data can be found under https://doi.org/10.5281/zenodo.16963566.
