# Supplementary material for: Decoupling Redox Potentials and Excited State Energies in Substituted Chromium(III) Chromophores
Source: Chemistry. 2025 Sep 27;32(28):e02668. doi: 10.1002/chem.202502668 (PMC13411421; doi:10.1002/chem.202502668)

checkCIF/PLATON report

Structure factors have been supplied for datablock(s) ak006

THIS REPORT IS FOR GUIDANCE ONLY. IF USED AS PART OF A REVIEW PROCEDURE FOR PUBLICATION, IT SHOULD NOT REPLACE THE EXPERTISE OF AN EXPERIENCED CRYSTALLOGRAPHIC REFEREE.

No syntax errors found.   CIF dictionary   Interpreting this report

Datablock: ak006

Bond precision: C-C = 0.0030 A   Wavelength=0.71073

Cell:	a=10.781(2)	b=13.484(3)	c=18.923(4)
	alpha=90	beta=102.31(3)	gamma=90
Temperature:	120 K		

	Calculated	Reported
Volume	2687.6(10)	2687.8(10)
Space group	P 21/c	P 21/c
Hall group	-P 2ybc	-P 2ybc
Moiety formula	C20 H15 Cr F9 N3 O9 S3	?
Sum formula	C20 H15 Cr F9 N3 O9 S3	C20 H15 Cr F9 N3 O9 S3
Mr	760.53	760.53
Dx,g cm-3	1.880	1.879
Z	4	4
Mu (mm-1)	0.778	0.778
F000	1524.0	1524.0
F000'	1527.91	
h,k,lmax	14,17,25	14,17,25
Nref	6570	6505
Tmin,Tmax	0.708,0.947	0.847,1.202
Tmin'	0.492	

Correction method= # Reported T Limits: Tmin=0.847 Tmax=1.202
AbsCorr = MULTI-SCAN

Data completeness= 0.990   Theta(max)= 28.129

R(reflections)= 0.0398(5368)   wR2(reflections)= 0.1075(6505)

S = 1.064   Npar= 553

The following ALERTS were generated. Each ALERT has the format
test-name_ALERT_alert-type_alert-level.
Click on the hyperlinks for more details of the test.

Alert level C

```
PLAT094_ALERT_2_C Ratio of Maximum / Minimum Residual Density ....       2.36 Report
PLAT220_ALERT_2_C NonSolvent   Resd 1  O   Ueq(max)/Ueq(min) Range        3.6 Ratio
PLAT234_ALERT_4_C Large Hirshfeld Difference Cr1      --O1A      .       0.19 Ang.
PLAT906_ALERT_3_C Large K Value in the Analysis of Variance ......      2.009 Check
```

Alert level G

```
PLAT002_ALERT_2_G Number of Distance or Angle Restraints on AtSite         32 Note
PLAT003_ALERT_2_G Number of Uiso or U(i,j)  Restrained non-H-Atoms         32 Report
PLAT063_ALERT_4_G Crystal Size Possibly too Large for Beam Size ..       0.90 mm
PLAT175_ALERT_4_G The CIF-Embedded .res File Contains SAME Records          2 Report
PLAT178_ALERT_4_G The CIF-Embedded .res File Contains SIMU Records          2 Report
PLAT187_ALERT_4_G The CIF-Embedded .res File Contains RIGU Records          2 Report
PLAT188_ALERT_3_G A Non-default SIMU Restraint Value has been used     0.0100 Report
PLAT189_ALERT_3_G A Non-default SAME Restraint Value for First Par     0.0010 Report
PLAT189_ALERT_3_G A Non-default SAME Restraint Value for First Par     0.0010 Report
PLAT190_ALERT_3_G A Non-default RIGU Restraint Value for First Par     0.0010 Report
PLAT242_ALERT_2_G Low    'MainMol' Ueq as Compared to Neighbors of        C20 Check
PLAT301_ALERT_3_G Main Residue  Disorder .............(Resd    1)         36% Note
PLAT432_ALERT_2_G Short Inter X...Y Contact  F4A      ..C14      .       2.78 Ang.
                                                 1-x,-y,1-z  =      3_656 Check
PLAT432_ALERT_2_G Short Inter X...Y Contact  F5A      ..C4       .       2.84 Ang.
                                             x,1/2-y,-1/2+z  =      4_565 Check
PLAT432_ALERT_2_G Short Inter X...Y Contact  F6A      ..C15      .       2.34 Ang.
                                                 1-x,-y,1-z  =      3_656 Check
PLAT480_ALERT_4_G Long H...A H-Bond Reported H2       ..O9       .       2.61 Ang.
PLAT480_ALERT_4_G Long H...A H-Bond Reported H9       ..S3       .       3.01 Ang.
PLAT480_ALERT_4_G Long H...A H-Bond Reported H12      ..O2       .       2.61 Ang.
PLAT480_ALERT_4_G Long H...A H-Bond Reported H12      ..S1A      .       3.00 Ang.
PLAT480_ALERT_4_G Long H...A H-Bond Reported H17      ..O6       .       2.62 Ang.
PLAT793_ALERT_4_G Model has Chirality at C6         (Centro SpGr)           S Verify
PLAT811_ALERT_5_G No ADDSYM Analysis: Too Many Excluded Atoms ....          ! Info
PLAT860_ALERT_3_G Number of Least-Squares Restraints ............         682 Note
PLAT883_ALERT_1_G Absent Datum for _atom_sites_solution_primary ..     Please Do !
PLAT910_ALERT_3_G Missing FCF Reflection(s) Below Theta(Min)[Deg]=       2.45 Note
                1  0  0,   0  1  1,   0  0  2,
PLAT912_ALERT_4_G Missing # of FCF Reflections Above STh/L=  0.600         59 Note
PLAT933_ALERT_2_G Number of HKL-OMIT Records in Embedded .res File          1 Note
                3  6  2,
PLAT955_ALERT_1_G Reported (CIF) and Actual (FCF) Lmax Differ by .          1 Units
PLAT969_ALERT_5_G The 'Henn et al.' R-Factor-gap value ..........       3.699 Note
              Predicted wR2: Based on SigI**2  2.91 or SHELX Weight 10.11
PLAT978_ALERT_2_G Number C-C Bonds with Positive Residual Density.          7 Info
```

0 **ALERT level A** = Most likely a serious problem - resolve or explain
0 **ALERT level B** = A potentially serious problem, consider carefully
4 **ALERT level C** = Check. Ensure it is not caused by an omission or oversight
30 **ALERT level G** = General information/check it is not something unexpected

```
 2 ALERT type 1 CIF construction/syntax error, inconsistent or missing data
10 ALERT type 2 Indicator that the structure model may be wrong or deficient
 8 ALERT type 3 Indicator that the structure quality may be low
12 ALERT type 4 Improvement, methodology, query or suggestion
 2 ALERT type 5 Informative message, check
```

It is advisable to attempt to resolve as many as possible of the alerts in all categories. Often the minor alerts point to easily fixed oversights, errors and omissions in your CIF or refinement strategy, so attention to these fine details can be worthwhile. In order to resolve some of the more serious problems it may be necessary to carry out additional measurements or structure refinements. However, the purpose of your study may justify the reported deviations and the more serious of these should normally be commented upon in the discussion or experimental section of a paper or in the "special_details" fields of the CIF. checkCIF was carefully designed to identify outliers and unusual parameters, but every test has its limitations and alerts that are not important in a particular case may appear. Conversely, the absence of alerts does not guarantee there are no aspects of the results needing attention. It is up to the individual to critically assess their own results and, if necessary, seek expert advice.

Publication of your CIF in IUCr journals

A basic structural check has been run on your CIF. These basic checks will be run on all CIFs submitted for publication in IUCr journals (*Acta Crystallographica*, *Journal of Applied Crystallography*, *Journal of Synchrotron Radiation*); however, if you intend to submit to *Acta Crystallographica Section C* or *E* or *IUCrData*, you should make sure that full publication checks are run on the final version of your CIF prior to submission.

Publication of your CIF in other journals

Please refer to the *Notes for Authors* of the relevant journal for any special instructions relating to CIF submission.

PLATON version of 04/06/2025; check.def file version of 30/05/2025

Datablock ak006 - ellipsoid plot

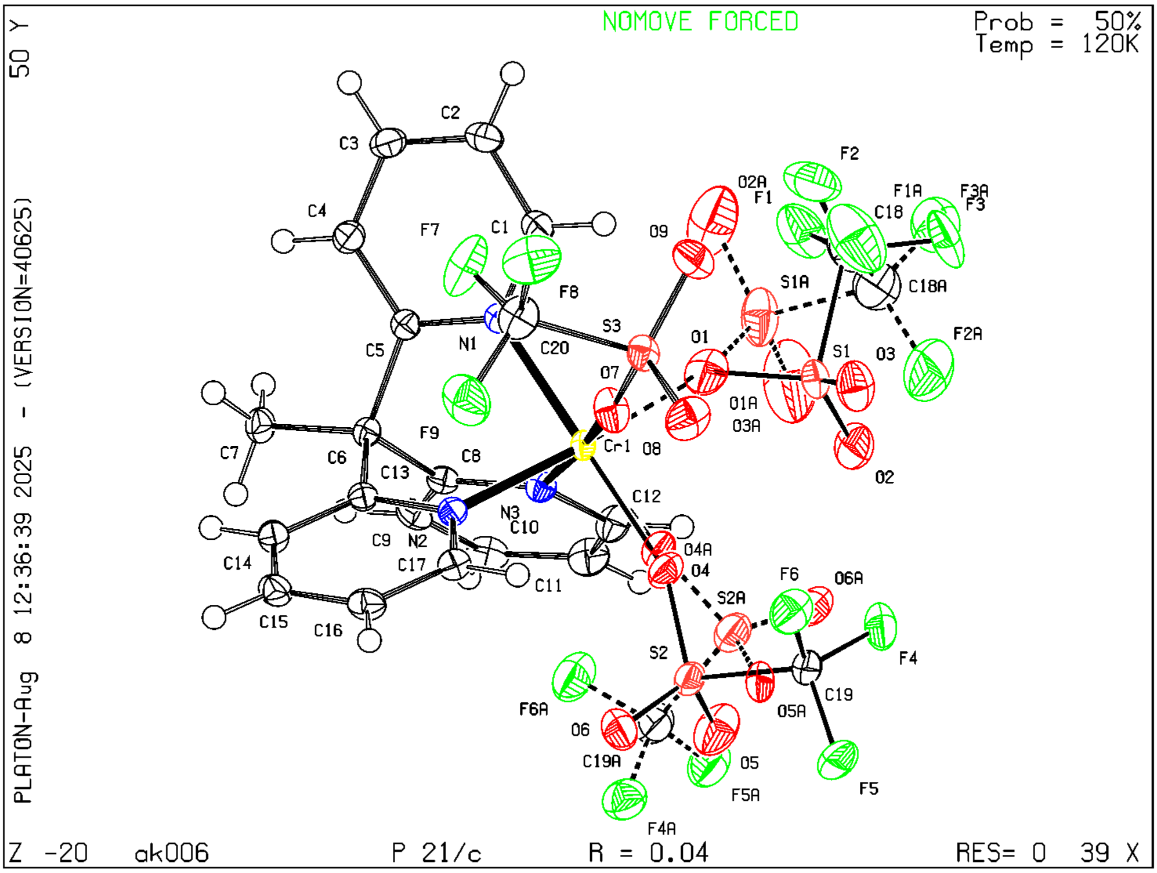

Supplement: Supplementary file 1 — Supporting Information [file CHEM-32-e02668-s001.zip › checkcif.pdf]
