# Supplementary material for: Decoupling Redox Potentials and Excited State Energies in Substituted Chromium(III) Chromophores
Source: Chemistry. 2025 Sep 27;32(28):e02668. doi: 10.1002/chem.202502668 (PMC13411421; doi:10.1002/chem.202502668)
Supplement: Supplementary file 2 — Supporting Information [file CHEM-32-e02668-s002.pdf]

Decoupling Redox Potentials and Excited State Energies in Substituted Chromium(III) Chromophores

Steven Sittel, Dimitri Zorn, Alexandra König, Jonas Marcel Grenz, Christoph Förster, Robert Naumann and Katja Heinze

Supporting Information

General. [Cr(tpe)$_2$][PF$_6$]$_3$,[24-26] Cr[OTf]$_3$[44] and 1,1-bis(2-pyridyl)ethane[40] were synthesized according to literature procedures. 2-Fluoro-4-(trifluoromethyl)pyridine (Angene Chemical, 98%), CrCl$_3$×6H$_2$O (Alfa Aesar, >99.5%), [n-Bu$_4$N][PF$_6$] for synthesis (>98%, TCI), trimethylsilyl trifluoromethanesulfonate (TMSOTf; TCI, >98%), NaCl (Thermo Fisher Scientific, ≥99.5%), cobaltocene (Sigma-Aldrich), ferrocenium hexafluorophosphate (Sigma-Aldrich), methanol, isopropanol, acetonitrile, diethylether, bis(2-methoxyethyl)ether, acetonitrile for spectroscopy (Supelco, hypergrade), water, CD$_3$CN (Deutero®) were commercially available and used as received. [n-Bu$_4$N][PF$_6$] for electrochemical experiments (≥99% for electrochemical analysis, Sigma-Aldrich) was dried at 80 °C at reduced pressure (10^{-3} mbar) for three days and stored under argon. Syntheses and handling of air-sensitive compounds were either conducted using Schlenk techniques or a glovebox (UniLab/Mbraun – Ar 5.0; O$_2$ <0.1 ppm; H$_2$O <0.1 ppm). Microwave syntheses were conducted using a CEM Discover microwave in 10 mL glass vials with Teflon seal caps under stirring.

Intensity data for crystal structure determination of Cr(OTf)$_3$(tpe) were collected with a STOE IPDS-2T diffractometer from STOE & CIE GmbH with an Oxford cooling using Mo-K$_\alpha$ radiation (λ = 0.71073 Å). The diffraction frames were integrated using the STOE X-Area[71] software package and were corrected for absorption with MULABS[72] of the PLATON software package.[73] The structures were solved with SHELXT[74] and refined by the full-matrix method based on F^2 using SHELXL[75] of the SHELX[76] software package and the ShelXle[77] graphical interface. All non-hydrogen atoms were refined anisotropically, while the positions of all hydrogen atoms were generated with appropriate geometric constraints and allowed to ride on their respective parent atoms with fixed isotropic thermal parameters. Crystallographic data for the structure reported in this paper have been deposited with the Cambridge Crystallographic Data Centre as supplementary publication no. CCDC-2479151.

Crystallographic data of Cr(OTf)$_3$(tpe): C$_{20}$H$_{15}$CrF$_9$N$_3$O$_9$S$_3$ (760.53); monoclinic; $P2_1/c$, a = 10.781(2) Å, b = 13.484(3) Å, c = 18.923(4) Å, β = 102.31(3)°; V = 2687.8(10) Å^3, Z = 4; density (calculated) = 1.879 g cm^{-3}; T = 120(2) K; μ = 0.778 mm^{-1}; F(000) = 1524; crystal size 0.900 × 0.380 × 0.070 mm^3; θ = 2.454 to 28.129 deg.; –14≤h≤14, –17≤k≤17, –25≤l≤24; rfln collected = 32444; rfln unique = 6505 [R(int) = 0.0613]; completeness to θ = 25.242 deg. = 99.9 %; semi empirical absorption correction from equivalents; max. and min. transmission 1.20201 and 0.84693; Data 6505; restraints 682; parameters 553; goodness-of-fit on F^2 = 1.064; final indices [$I > 2\sigma(I)$] R_1 = 0.0398, wR_2 = 0.1002; R indices (all data) R_1 = 0.0522, wR_2 = 0.1075; largest diff. peak and hole 1.051 and –0.445 e Å^{-3}.

HPLC purification and analysis was carried out using a Jasco LC-NetII/ADC control unit, two Jasco PU-2087 Plus pumps, a Jasco CO-4060 column oven and a Jasco UV-2075 Plus absorption detector. Fractions were collected using an Advantec CHF122SC fraction collector. Eluents were degassed by a Gastorr AG-42 degassing unit and subsequently mixed with a high-pressure mixing unit. Analytical runs were conducted on a Macherey-Nagel EC Nucleodur HILIC column (5 µm, 4.6 × 250 mm), while semipreparative separations were conducted on a Macherey-Nagel VP Nucleodur HILIC column (5 µm, 21 × 250 mm). Discrimination between *cis* and *trans* isomers was achieved using a chiral column ChiralPAK IC (5 µm, 4.6 × 250 mm) column from Daicel.

Electrochemical experiments were carried out on a BioLogic SP-200 voltammetric analyzer. Platinum wires were used as working and counter electrodes and 10 mM $Ag/AgNO_3$ (100 mM [*n*-Bu_4N][ClO_4] in MeCN) was used as the reference electrode. For [Cr(tpe)$_2$][PF_6]$_3$, a glassy carbon working electrode was used. 100 mM [*n*-Bu_4N][PF_6] as supporting electrolyte in MeCN and 1 mM of the sample were used. Cyclic voltammetry experiments were carried out at scan rates of 50–100 mV s^{-1}. Potentials were referenced relative to the ferrocenium/ferrocene couple. Potentials were converted to the saturated calomel electrode (SCE).[63]

IR spectra were recorded with a Bruker Alpha II FTIR spectrometer with an ATR unit containing a diamond crystal. The intensities are qualitatively indicated with weak (w), medium (m) and strong (s). Assignments ware accomplished according to the literature.[78]

NMR spectra were recorded on a Bruker Avance II 400 spectrometer at 400.13 MHz (^{1}H), 100.70 MHz (^{13}C{^{1}H}) or 376.50 MHz (^{19}F). Resonances are reported in ppm versus the solvent signal as an internal standard (^{1}H/^{13}C: δ(CD_3CN) = 2.13, 1.94/118.26, 1.32).[79] ^{19}F NMR resonances are reported in ppm versus $CFCl_3$ (δ($CFCl_3$) = 0) as external standard. (s) = singlet, (d) = doublet, (t) = triplet, (m) = multiplet.

ESI$^+$ mass spectra were measured on an Agilent 6545 HPLC-ESI-QTOF-MS spectrometer in MeCN.

UV/vis absorption spectra were recorded on an Agilent Cary 5000 UV/vis/NIR spectrophotometer using 1.0 cm quartz cells. 1.00 cm quartz cells with a Schott valve were used to maintain an inert atmosphere.

Emission spectra and luminescence decay curves were recorded with a FLS1000 spectrometer from Edinburgh Instruments equipped with the cooled red and NIR sensitive photomultiplier detectors PMT-980 and N-G09 PMT-1700, together covering the entire spectral range between 200 nm and 1700 nm. A xenon arc lamp Xe2 (450 W) was used for excitation in steady-state measurements. Time-resolved luminescence experiments were performed in the multi-channel scaling mode employing a variable pulsed laser VPL-450 (λ_{exc} = 451.3 nm) as excitation source. The absolute luminescence quantum yield Φ was determined using an integrating sphere from Edinburgh Instruments. Relative uncertainty of Φ is estimated to be ±10%. Due to the extremely long excited state lifetimes in the millisecond range and the high excited state redox potentials of the complexes, their luminescence is quenched efficiently even by trace impurities. Therefore, it is very challenging to reproducibly obtain the natural lifetime τ_0. Highly purified samples and a particularly cautious conduction of the experiments is

mandatory, otherwise erroneous τ_0 values much lower than milliseconds are obtained. Trace impurities that quench the emission will also affect the luminescence quantum yield Φ, which is proportional to the lifetime τ according to $\Phi = k_r\tau$.

Density functional theory and CASSCF-NEVPT2 calculations were carried out using the ORCA program package 5.0.3.[80,81]

The **DFT** calculations were performed using the B3LYP functional[82,83] employing the RIJCOSX approximation[84,85] and the SARC/J auxillary basis.[86] Tight convergence criteria were chosen for DFT-UKS calculations (keywords *tightscf* and *tightopt*). Relativistic effects were calculated at the zeroth order regular approximation (ZORA) level.[86] The ZORA keyword automatically invokes relativistically adjusted basis sets. To account for solvent effects, a conductor-like screening model (CPCM) modeling acetonitrile was used in all calculations.[87,88] Geometry optimizations were performed using Ahlrichs' polarized valence triple-ζ basis set (def2-TZVPP).[89,90] Atompairwise dispersion correction was performed with the Becke-Johnson damping scheme (D3BJ).[91,92] The energy of the electronic states and the presence of energy minima were checked by numerical frequency calculations. Explicit counter ions and/or solvent molecules were not taken into account. TD-DFT calculations were performed at the same level of theory. Fifty vertical spin-allowed transitions were calculated.

The **CASSCF(7,12)-SC-NEVPT2** calculations of ground and excited state properties with respect to metal-centered (MC) states were performed using the complete-active-space self-consistent field method (CASSCF).[93,94] To accelerate the calculation, the RI-JK approximation was used,[95,96] with an automatically generated auxillary basis (*AutoAux*). To recover the missing dynamic electron correlation, the strongly contracted variant of N-electron valence perturbation theory to second order (SC-NEVPT2) was used.[97,98] All electronic states are classified by irreducible representations of the *O* point group, despite the lower actual symmetry of the considered complex. To accurately model the ligand field, the active space was expanded to encompass the dominant bonding/antibonding orbitals formed between chromium and the ligand. In addition to the minimal active space of (3,5), two occupied Cr–N σ bonding orbitals and a second d shell[99] were included in these calculations giving an active space of (7,12). 10 quartet and 10 doublet roots were calculated with this active space.

Synthesis of the tpe^{CF3} ligand

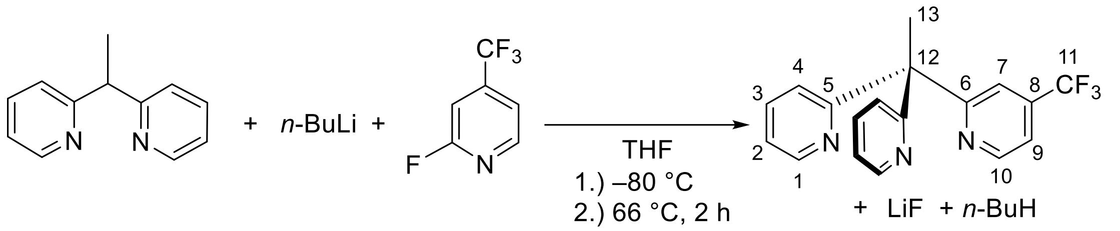

The following procedure was carried out under an inert atmosphere until the quenching step. Glassware was oven-dried and flushed with inert gas.

In a 250 mL three-neck round-bottom flask, 1,1-bis(2-pyridyl)ethane (5.0 g, 27 mmol, 1.0 eq.) was dissolved in dry THF (100 mL). The solution was cooled to –78 °C using an ethanol/dry ice bath. *n*-Butyllithium (1.8 g, 29 mmol, 12 mL of 2.5 M solution in hexanes, 1.1 eq.) was added dropwise to this solution, yielding a red solution. After addition, the mixture was stirred for 30 min at –78 °C. 2-Fluoro-4-(trifluoromethyl)pyridine (1.3 g, 30 mmol, 1.1 eq.) was dissolved in dry THF (20 mL) and added to the reaction solution over the course of 5 min, which led to a deeper red color. The reaction mixture was allowed to reach to room temperature and refluxed for 2 h. The red solution was cooled to room temperature and poured onto ice (100 g), causing a color change to yellow. The organic solvents were removed by rotary evaporation. The remaining aqueous phase was filtered under reduced pressure to remove precipitated LiF and then extracted with diethyl ether (4×100 mL). The combined organic extracts were dried over anhydrous sodium sulfate, filtered and the solvent was removed by rotary evaporation. The residue was dried under reduced pressure (10^{-3} mbar) and the product was obtained as a yellow oil (8.1 g, 25 mmol, 91 %). Upon standing at room temperature for three days, large colorless crystals formed from the oil. The crystals were washed with hexanes (4×5 mL) and dried under reduced pressure (3.2 g, 9.7 mmol, 36 %).

^{1}H NMR (400 MHz, CD_3CN): δ / ppm = 8.71 (1H, dt, $^3J_{HH}$ = 5.2 Hz, $^5J_{HH}$ = 0.8 Hz, H^{10}), 8.48 (2H, ddd, $^3J_{HH}$ = 4.8 Hz, $^4J_{HH}$ = 1.9 Hz ,$^5J_{HH}$ = 1.0 Hz, H^1), 7.67 (2H, ddd, $^3J_{HH}$ = 8.0 Hz, $^3J_{HH}$ = 7.5 Hz ,$^4J_{HH}$ = 1.9 Hz, H^3), 7.47 (1H, m, H^9), 7.35 (1H, m, H^7), 7.21 (2H, ddd, $^3J_{HH}$ = 7.5 Hz, $^3J_{HH}$ = 4.5 Hz ,$^4J_{HH}$ = 1.1 Hz, H^2), 7.14 (2H, ddd, $^3J_{HH}$ = 8.0 Hz, $^4J_{HH}$ = 1.1 Hz, H^4), 2.25 (3H, s, H^{13}).

$^{13}C\{^1H\}$ NMR (100 MHz, CD_3CN): δ / ppm = 168.83 (s, C^6), 166.21 (s, C^5), 150.51 (s, C^{10}), 149.51 (s, C^1), 137.28 (s, C^3), 124.35 (s, C^4), 122.66 (s, C^2), 120.40 (s, C^7), 117.83 (s, C^9), 61.27 (s, C^{12}), 27.54 (s, C^{13}). The ^{13}C NMR resonance of C^{11} was not detected.

^{19}F NMR (377 MHz, CD_3CN): δ / ppm = –65.28 (s).

MS (ESI$^+$, MeCN): *m*/*z* (%) = calcd for [tpe^{CF3}+H; $C_{18}H_{15}F_3N_3$]$^+$: *m*/*z* = 330.121; found: *m*/*z* (%) = 330.121 (100) [M+H]$^+$.

IR (ATR): $\tilde{\nu}$ / cm^{-1} = 3067 (w, CH), 3012 (w, CH), 2995 (w, CH), 2940 (w, CH), 1583 (m), 1567 (m), 1472 (m), 1430 (m), 1390 (m), 1329 (s, CF), 1293 (m), 1239 (w), 1181 (s), 1131 (s, CF), 1088 (m), 1065 (m), 1046 (m), 990 (m), 912 (w), 896 (w), 874 (m), 843 (m), 800 (w), 785 (m), 769 (w), 750 (s), 727 (w), 665 (s), 631 (w), 620 (m), 611 (m), 587 (w), 570 (m), 525 (w), 512 (w), 472 (w), 455 (w), 416 (w), 402 (w).

Synthesis of $CrCl_3$(tpe)

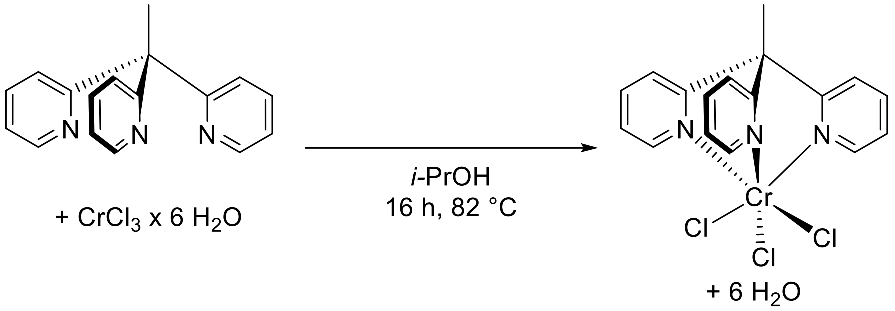

The precursor complex $CrCl_3$(tpe) was prepared analogously to the preparation of $CrCl_3$(tpy) (tpy =2,2ʻ:6ʻ,2ʻʻ-terpyridine).[47] $CrCl_3 \times 6$ H_2O (2.14 g, 8.06 mmol, 1.05 eq.) was dissolved in isopropanol (50 mL). To the resulting deep green solution was added a pale-yellow solution of tpe (2.00 g, 7.66 mmol, 1.00 eq.) in isopropanol (50 mL). The mixture was heated to 82 °C for 16 h. A fine green solid precipitated. The suspension was cooled to room temperature and filtered under reduced pressure. The remaining green solid was washed with warm isopropanol (60 °C; 3×50 mL), acetonitrile (2×50 mL) and diethyl ether (2×50 mL). The obtained green powder (2.06 g, 4.94 mmol, 64 %) was dried under reduced pressure and used without further purification.

Synthesis of $Cr(OTf)_3(tpe)$

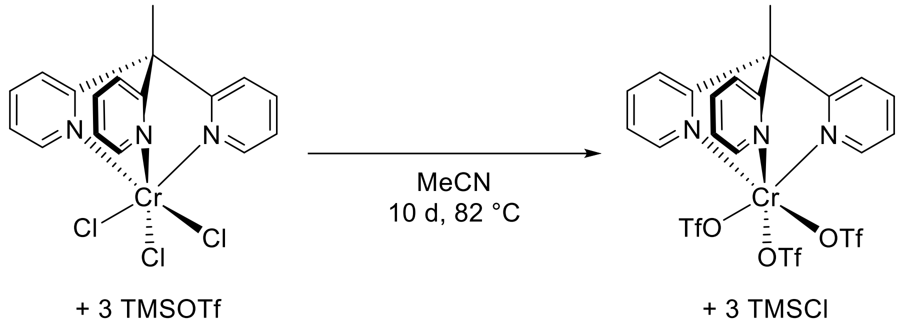

The precursor complex $Cr(OTf)_3(tpe)$ was prepared analogously to the preparation of $Cr(ddpd)(OTf)_3$ (ddpd = *N*,*N*′-dimethyl-*N*,*N*′-dipyridine-2-yl-pyridine-2,6-diamine).[49] All steps were carried out using dry solvents and inert conditions using Schlenk techniques or a glovebox. $CrCl_3(tpe)$ (2.26 g, 3.02 mmol, 1.00 eq.) was suspended in MeCN (50 mL) and TMSOTf (10.0 g, 45.0 mmol, 15.0 eq.) was added. The green suspension immediately became purple-brown and the green solid partially dissolved. The mixture was heated to 82 °C for ten days and then cooled to room temperature.[a] The solvent and the TMSCl byproduct were removed under reduced pressure at 40 °C until a dark-purple residue remained. The crude product was further dried under reduced pressure for 16 h, washed with diethyl ether (2×50 mL), petroleum ether (2×50 mL) and ground using a spatula. The residue was dissolved in 10 mL MeCN and the purple product was precipitated via slow addition of diethyl ether (120 mL).[b] The supernatant was decanted and the purple solid was washed with MeOH (2×20 mL), petroleum ether (2×20 mL) and diethyl ether (20 mL). After drying for 16 h under reduced pressure, the product was obtained as a purple solid and used without further purification (1.11 g, 1.46 mmol, 49 %). Dark purple single crystals suitable for XRD analysis were obtained via diffusion of diethyl ether into a solution of $Cr(OTf)_3(tpe)$ in MeCN (50 mg/5 mL).

[a] *Before proceeding with the workup, it is recommended to grease the stopcock of the flask again, since it is prone to getting stuck.*

[b] *Occasionally, the precipitate clotted again during this process. Washing the residue with diethyl ether and repeating the precipitation proved useful in these cases.*

Synthesis of $[Cr(tpe)(tpe^{CF3})][PF_6]_3$

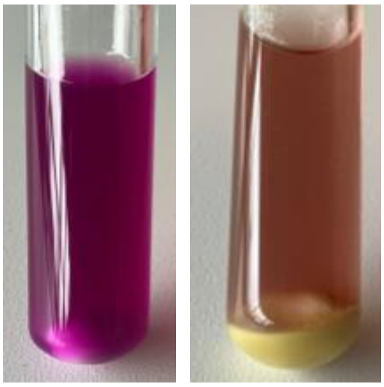

before (left) and after (right) the microwave reaction.

In a glovebox, $Cr(OTf)_3(tpe)$ (100 mg, 0.13 mmol, 1.0 eq.) was dissolved in dry, deaerated MeCN (0.5 mL), resulting in a purple solution. A colorless solution of tpe^{CF3} (52.0 mg, 0.16 mmol, 1.2 eq.) in bis(2-methoxyethyl)ether (diglyme, 4 mL) was added. The purple mixture (see photo) was transferred to a microwave vial and heated in a microwave oven at 200 °C for 3 h. A yellow solid with an orange supernatant was obtained (see photo). The liquid was decanted and discarded. The solid was washed with diethyl ether (4×5 mL) and dried under ambient conditions. The product was obtained as a pale-yellow powder (128 mg, 0.11 mmol, 89 % yield). As determined by analytical HPLC, the crude product was contaminated with $[Cr(tpe)_2]^{3+}$ and presumably $[Cr(tpe^{CF3})_2]^{3+}$ (Figure S9).

The desired heteroleptic complex (several combined batches) was purified by HPLC (H_2O (300 mM NaCl)/MeCN (40/60 *v*/*v*); R_t = 6.8 min). The solvent mixture was removed by rotary evaporation. To separate the product from NaCl, the solid residue was extracted with MeCN (4×50 mL). The solvent was removed by rotary evaporation. The remaining pale-yellow solid was dissolved in MeOH (5 mL) and added dropwise to a saturated solution of $[n\text{-}Bu_4N][PF_6]$ in MeOH (3 mL) under strong stirring, yielding $[Cr(tpe)(tpe^{CF3})][PF_6]_3$ as a fine precipitate. The solid was washed with MeOH (5×10 mL) and diethyl ether (5 mL) and dried under ambient conditions. Finally, the product was dissolved in MeCN (3 mL) and crystallized by diffusion of diethyl ether. Crude $[Cr(tpe)(tpe^{CF3})_2][OTf]_3$ (680 mg) yielded purified $[Cr(tpe)(tpe^{CF3})][PF_6]_3$ (432 mg).

MS (MeCN/ESI$^+$): calcd for $[[Cr(tpe)(tpe^{CF3})][PF_6]_2]^+$: m/z = 932.108, found: m/z (%) = 932.108 (77.0); calcd for $[[Cr(tpe)(tpe^{CF3})][PF_6]]^{2+}$: m/z = 393.572, found: m/z (%) = 393.571 (100); calcd for $[[Cr(tpe)(tpe^{CF3})]]^{3+}$: m/z = 214.060, found: m/z (%) = 214.061 (20.2).

IR (ATR): $\tilde{\nu}$ / cm^{-1} = 3145 (w, CH), 3103 (w, CH), 1663 (w), 1631 (w,), 1605 (w), 1579 (w), 1496 (w), 1472 (w) 1442 (w), 1413 (w), 1392 (w), 1335 (w, CF), 1307 (w), 1249 (w), 1210 (w), 1188 (w), 1159 (w, CF), 1113 (w), 1062 (w), 1037 (w), 874 (m), 826 (vs, PF), 757 (m), 723 (w), 685 (w), 662 (w), 640 (w), 619 (w), 556 (vs, PF), 509 (w), 465 (w), 445 (w), 411 (w).

UV/vis/NIR absorption (MeCN): λ / nm (ε / M^{-1} cm^{-1}) = 332 (1200), 433 (35).

Emission (MeCN): λ / nm (I_{rel}) = 719 (0.50), 723 (0.53), 730 (0.75), 743 (1.00), 748 (0.90), 767 (0.28), 790 (0.23), 818 (0.10), 826 (0.11).

Emission Lifetime (MeCN): τ / µs = 2600 (deaerated); 330 (aerated).

Quantum Yield (MeCN): Φ / % = 4.9.

CV (MeCN, 100 mM $[n\text{-}Bu_4N][PF_6]$): $E_{1/2}$ / V vs. SCE = –0.40 V, –1.06 V.

Synthesis of $[Cr(tpe^{CF3})_2]^{3+}$

$Cr(OTf)_3$ (200 mg, 0.40 mmol, 1.0 eq.) was dissolved in MeCN (0.5 mL) in a glovebox, resulting in a turquoise solution. A colorless solution of tpe^{CF3} (277 mg, 0.84 mmol, 2.1 eq.) in bis(2-methoxyethyl)ether (diglyme, 4 mL) was added. The turquoise mixture (see photo) was transferred to a microwave vial and heated in a microwave oven at 200 °C for 3 h. A yellow solid with an orange supernatant was obtained (see photo). The liquid was decanted and discarded. The solid was washed with diethyl ether (4×5 mL) and dried under ambient conditions. The crude product was obtained as a pale-yellow powder (329 mg, 0.28 mmol, 71 % yield). As determined by analytical HPLC, the mixture consisted of *cis*-$[Cr(tpe^{CF3})_2]^{3+}$ and *trans*-$[Cr(tpe^{CF3})_2]^{3+}$ in a 2:1 ratio (Figure S11).

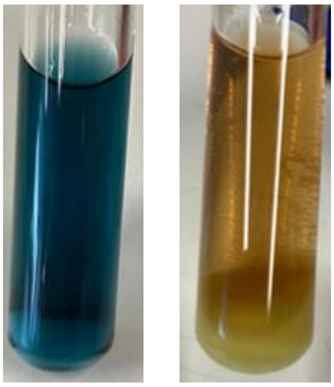

before (left) and after (right) the microwave reaction.

The isomers (several combined batches) were separated by HPLC (H_2O (75 mM NaCl)/MeCN (17.5/82.5 *v*/*v*); R_t(*trans*) = 28.9 min; R_t(*cis*) = 31.6 min). For both fractions, the solvent mixture was removed by rotary evaporation. To separate the product from NaCl, the solid residue was extracted with MeCN (4×50 mL). The solvent was removed by rotary evaporation. The remaining pale-yellow solid was dissolved in MeOH (5 mL) and added dropwise to a saturated solution of $[n\text{-}Bu_4N][PF_6]$ in MeOH (3 mL) under strong stirring, yielding the respective isomer as a fine precipitate. The solid was washed with MeOH (5×10 mL) and diethyl ether (5 mL) and dried under ambient conditions. Finally, the product was dissolved in MeCN (3 mL) and crystallized by diffusion of diethyl ether. Crude $[Cr(tpe^{CF3})_2][OTf]_3$ (632 mg) yielded *cis*-$[Cr(tpe^{CF3})_2][PF_6]_3$ (180 mg) and *trans*-$[Cr(tpe^{CF3})_2][PF_6]_3$ (139 mg), respectively.

***cis*-[Cr(tpe^{CF3})$_2$][PF$_6$]$_3$:**

MS (MeCN/ESI$^+$): calcd for [[Cr(tpe)(tpe^{CF3})][PF$_6$]$_2$]$^+$: *m/z* = 1000.096, found: *m/z* (%) = 1000.098 (42.5); calcd for [[Cr(tpe)(tpe^{CF3})][PF$_6$]]$^{2+}$: *m/z* = 427.566, found: *m/z* (%) = 427.566 (100); calcd for [[Cr(tpe)(tpe^{CF3})]]$^{3+}$: *m/z* = 236.722, found: *m/z* (%) = 236.722 (64.6).

IR (ATR): $\tilde{\nu}$ / cm^{-1} = 3147 (w, CH), 3104 (w, CH), 1633 (w,), 1605 (w), 1578 (w), 1498 (w), 1474 (w) 1444 (w), 1412 (w), 1395 (w), 1334 (m, CF), 1299 (w), 1243 (w), 1213 (w), 1184 (m), 1144 (m, CF), 1116 (w), 1090 (w), 1073 (w), 1063 (w), 1037 (w), 878 (m), 824 (vs, PF), 774 (m), 759 (m), 741 (w), 724 (w), 684 (m), 666 (w), 642 (w), 620 (w), 596 (w), 555 (vs, PF), 509 (w), 489 (w), 463 (w), 446 (w), 439 (w), 424 (w), 408 (w).

UV/vis/NIR absorption (MeCN): λ / nm (ε / M^{-1} cm^{-1}) = 333 (1200), 432 (35).

Emission (MeCN): λ / nm (I_{rel}) = 719 (0.51), 730 (0.78), 738 (0.75), 743 (1.00), 748 (0.95), 768 (0.27), 790 (0.22), 819 (0.10), 828 (0.10).

Emission Lifetime (MeCN): τ / µs = 3000 (deaerated), 360 (aerated).

Quantum Yield (MeCN): Φ / % = 6.0.

CV (MeCN, 100 mM [*n*-Bu$_4$N][PF$_6$]): $E_{1/2}$ / V vs. SCE = –0.30 V, –0.90 V.

***trans*-[Cr(tpe^{CF3})$_2$][PF$_6$]$_3$:**

MS (MeCN/ESI$^+$): calcd for [[Cr(tpe)(tpe^{CF3})][PF$_6$]$_2$]$^+$: *m/z* = 1000.096, found: *m/z* (%) = 1000.097 (32.5); calcd for [[Cr(tpe)(tpe^{CF3})][PF$_6$]]$^{2+}$: *m/z* = 427.566, found: *m/z* (%) = 427.566 (100); calcd for [[Cr(tpe)(tpe^{CF3})]]$^{3+}$: *m/z* = 236.722, found: *m/z* (%) = 236.725 (32.6).

IR (ATR): $\tilde{\nu}$ / cm^{-1} = 3145 (w, CH), 3102 (w, CH), 1635 (w,), 1605 (w), 1590 (w), 1500 (w), 1472 (w) 1441 (w), 1414 (w), 1337 (w, CF), 1301 (w), 1245 (w), 1210 (w), 1179 (w), 1149 (m, CF) 1117 (w), 1090 (w), 1070 (w), 1064 (w), 1037 (w), 877 (m), 828 (vs, PF), 772 (m), 759 (m), 742 (w), 724 (w), 687 (m), 666 (w), 643 (w), 621 (w), 596 (w), 556 (vs, PF), 515 (w), 468 (w), 448 (w), 432 (w).

UV/vis/NIR absorption (MeCN): λ / nm (ε / M^{-1} cm^{-1}) = 333 (1300), 432 (35).

Emission (MeCN): λ / nm (I_{rel}) = 731 (0.76), 744 (0.98), 748 (1.00), 791 (0.21), 819 (0.09), 826 (0.10).

Emission Lifetime (MeCN): τ / µs = 3300 (deaerated), 360 (aerated).

Quantum Yield (MeCN): Φ / % = 6.2.

CV (MeCN, 100 mM [*n*-Bu$_4$N][PF$_6$]): $E_{1/2}$ / V vs. SCE = –0.28 V, –1.00 V.

Chemical reduction of chromium(III) complexes

Reduction of the chromium(III) complexes was accomplished in a glovebox using cobaltocene in dry and degassed MeCN.

In an exemplary procedure, a stock solution of $[Cr(tpe)_2][PF_6]_3$ (3.30 mg, 3.27 mmol) was prepared in MeCN (1.9 mL, 1.72 mmol L^{-1}). An aliquot of this stock solution (100 µL) was diluted with MeCN (2.9 mL) in an inert gas cuvette yielding a 0.06 mM solution. Separately, a cobaltocene stock solution (13.1 mg) was prepared in MeCN (25 mL, 2.74 mmol L^{-1}). An aliquot of the cobaltocene stock solution (64 µL) was added to the cuvette (0.06 mM in the cuvette) to reduce the chromium(III) complex. The solution immediately turned green. The cuvette was locked, removed from the glovebox and UV/vis/NIR absorption spectra were recorded.

UV/vis/NIR absorption of $[Cr(tpe)_2][PF_6]_2$ (MeCN): λ / nm (ε / M^{-1} cm^{-1}) = 416 (6000), 689 (6700), 890 (13400, sh), 944 (25000).

UV/vis/NIR absorption of $[Cr(tpe)(tpe^{CF3})][PF_6]_2$ (MeCN): λ / nm (ε / M^{-1} cm^{-1}) = 436 (4800), 685 (3600), 795 (4200, sh), 983 (17800).

UV/vis/NIR absorption of *cis*-$[Cr(tpe^{CF3})_2][PF_6]_2$ (MeCN): λ / nm (ε / M^{-1} cm^{-1}) = 432 (5200), 690 (3500), 830 (6000, sh), 915 (10200, sh), 994 (19000).

UV/vis/NIR absorption of *trans*-$[Cr(tpe^{CF3})_2][PF_6]_2$ (MeCN): λ / nm (ε / M^{-1} cm^{-1}) = 437 (4900), 660 (3800), 761 (3600), 910 (5000, sh), 1048 (26700).

Reoxidation of reduced complexes

Reductions of the chromium(III) complexes were carried out as described above. For re-oxidation, a stock solution of ferrocenium hexafluorophosphate (10.4 mg) in MeCN (10 mL, 3.14 mmol L^{-1}) was prepared. Exemplary, the solution of $[Cr(tpe)_2][PF_6]_3$ in MeCN was treated with an aliquot of the ferrocenium hexafluorophosphate solution (57 µL) in the cuvette (0.06 mM in the cuvette). The cuvette was locked, removed from the glovebox and UV/vis/NIR absorption spectra were recorded. These matched the spectra of the tricationic complexes.

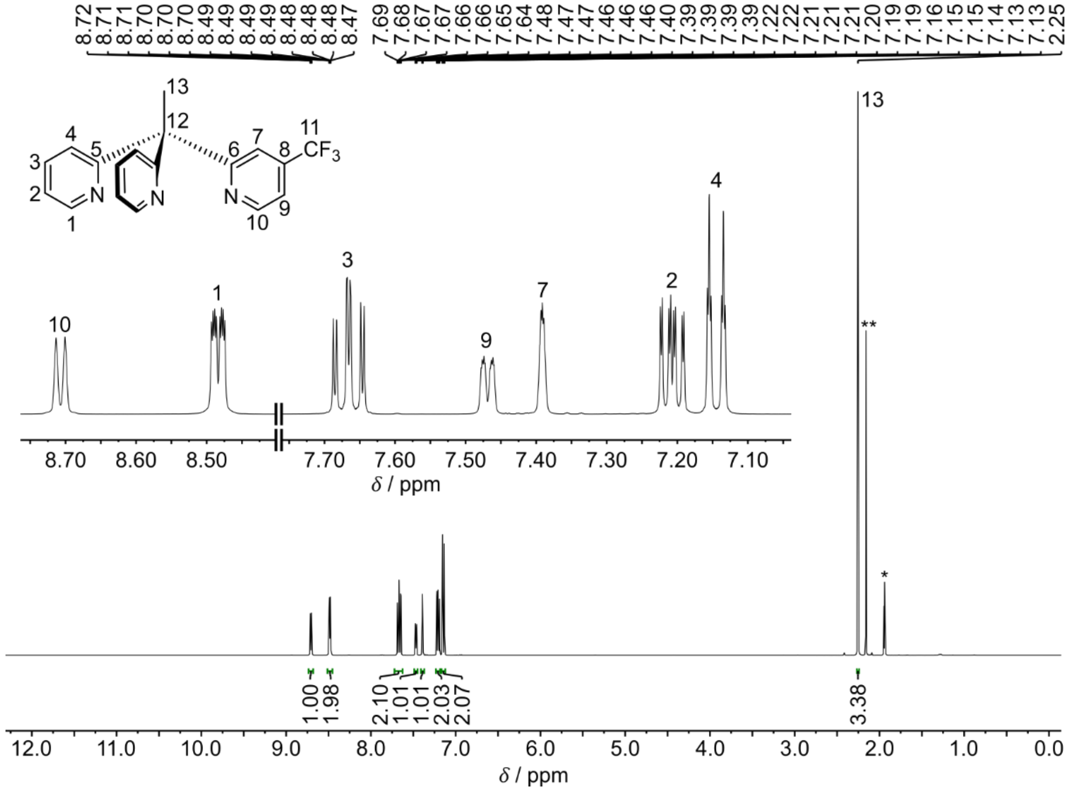

Figure S1. ^{1}H NMR spectrum of tpe^{CF3} in CD_3CN. The inset shows a zoom in the aromatic region. The asterisk * denotes solvent resonance. The double asterisk ** denotes water resonance.

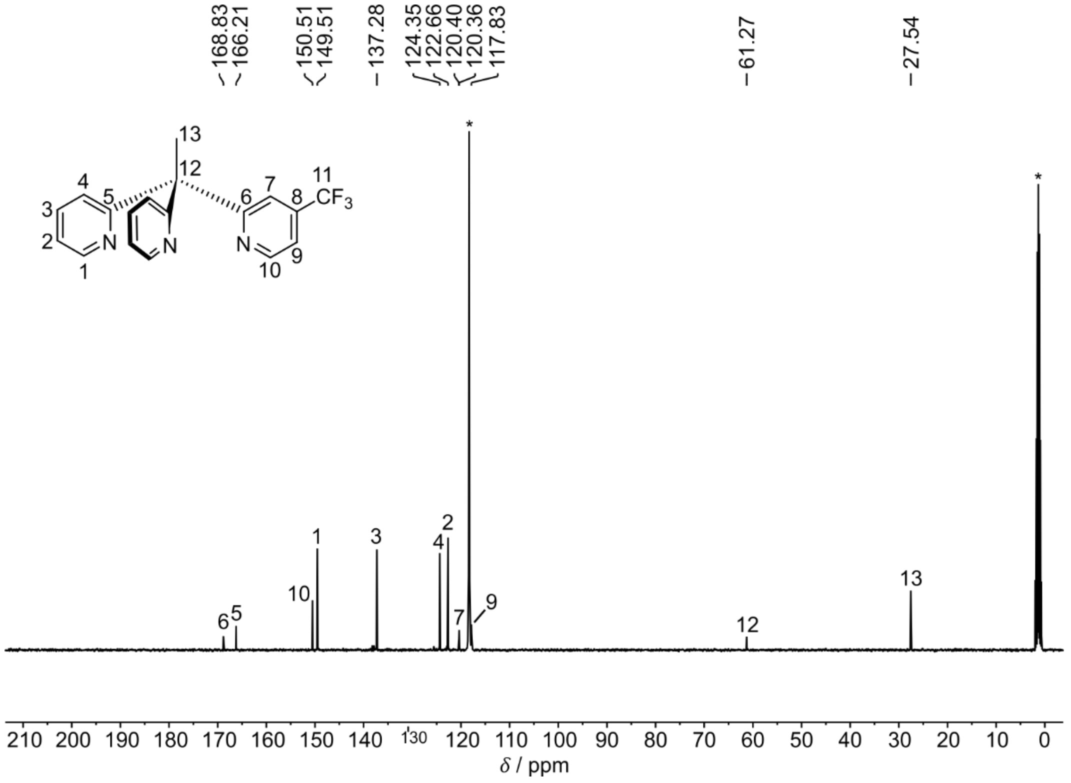

Figure S2. $^{13}C\{^{1}H\}$ NMR spectrum of tpe^{CF3} in CD_3CN. The asterisks * denote solvent resonances.

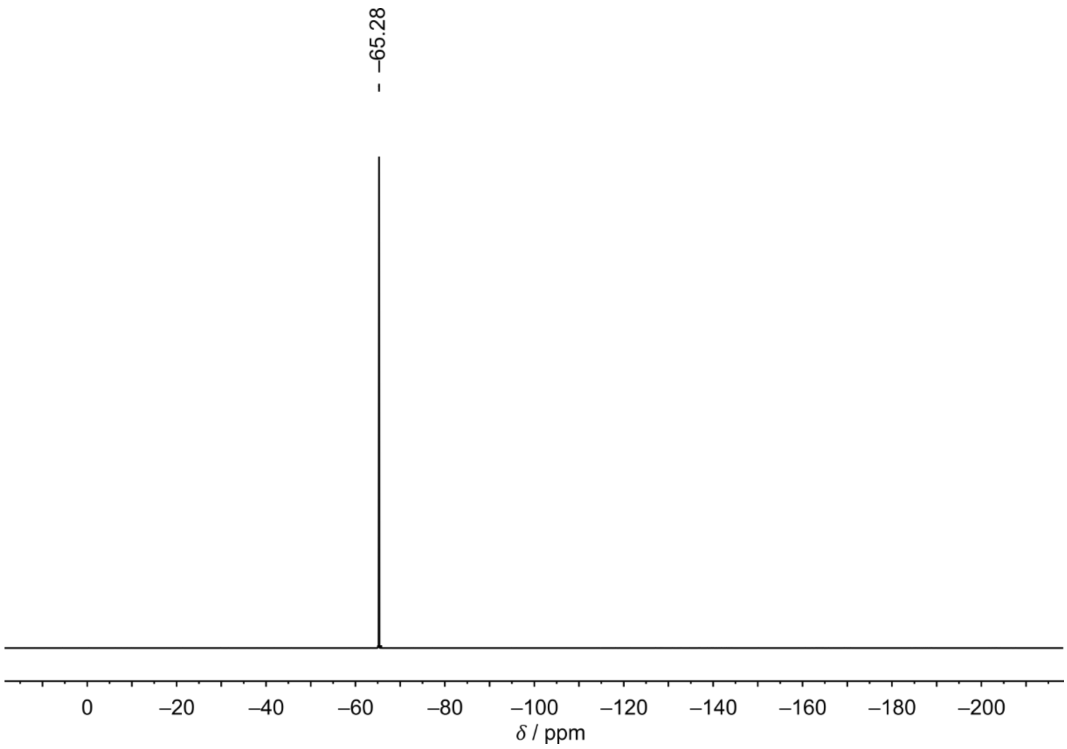

Figure S3. ^{19}F NMR spectrum of tpe^{CF3} in CD_3CN.

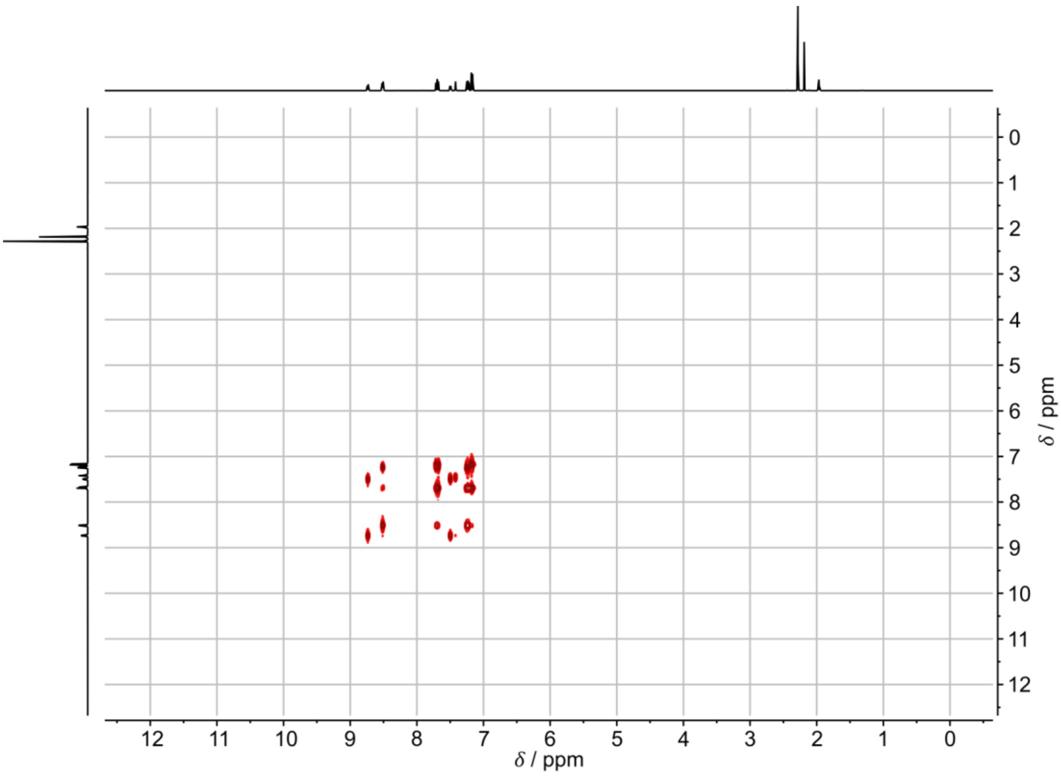

Figure S4. ^{1}H-^{1}H-COSY NMR spectrum of tpe^{CF3} in CD_3CN.

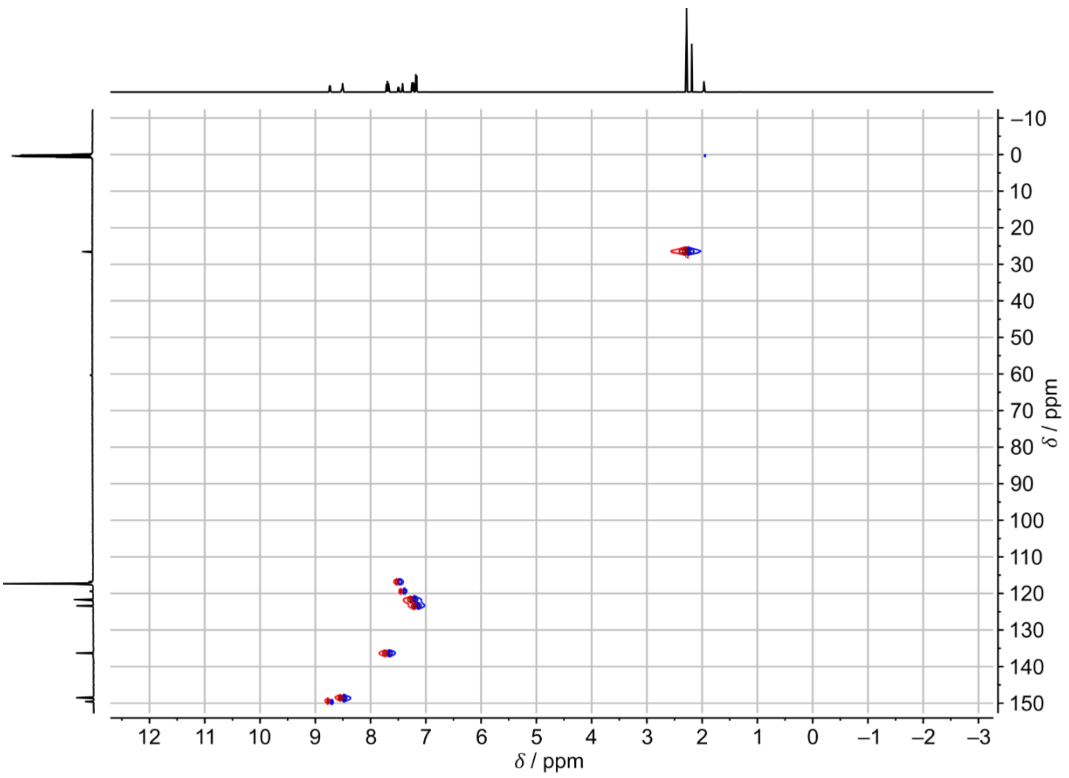

Figure S5. ^{1}H-^{13}C-HSQC NMR spectrum of tpe^{CF3} in CD_3CN.

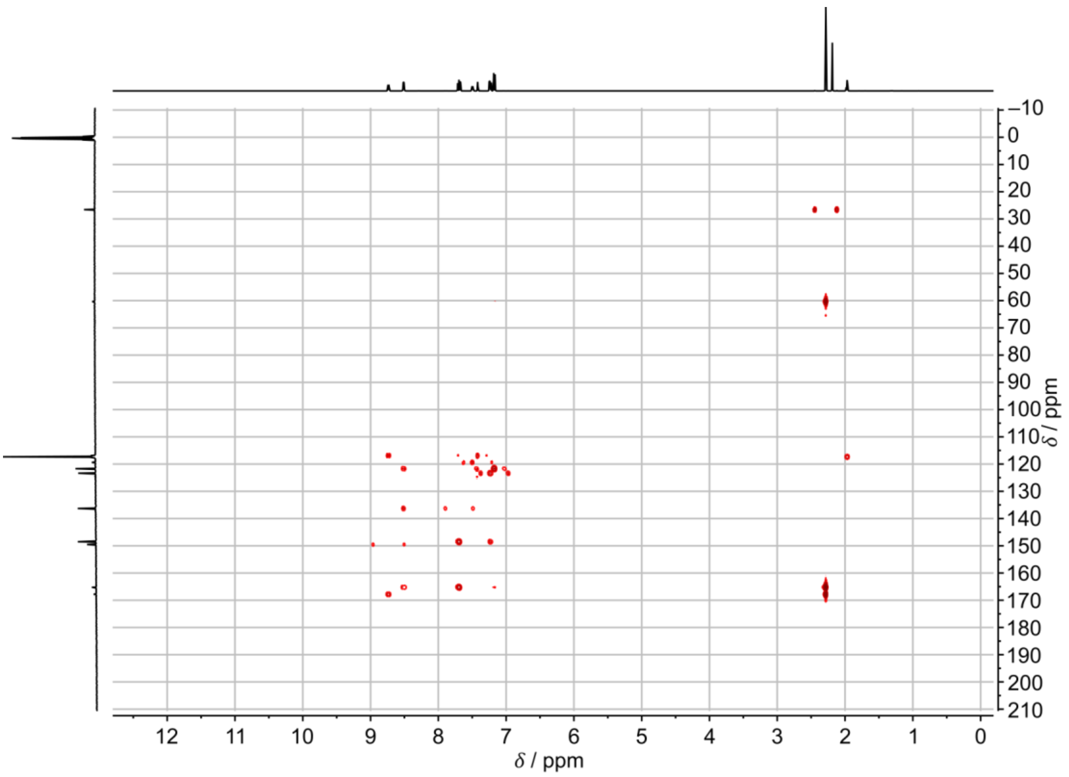

Figure S6. ^{1}H-^{13}C-HMBC NMR spectrum of tpe^{CF3} in CD_3CN.

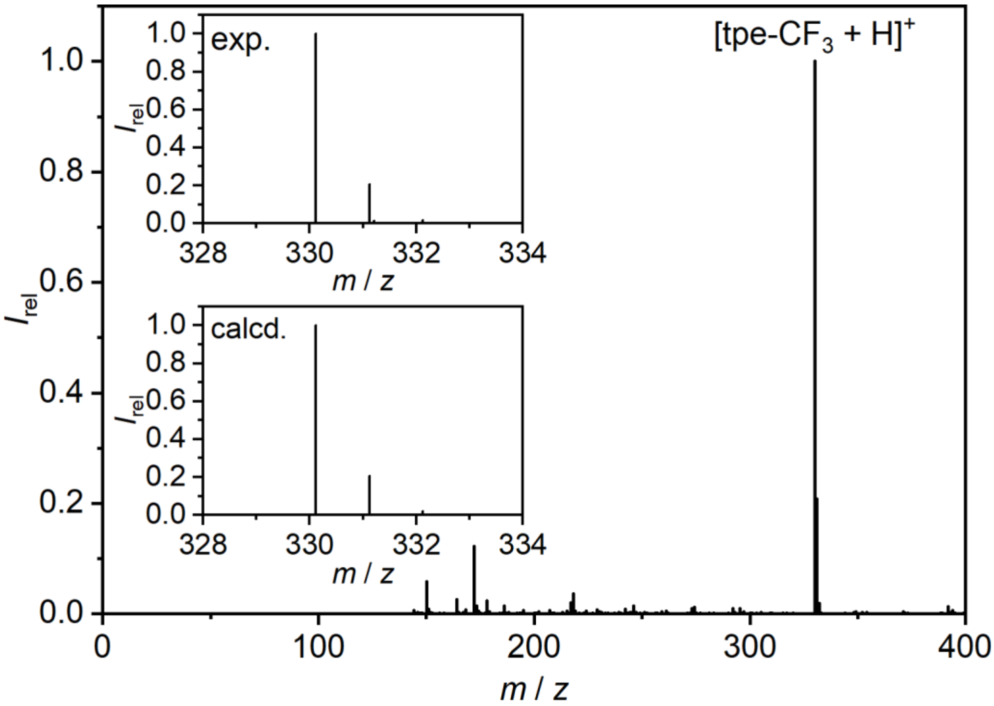

Figure S7. ESI$^+$ mass spectrum of tpe^{CF3} in MeCN. Insets show experimental and calculated ($C_{18}H_{15}F_3N_3$) isotope patterns of the [tpe^{CF3} + H]$^+$ peak, respectively.

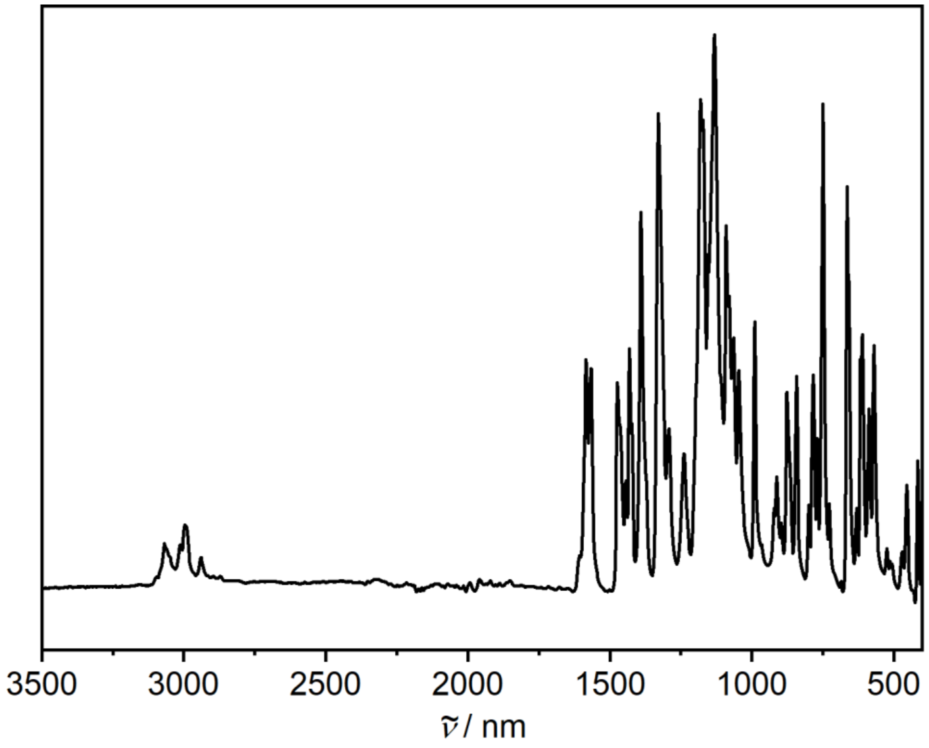

Figure S8. ATR-IR spectrum of tpe^{CF3}.

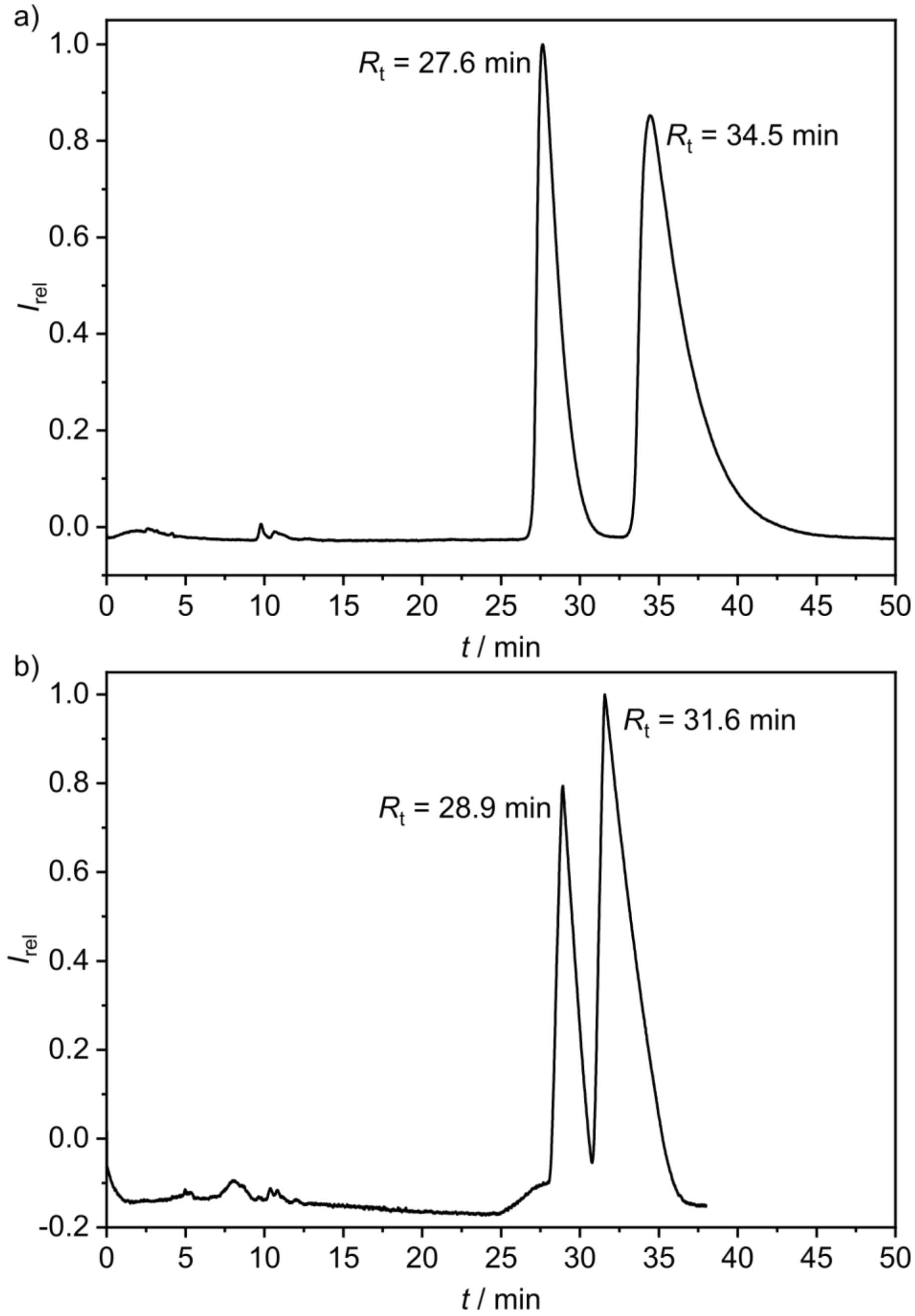

Figure S9. a) Analytical HPLC chromatogram (H_2O (75 mM NaCl)/MeCN (17.5/82.5 *v*/*v*)) of the crude product obtained from the synthesis of $[Cr(tpe^{CF3})_2]^{3+}$. The peaks at R_t = 27.6 min and R_t = 34.5 min integrate as 1:2 and correspond to *trans*-$[Cr(tpe^{CF3})_2]^{3+}$ and *cis*-$[Cr(tpe^{CF3})_2]^{3+}$, respectively. b) Preparative HPLC chromatogram (H_2O (75 mM NaCl)/MeCN (17.5/82.5 *v*/*v*)) of the crude product obtained from the synthesis of $[Cr(tpe^{CF3})_2]^{3+}$. The peaks at R_t = 28.9 min and R_t = 31.6 min correspond to *trans*-$[Cr(tpe^{CF3})_2]^{3+}$ and *cis*-$[Cr(tpe^{CF3})_2]^{3+}$, respectively.

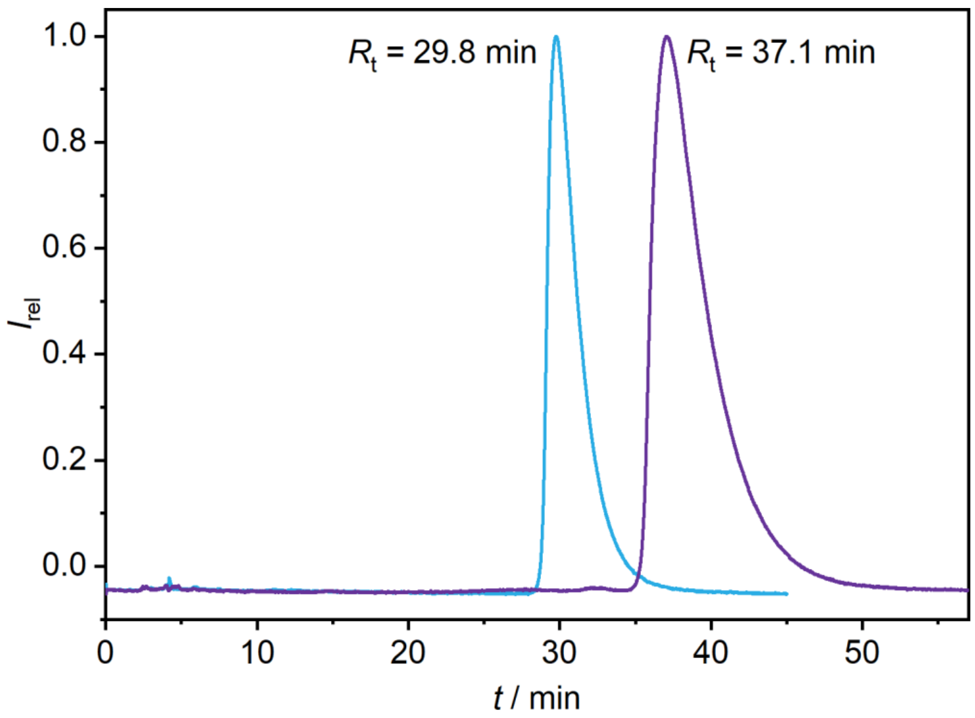

Figure S10. Analytical HPLC chromatograms (H_2O (75 mM NaCl)/MeCN (17.5/82.5 *v*/*v*)) of *trans*-$[Cr(tpe^{CF3})_2]^{3+}$ (blue; R_t = 29.0 min) and *cis*-$[Cr(tpe^{CF3})_2]^{3+}$ (purple; R_t = 37.1 min).

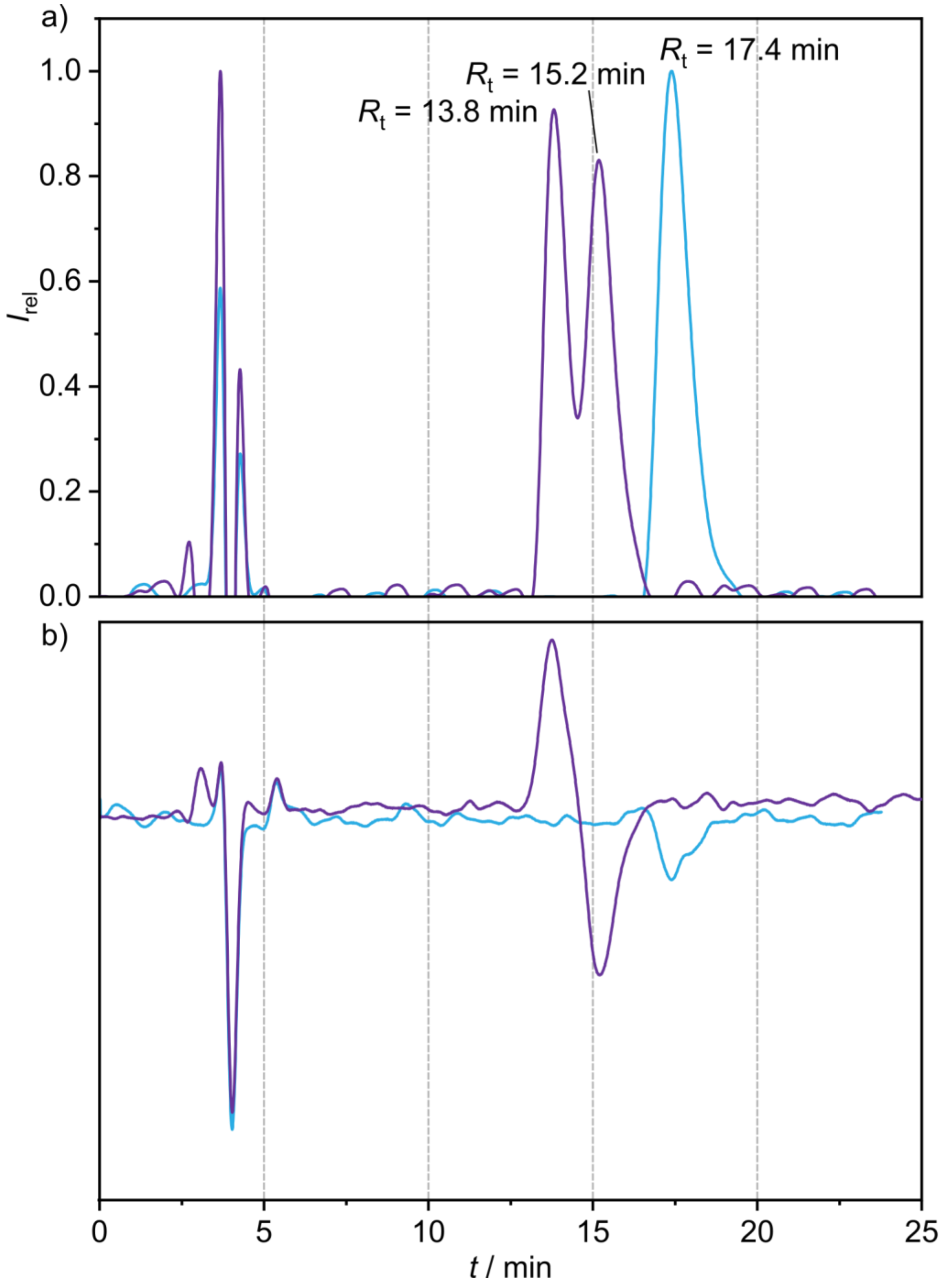

Figure S11. HPLC chromatograms (MeOH (40 mM [n-Bu_4N]Cl)/CH_2Cl_2 (40 mM [n-Bu_4N]Cl; 10/90 v/v); 0.1 % formic acid) of *trans*-[Cr(tpe^{CF3})$_2$]$^{3+}$ (blue; R_t = 17.4 min) and *cis*-[Cr(tpe^{CF3})$_2$]$^{3+}$ (purple; R_t = 13.8/15.2 min) using a chiral column and detection via CD spectroscopy.

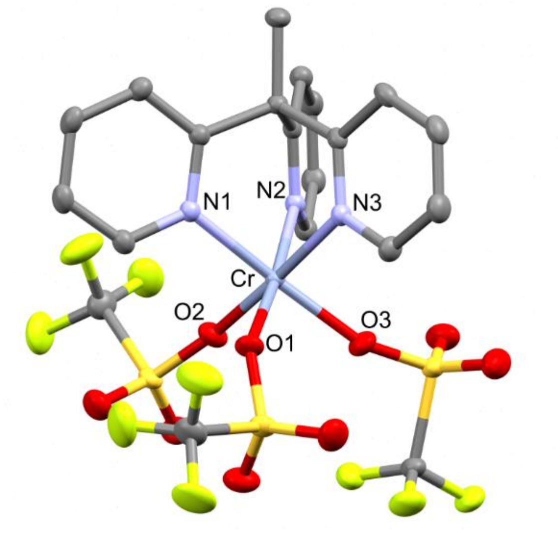

Figure S12. Molecular structure of $Cr(OTf)_3(tpe)$ obtained from single-crystal XRD. Thermal ellipsoids set to 50% probability. Hydrogen atom omitted.

Table S1. Bond lengths [Å] and angles [deg] of $Cr(OTf)_3(tpe)$ obtained from XRD analysis.

bond lengths/Å	
Cr-N1	2.023(18)
Cr-N2	2.031(18)
Cr-N3	2.023(19)
Cr-O1	1.963(17)
Cr-O2	1.959(17)
Cr-O3	1.969(17)
**bond angles/deg**	
N1-Cr-N2	86.91(8)
N1-Cr-N3	85.53(7)
N1-Cr-O1	88.06(9)
N1-Cr-O2	93.76(8)
N1-Cr-O3	175.74(7)
N2-Cr-N3	87.58(7)
N2-Cr-O1	174.94(9)
N2-Cr-O2	87.04(7)
N2-Cr-O3	94.49(8)
N3-Cr-O1	91.59(9)
N3-Cr-O2	174.60(7)
N3-Cr-O3	90.50(8)
O1-Cr-O2	93.74(9)
O1-Cr-O3	90.50(10)
O2-Cr-O3	90.33(8)

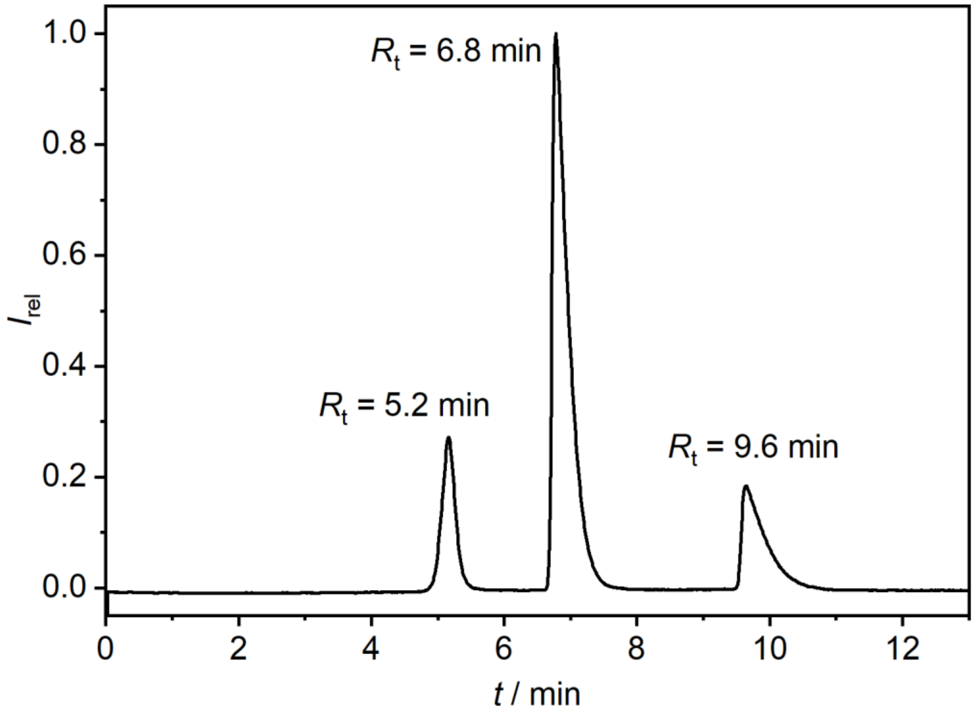

Figure S13. Preparative HPLC chromatogram (H_2O (300 mM NaCl)/MeCN (40/60 *v*/*v*)) of the crude product obtained from the synthesis of $[Cr(tpe)(tpe^{CF3})]^{3+}$ (R_t = 6.8 min). The peaks at R_t = 5.2 min and R_t = 9.6 min stem from homoleptic $[Cr(tpe^{CF3})_2]^{3+}$ (presumably) and $[Cr(tpe)_2]^{3+}$, respectively.

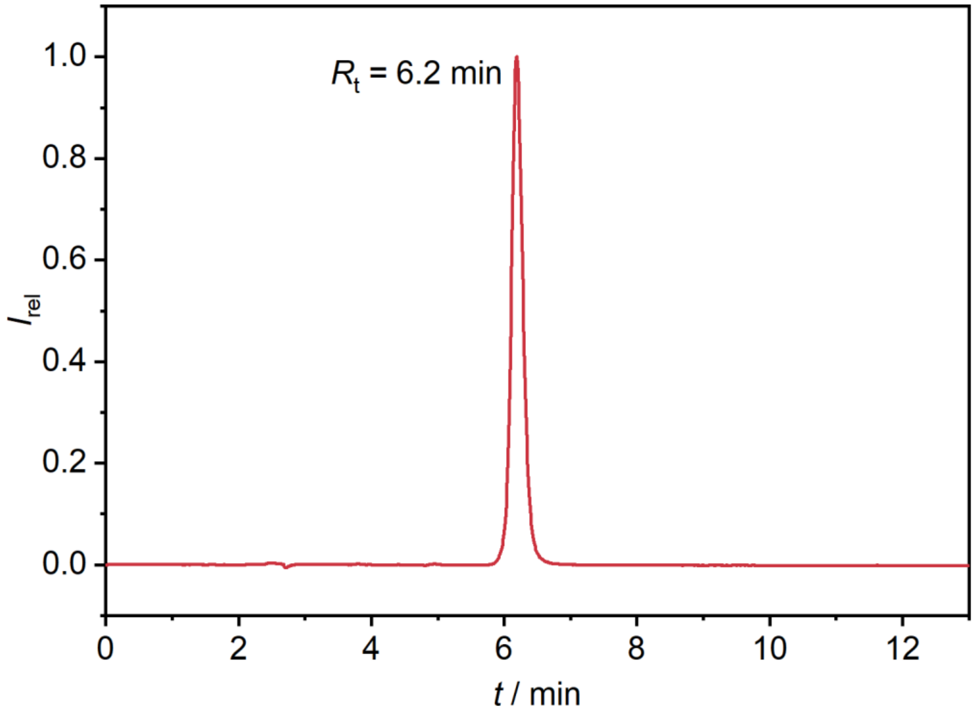

Figure S14. Analytical HPLC chromatogram (H_2O (300 mM NaCl)/MeCN (40/60 *v*/*v*)) of $[Cr(tpe)(tpe^{CF3})]^{3+}$ (R_t = 6.2 min).

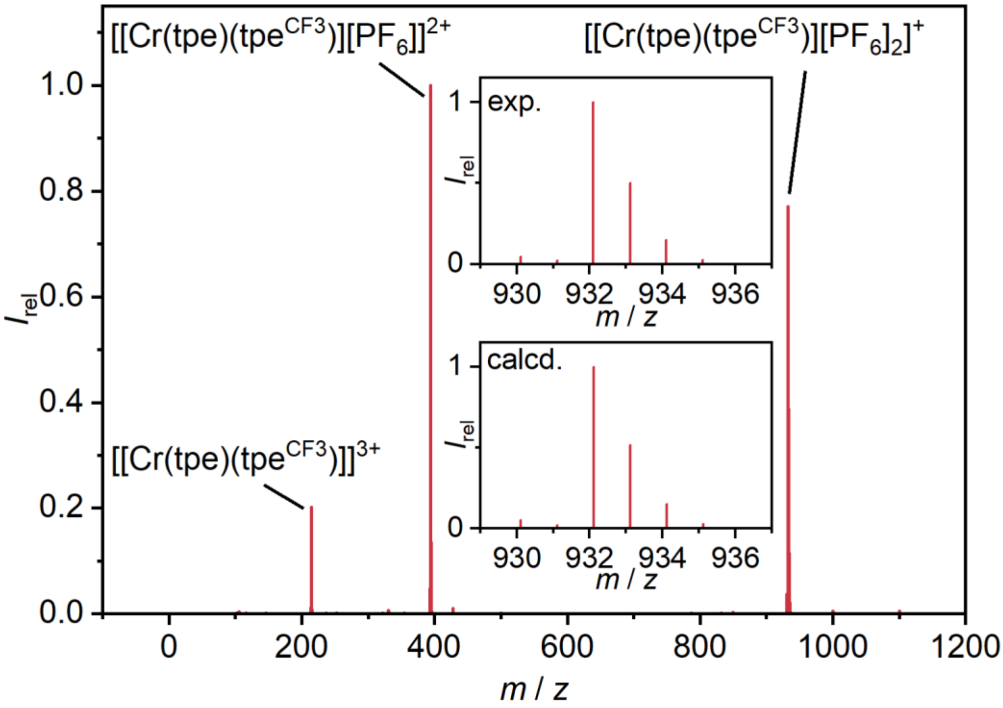

Figure S15. ESI$^+$ mass spectrum of [Cr(tpe)(tpe^{CF3})][PF$_6$]$_3$ in MeCN. Insets show experimental and calculated isotope patterns of the {[Cr(tpe)(tpe^{CF3})][PF$_6$]$_2$}$^+$ peak, respectively.

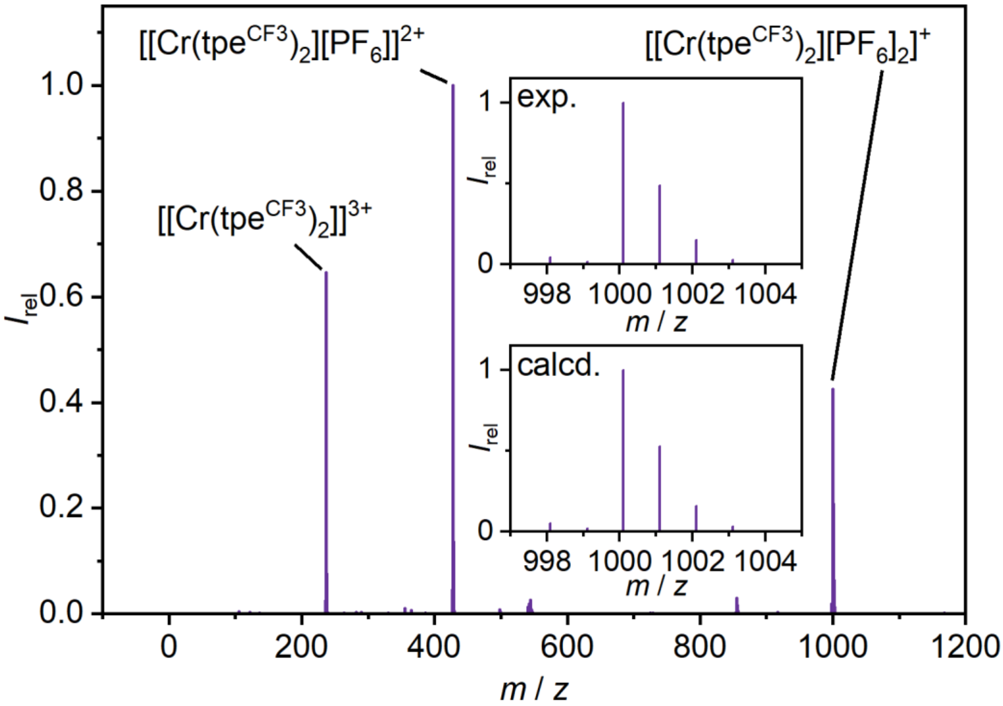

Figure S16. ESI$^+$ mass spectrum of *cis*-[Cr(tpe^{CF3})$_2$][PF$_6$]$_3$ in MeCN. Insets show experimental and calculated isotope patterns of the {[Cr(tpe^{CF3})$_2$][PF$_6$]$_2$}$^+$ peak, respectively.

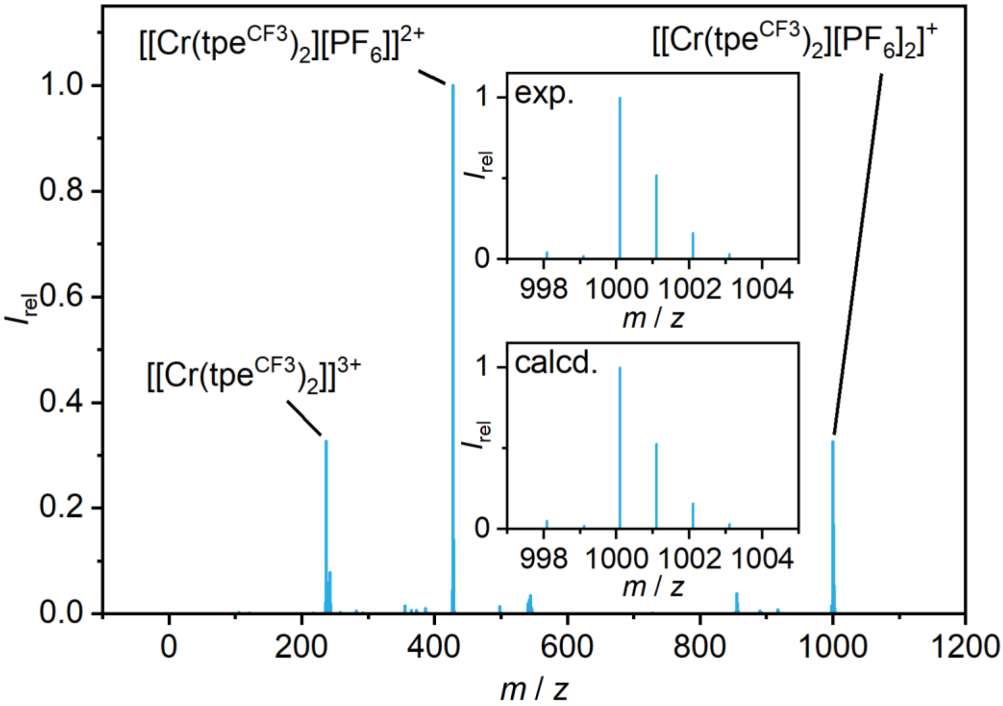

Figure S17. ESI+ mass spectrum of *trans*-[Cr(tpe^{CF3})$_2$][PF$_6$]$_3$ in MeCN. Insets show experimental and calculated isotope patterns of the {[Cr(tpe^{CF3})$_2$][PF$_6$]$_2$}$^+$ peak, respectively.

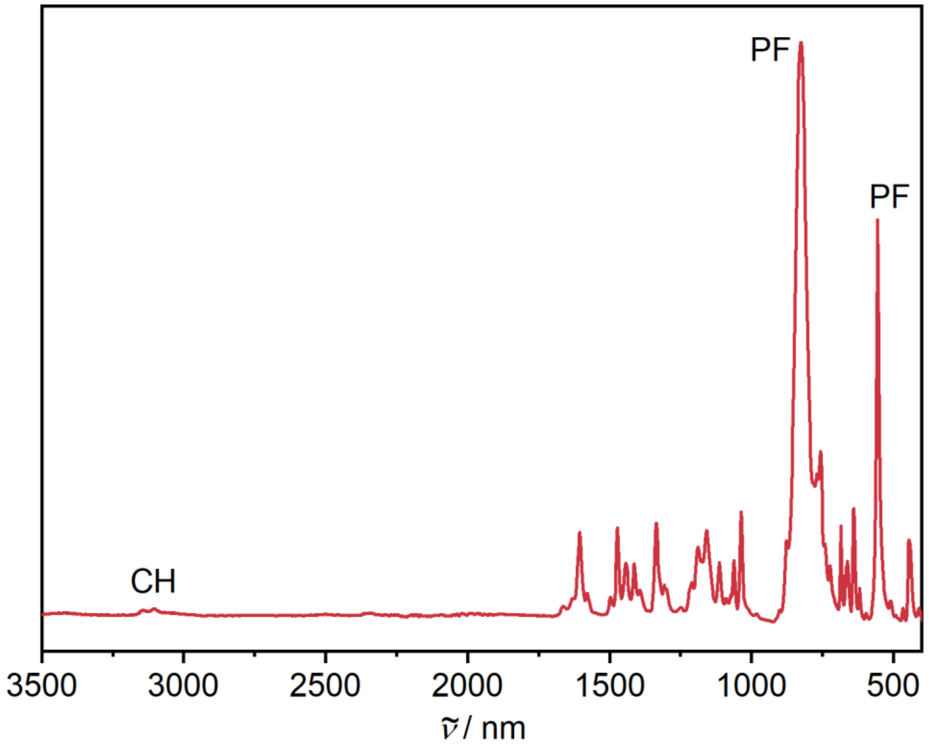

Figure S18. ATR-IR spectrum of [Cr(tpe)(tpe^{CF3})][PF$_6$]$_3$. Some of the characteristic bands have been assigned.[78]

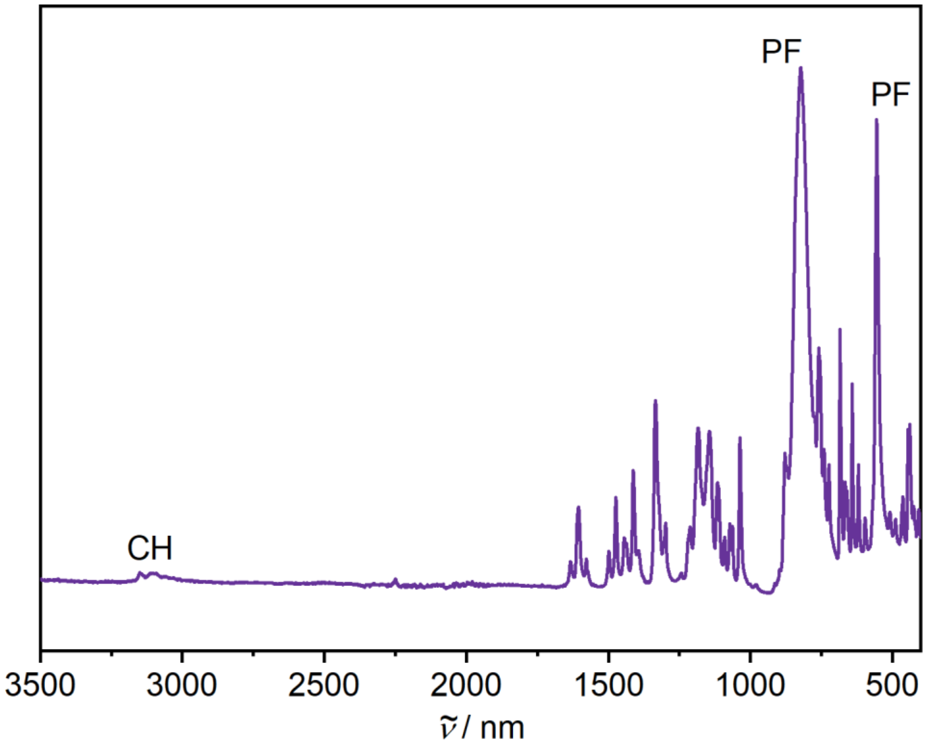

Figure S19. ATR-IR spectrum of *cis*-[Cr(tpe^{CF3})$_2$][PF$_6$]$_3$. Some of the characteristic bands have been assigned.[78]

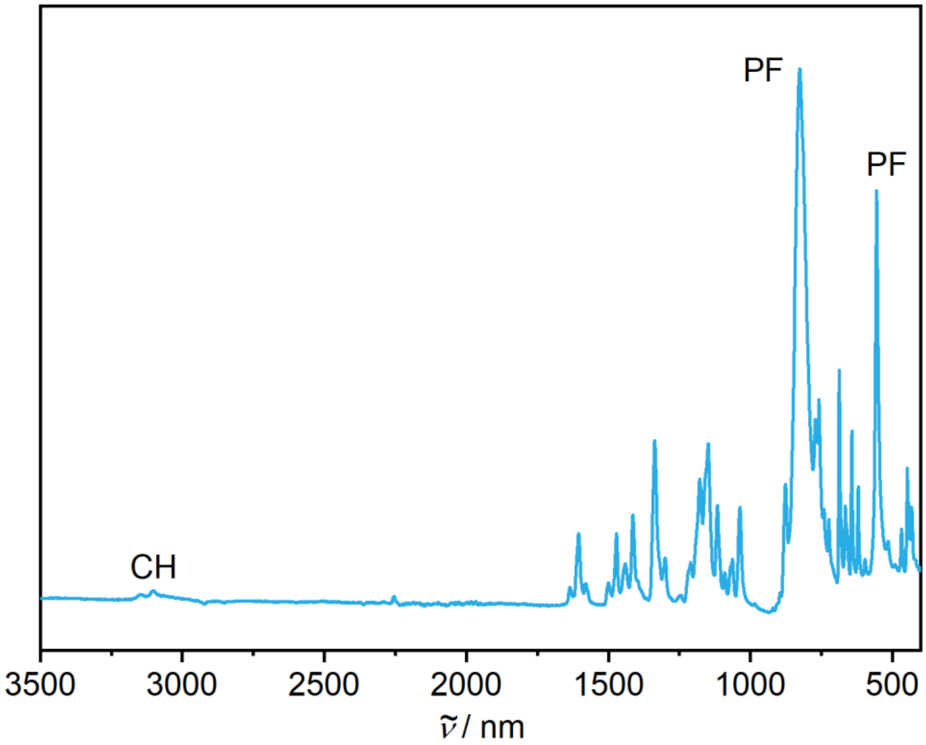

Figure S20. ATR-IR spectrum of *trans*-[Cr(tpe^{CF3})$_2$][PF$_6$]$_3$. Some of the characteristic bands have been assigned.[78]

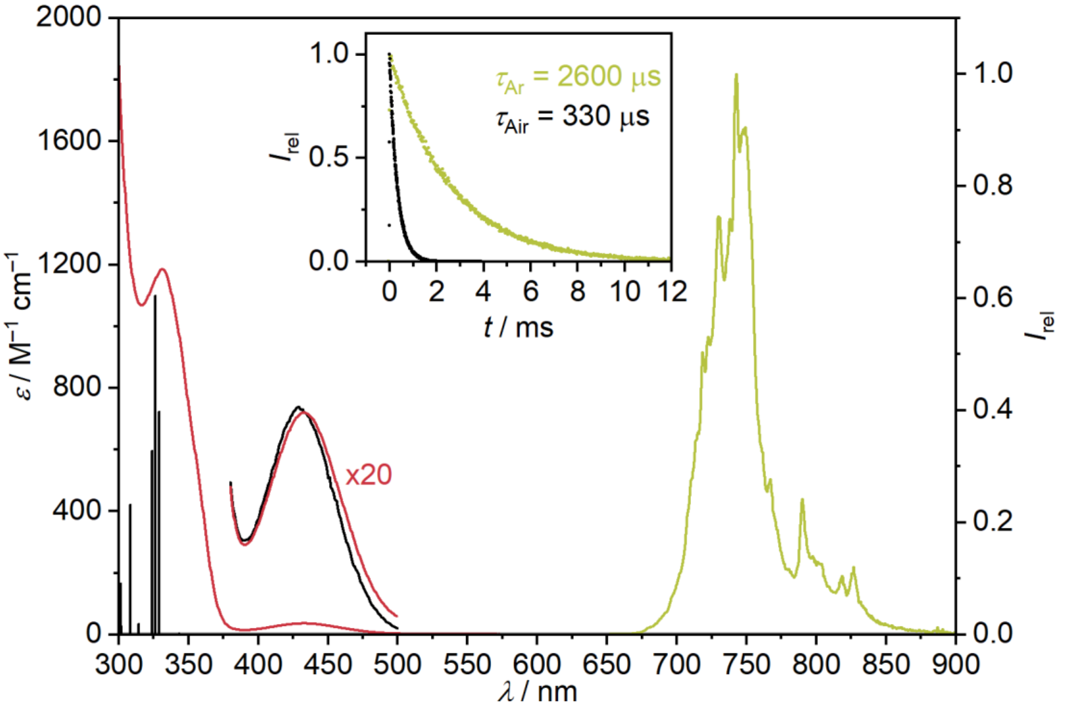

Figure S21. UV/vis absorption (red), normalized excitation (λ_{em} = 742 nm, black) and emission spectra (λ_{exc} = 435 nm, green) of [Cr(tpe)(tpe^{CF3})][PF_6]$_3$ in MeCN. TD-DFT calculated transitions of [Cr(tpe)(tpe^{CF3})]$^{3+}$ shown as vertical bars (black). Inset shows emission decay curves recorded at 742 nm under argon (green) and air (black).

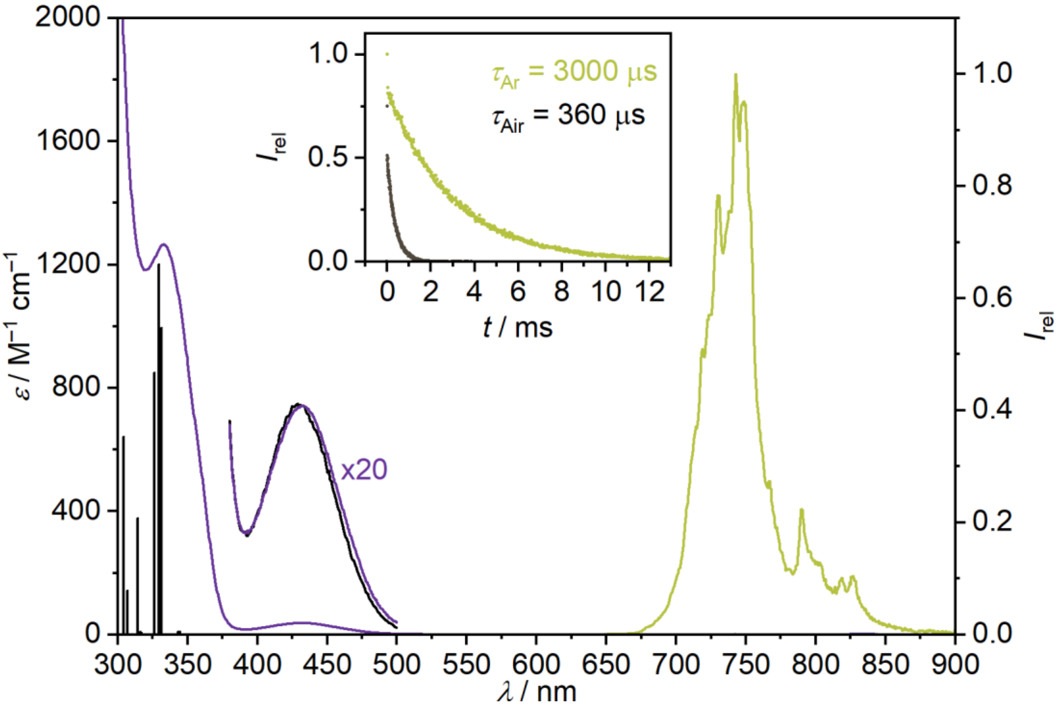

Figure S22. UV/vis absorption (purple), normalized excitation (λ_{em} = 742 nm, black) and emission spectra (λ_{exc} = 435 nm, green) of *cis*-[Cr(tpe^{CF3})$_2$][PF_6]$_3$ in MeCN. TD-DFT calculated transitions of *cis*-[Cr(tpe^{CF3})$_2$]$^{3+}$ shown as vertical bars (black). Inset shows emission decay curves recorded at 742 nm under argon (green) and air (black).

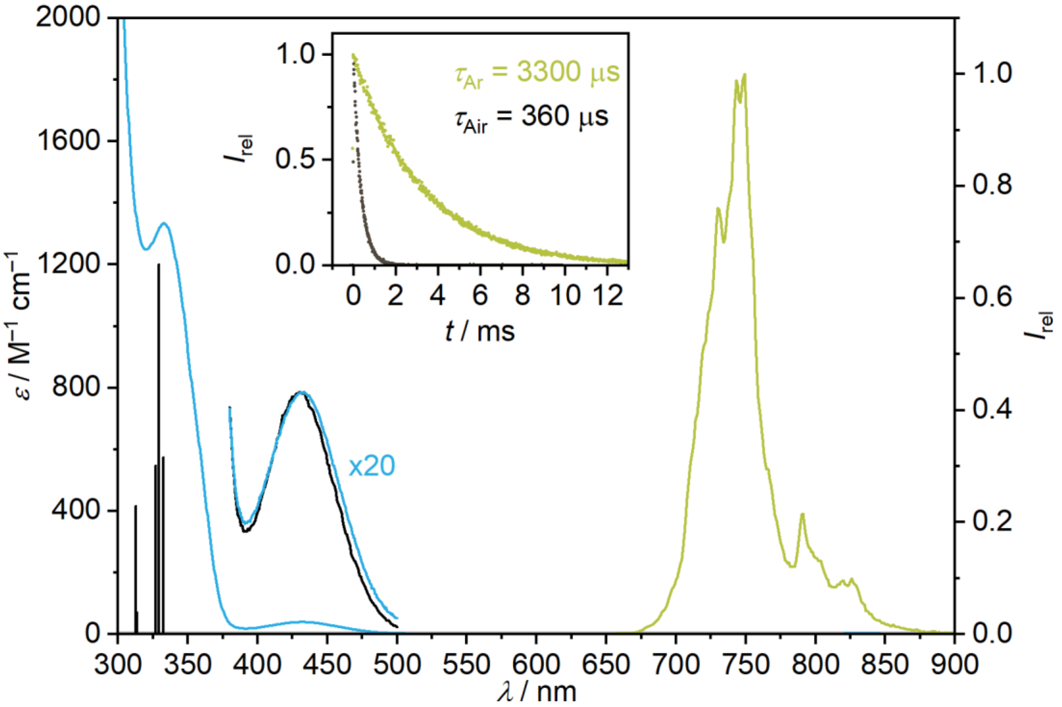

Figure S23. UV/vis absorption (blue), normalized excitation (λ_{em} = 742 nm, black) and emission spectra (λ_{exc} = 435 nm, gree) of *trans*-[Cr(tpe^{CF3})$_2$][PF$_6$]$_3$ in MeCN. TD-DFT calculated transitions of *trans*-[Cr(tpe^{CF3})$_2$]$^{3+}$ shown as vertical bars (black). Inset shows emission decay curves recorded at 742 nm under argon (green) and air (black).

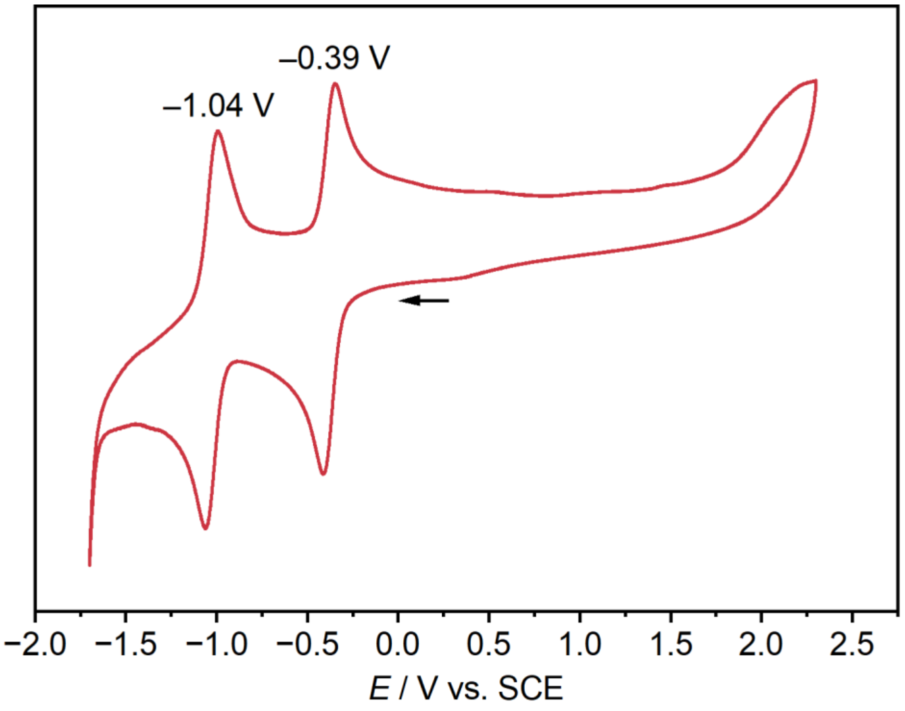

Figure S24. Cyclic voltammogram of 1 mM [Cr(tpe)(tpe^{CF3})][PF$_6$]$_3$ in a 100 mM solution of [*n*-Bu$_4$N][PF$_6$] in MeCN.

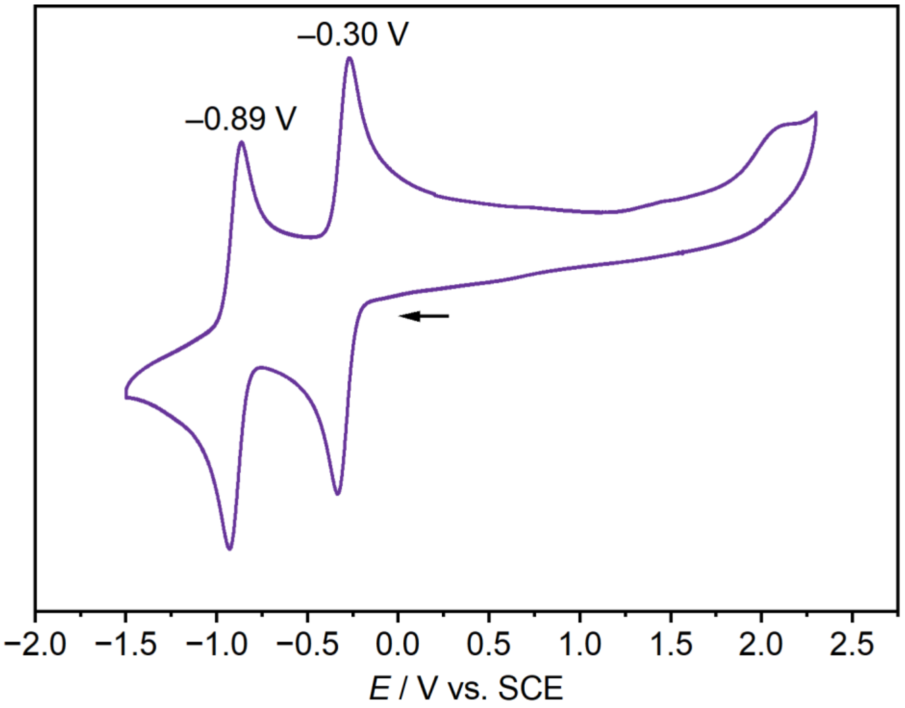

Figure S25. Cyclic voltammogram of 1 mM *cis*-[Cr(tpe^{CF3})$_2$][PF$_6$]$_3$ in a 100 mM solution of [*n*-Bu$_4$N][PF$_6$] in MeCN.

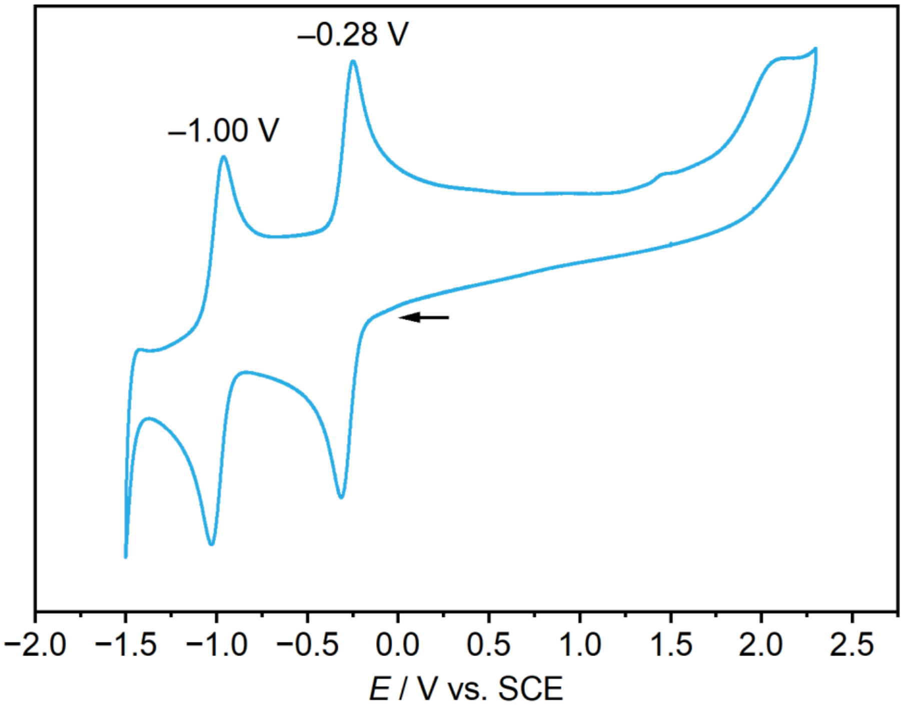

Figure S26. Cyclic voltammogram of 1 mM *trans*-[Cr(tpe^{CF3})$_2$][PF$_6$]$_3$ in a 100 mM solution of [n-Bu$_4$N][PF$_6$] in MeCN.

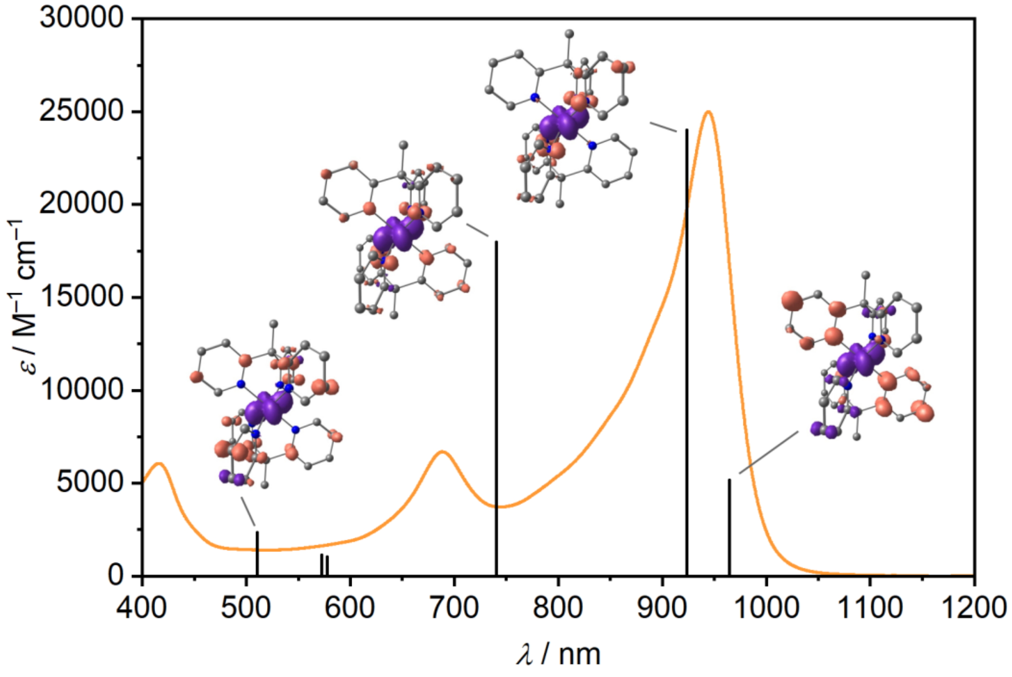

Figure S27. Vis/NIR absorption spectrum of $[Cr(tpe)_2][PF_6]_2$ (orange) prepared by reduction $[Cr(tpe)_2][PF_6]_3$ with cobaltocene in MeCN. TD-DFT calculated transitions of $[Cr(tpe)_2]^{2+}$ shown as vertical bars (black). Selected TD-DFT calculated electronic transitions are depicted. Orange: electron density gain, purple: electron density loss; hydrogen atoms omitted for clarity (CPCM(acetonitrile) ZORA SARC/J RIJCOSX B3LYP D3BJ ZORA-Def2-TZVPP).

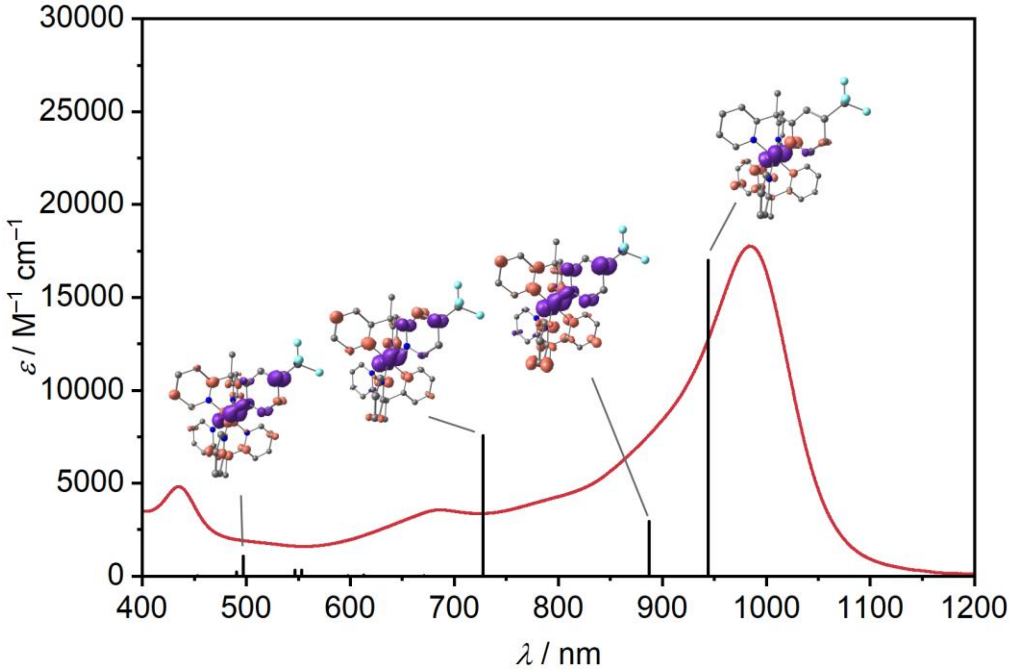

Figure S28. Vis/NIR absorption spectrum of $[Cr(tpe)(tpe^{CF3})][PF_6]_2$ (red) prepared by reduction $[Cr(tpe)(tpe^{CF3})][PF_6]_3$ with cobaltocene in MeCN. TD-DFT calculated transitions of $[Cr(tpe)(tpe^{CF3})]^{2+}$ shown as vertical bars (black). Selected TD-DFT calculated electronic transitions are depicted. Orange/purple: electron density gain/loss; hydrogen atoms omitted for clarity (CPCM(acetonitrile) ZORA SARC/J RIJCOSX B3LYP D3BJ ZORA-Def2-TZVPP).

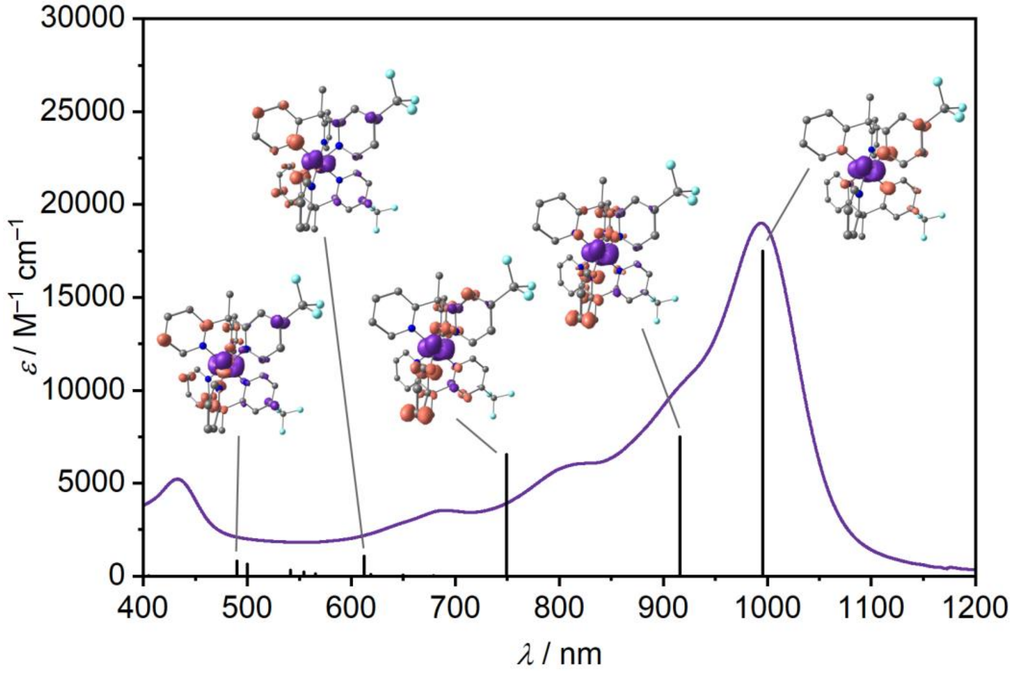

Figure S29. Vis/NIR absorption spectrum of *cis*-$[Cr(tpe^{CF3})_2][PF_6]_2$ (purple) prepared by reduction *cis*-$[Cr(tpe^{CF3})_2][PF_6]_3$ with cobaltocene in MeCN. TD-DFT calculated transitions of *cis*-$[Cr(tpe^{CF3})_2]^{2+}$ shown as vertical bars (black). Selected TD-DFT calculated electronic transitions are depicted. Orange/purple: electron density gain/loss; hydrogen atoms omitted for clarity (CPCM(acetonitrile) ZORA SARC/J RIJCOSX B3LYP D3BJ ZORA-Def2-TZVPP).

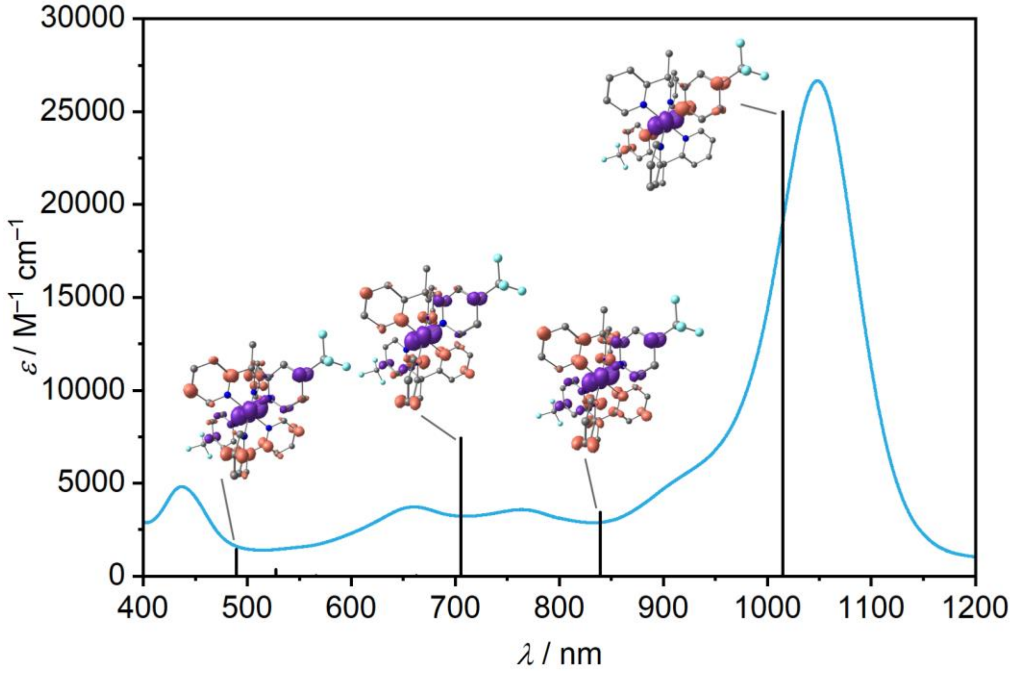

Figure S30. Vis/NIR absorption spectrum of *trans*-$[Cr(tpe^{CF3})_2][PF_6]_2$ (blue) prepared by reduction *trans*-$[Cr(tpe^{CF3})_2][PF_6]_3$ with cobaltocene in MeCN. TD-DFT calculated transitions of *trans*-$[Cr(tpe^{CF3})_2]^{2+}$ shown as vertical bars (black). Selected TD-DFT calculated electronic transitions are depicted. Orange/purple: electron density gain/loss; hydrogen atoms omitted for clarity (CPCM(acetonitrile) ZORA SARC/J RIJCOSX B3LYP D3BJ ZORA-Def2-TZVPP).

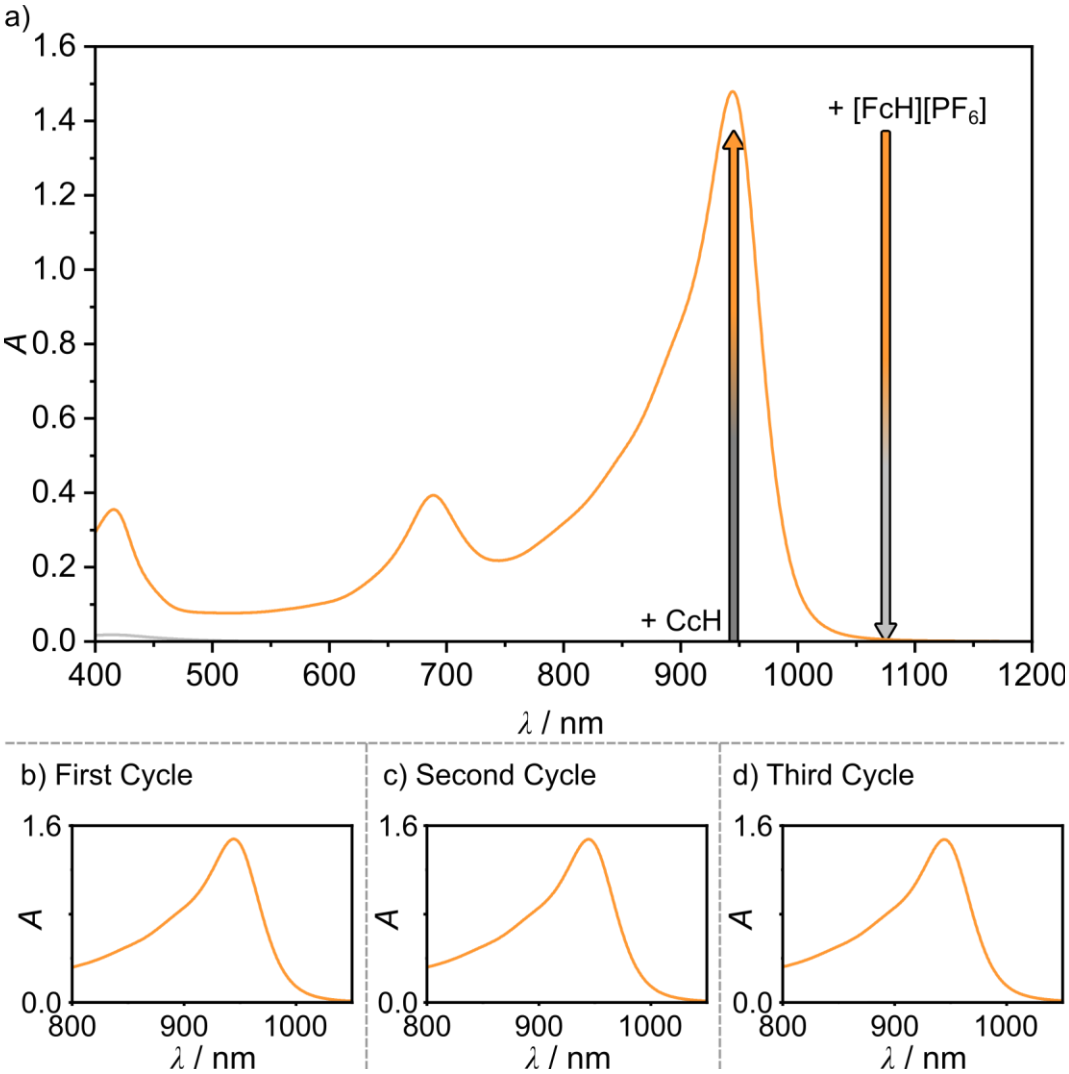

Figure S31. a) UV/vis/NIR absorption spectra of $[Cr(tpe)_2]^{3+}$ (dark gray), $[Cr(tpe)_2]^{2+}$ obtained by reduction of $[Cr(tpe)_2]^{3+}$ with cobaltocene (CcH; orange) and $[Cr(tpe)_2]^{3+}$ after reoxidation of $[Cr(tpe)_2]^{2+}$ with ferrocenium hexafluorophosphate ([FcH][PF_6]; light gray) in MeCN. b)–d) Excerpts of NIR absorption spectra from three consecutive reduction/oxidation cycles. The $[Cr(tpe)_2]^{3+}$ complex does not absorb in the NIR spectral region.

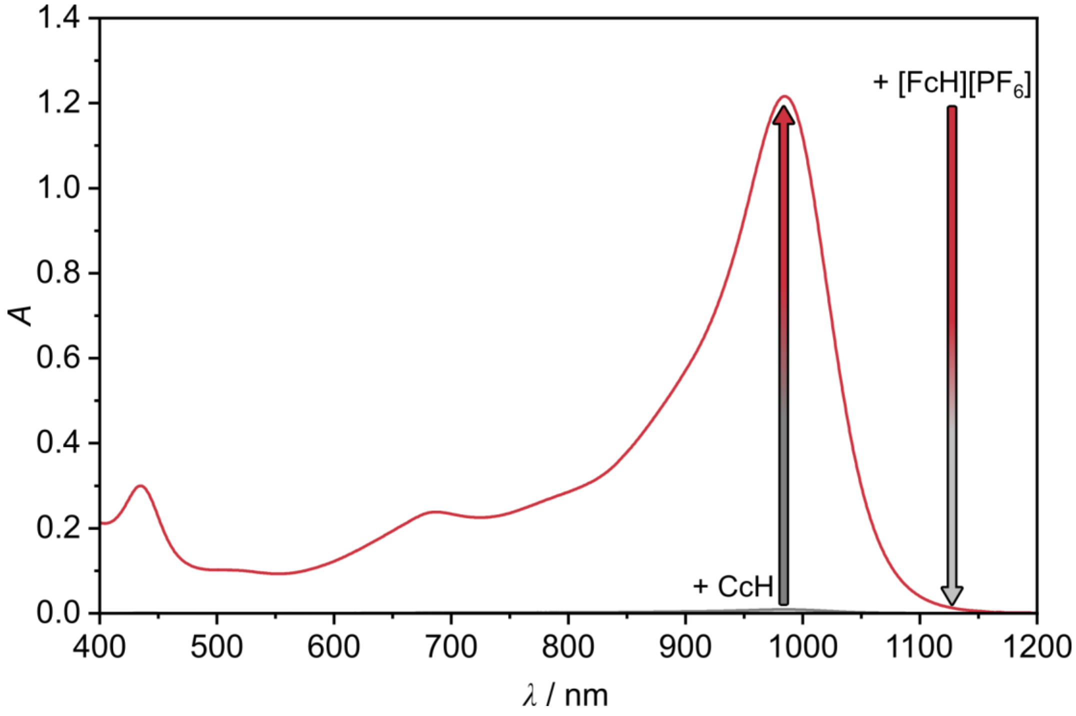

Figure S32. UV/vis/NIR absorption spectra of $[Cr(tpe)(tpe^{CF3})]^{3+}$ (dark gray), $[Cr(tpe)(tpe^{CF3})]^{2+}$ obtained by reduction of $[Cr(tpe)(tpe^{CF3})]^{3+}$ with cobaltocene (CcH; red) and $[Cr(tpe)(tpe^{CF3})]^{3+}$ after reoxidation with ferrocenium hexafluorophosphate ([FcH][PF_6]; light gray) in MeCN.

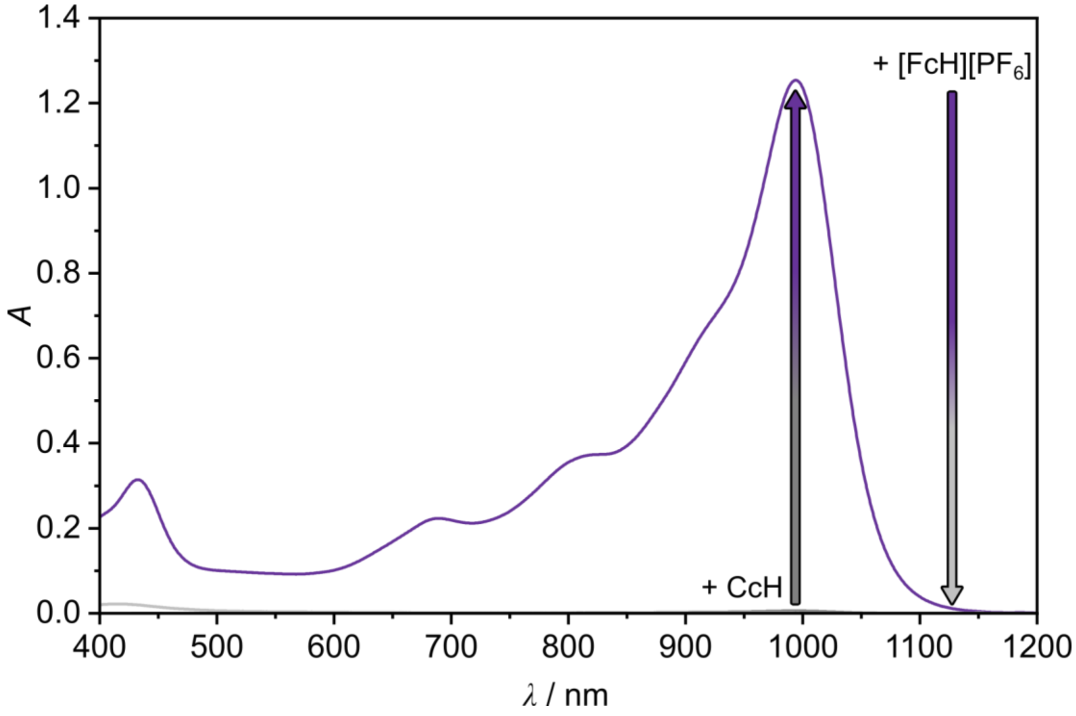

Figure S33. UV/vis/NIR absorption spectra of *cis*-$[Cr(tpe^{CF3})_2]^{3+}$ (dark gray), *cis*-$[Cr(tpe^{CF3})_2]^{2+}$ obtained by reduction of *cis*-$[Cr(tpe^{CF3})_2]^{3+}$ with cobaltocene (CcH; purple) and *cis*-$[Cr(tpe^{CF3})_2]^{3+}$ after reoxidation with ferrocenium hexafluorophosphate ([FcH][PF_6]; light gray) in MeCN.

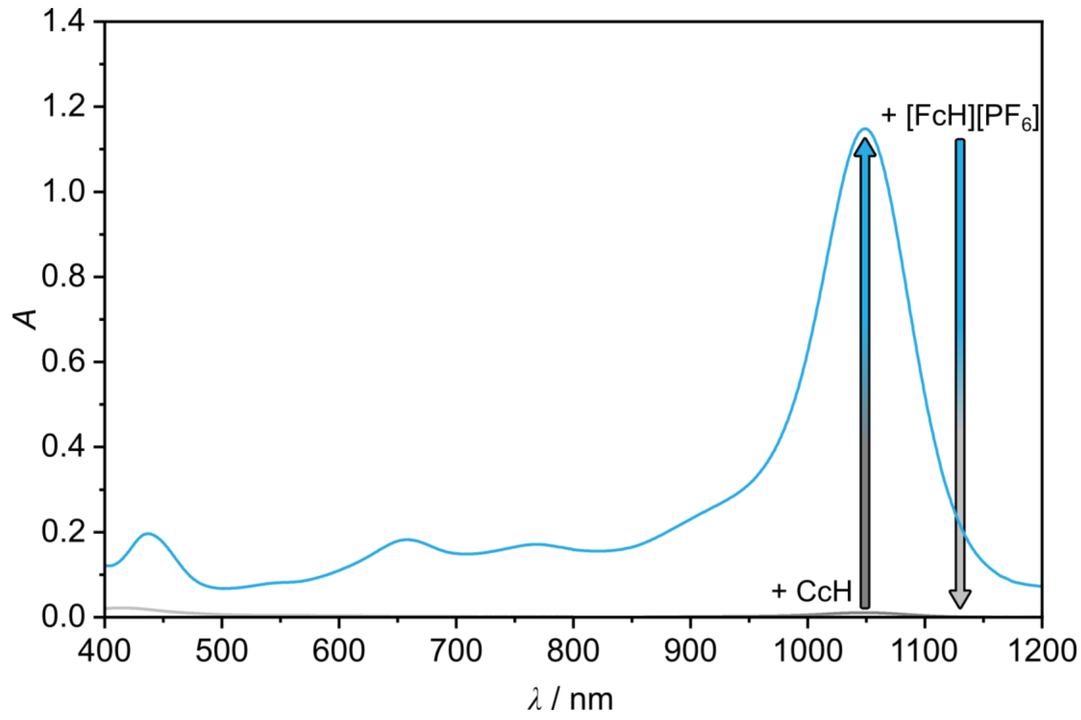

Figure S34. UV/vis/NIR absorption spectra of *trans*-[Cr(tpe^{CF3})$_2$]$^{3+}$ (dark gray), *trans*-[Cr(tpe^{CF3})$_2$]$^{2+}$ obtained by reduction of *trans*-[Cr(tpe^{CF3})$_2$]$^{3+}$ with cobaltocene (CcH; blue) and *trans*-[Cr(tpe^{CF3})$_2$]$^{3+}$ after reoxidation with ferrocenium hexafluorophosphate ([FcH][PF$_6$]; light gray) in MeCN.

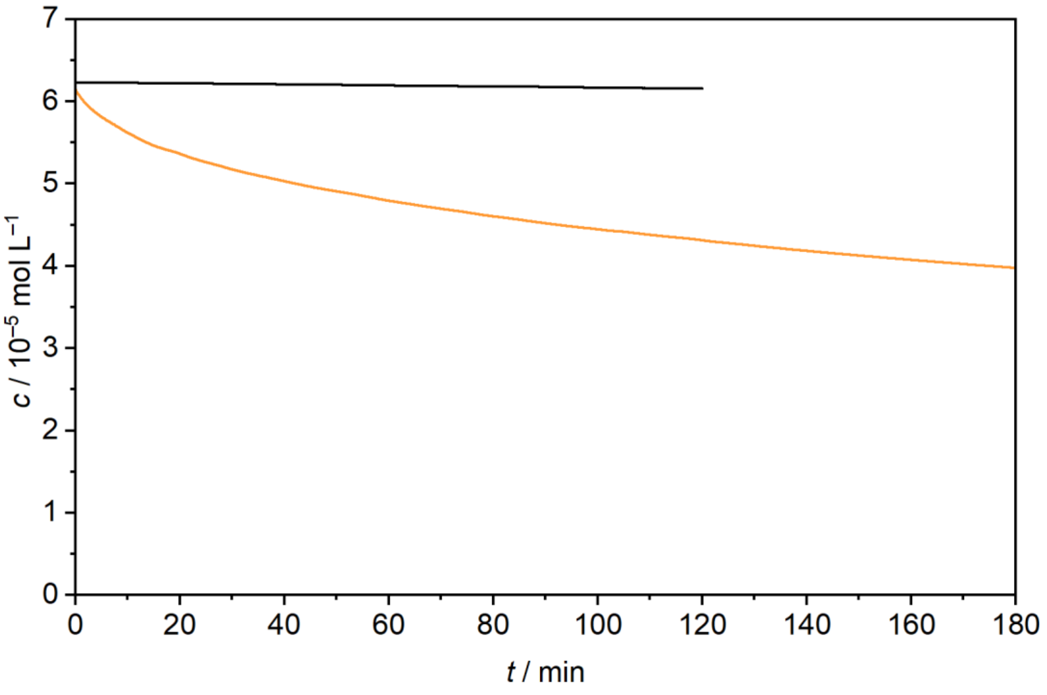

Figure S35. Decrease of the concentration of $[Cr(tpe)_2]^{2+}$ (obtained by reduction of $[Cr(tpe)_2]^{2+}$ with cobaltocene) in air-free (black) and air-saturated (orange) MeCN over time. The concentration was determined using the absorbance at λ_{abs} = 944 nm.

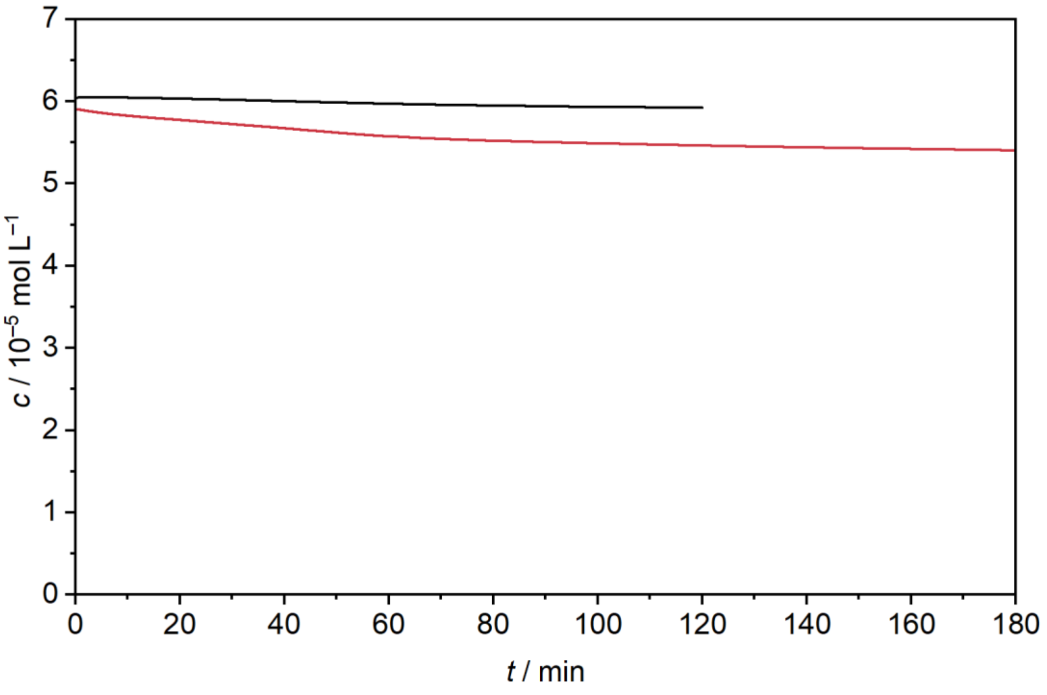

Figure S36. Decrease of the concentration of $[Cr(tpe)(tpe^{CF3})]^{2+}$ (obtained by reduction of $[Cr(tpe)(tpe^{CF3})]^{2+}$ with cobaltocene) in air-free (black) air-saturated (red) MeCN over time. The concentration was determined using the absorbance at λ_{abs} = 985 nm.

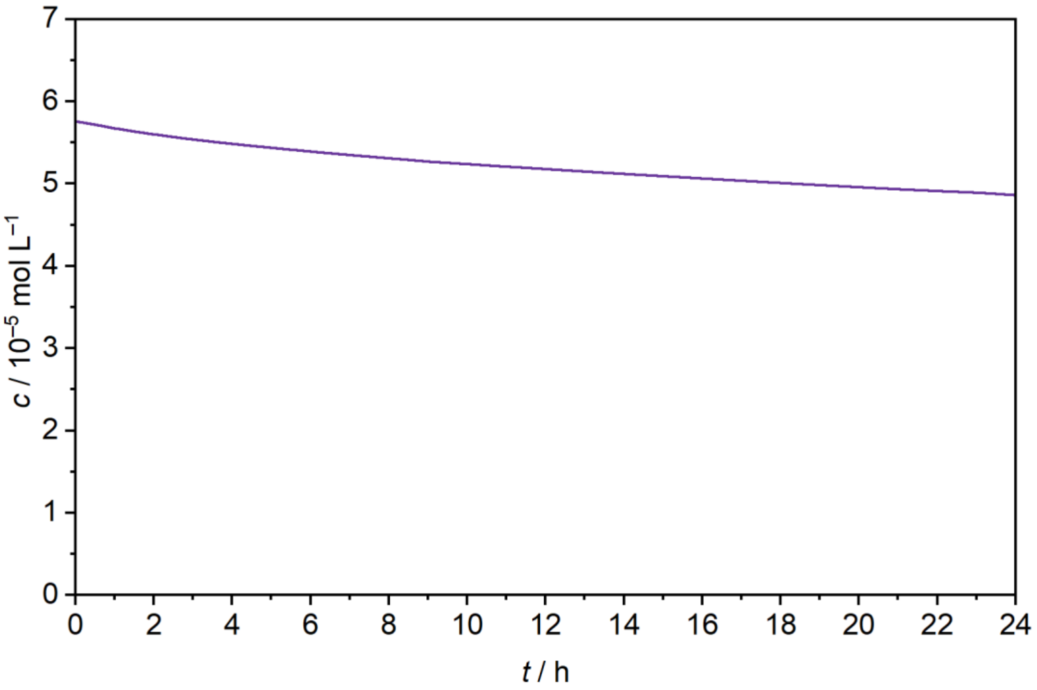

Figure S37. Decrease of the concentration of *cis*-$[Cr(tpe^{CF3})_2]^{2+}$ (obtained by reduction of *cis*-$[Cr(tpe^{CF3})_2]^{2+}$ with cobaltocene) in air-saturated MeCN (purple) over time. The concentration was determined using the absorbance at λ_{abs} = 995 nm. Note the different time scale with respect to Figures S35 and S36.

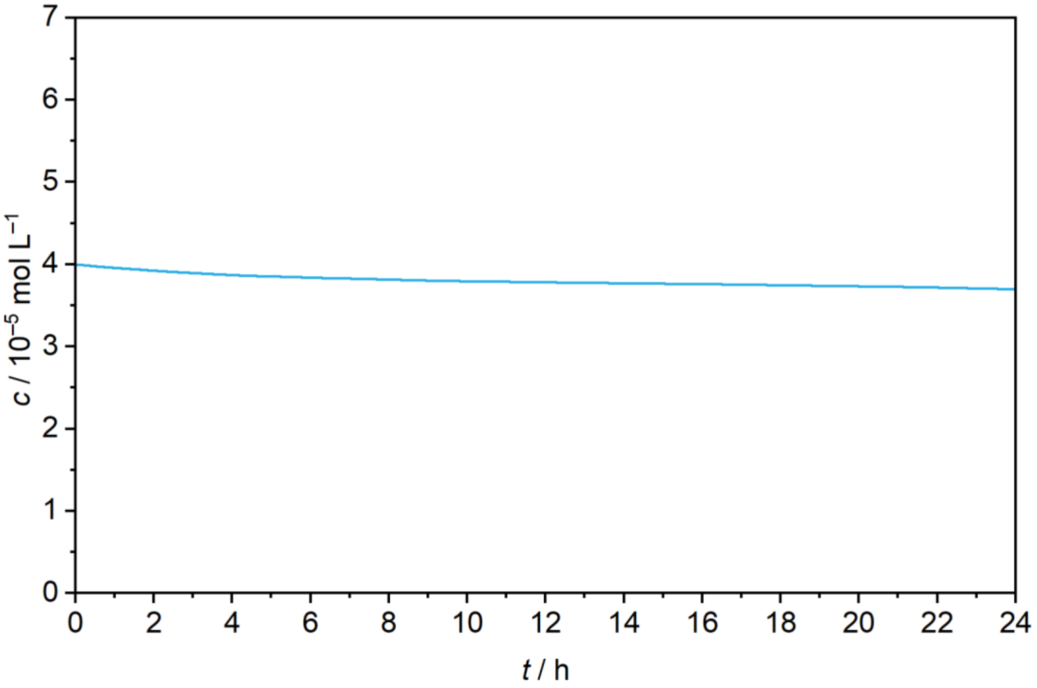

Figure S38. Decrease of the concentration of *trans*-$[Cr(tpe^{CF3})_2]^{2+}$ (obtained by reduction of *trans*-$[Cr(tpe^{CF3})_2]^{2+}$ with cobaltocene) in air-saturated MeCN (blue) over time. The concentration was determined using the absorbance at λ_{abs} = 1048 nm. Note the different time scale with respect to Figures S35 and S36.

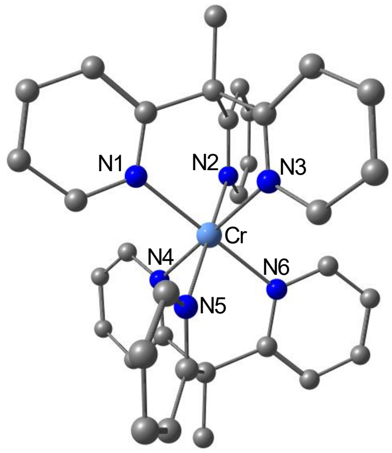

Figure S39. DFT-optimized structure of $^4[Cr(tpe)_2]^{3+}$ with atom labels for Table S2.

Table S2. Bond lengths [Å] and angles [deg] of $[Cr(tpe)_2]^{3+}$ in the DFT-optimized quartet and doublet states $^4[Cr]^{3+}$ / $^2[Cr]^{3+}$ and of $[Cr(tpe)_2]^{2+}$ in the DFT-optimized triplet and quintet states $^3[Cr]^{2+}$ / $^5[Cr]^{2+}$. Gibbs free energies G [H] and relative energies $E_{rel} = G(^2[Cr]^{3+}) - G(^4[Cr]^{3+})$ and $E_{rel} = G(^3[Cr]^{2+}) - G(^5[Cr]^{2+})$ [eV]. Mulliken spin density at Cr.

	$^4[Cr]^{3+}$	$^2[Cr]^{3+}$	$^3[Cr]^{2+}$	$^5[Cr]^{2+}$
**Cr-N1**	2.056	2.050	2.030	2.117
**Cr-N2**	2.055	2.048	2.067	2.302
**Cr-N3**	2.055	2.049	2.046	2.086
**Cr-N4**	2.055	2.049	2.046	2.110
**Cr-N5**	2.055	2.048	2.067	2.333
**Cr-N6**	2.056	2.050	2.030	2.093
**N1-Cr-N2**	86.81	86.79	86.92	81.14
**N1-Cr-N3**	86.78	86.77	86.77	84.22
**N1-Cr-N4**	93.22	93.23	93.23	95.79
**N1-Cr-N5**	93.19	93.21	93.08	92.57
**N1-Cr-N6**	180.00	180.00	180.00	179.88
**N2-Cr-N3**	86.82	86.96	86.86	86.82
**N2-Cr-N4**	93.18	93.04	93.13	94.61
**N2-Cr-N5**	180.00	180.00	180.00	171.53
**N2-Cr-N6**	93.20	93.21	93.07	98.76
**N3-Cr-N4**	180.00	180.00	179.99	178.56
**N3-Cr-N5**	93.18	93.04	93.14	98.24
**N3-Cr-N6**	93.22	93.23	93.23	95.85
**N4-Cr-N5**	86.82	86.96	86.87	80.32
**N4-Cr-N6**	86.78	86.77	86.77	84.15
**N5-Cr-N6**	86.81	86.79	86.92	87.52
***G* / H**	−2696.801	−2696.764	−2696.939	−2696.933
**E_{rel} / eV**	0	1.01	0	0.16
**Spin density at Cr**	3.22	1.13	2.51	3.96

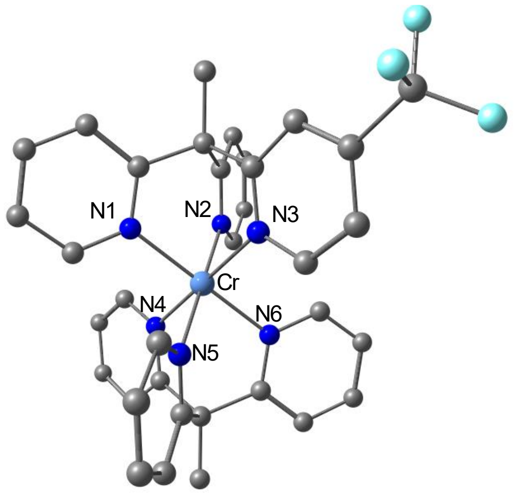

Figure S40. DFT-optimized structure of $^4[Cr(tpe)(tpe^{CF3})]^{3+}$ with atom labels for Table S3.

Table S3. Bond lengths [Å] and angles [deg] of $[Cr(tpe)(tpe^{CF3})]^{3+}$ in the DFT-optimized quartet and doublet states $^4[Cr]^{3+}$ / $^2[Cr]^{3+}$ and of $[Cr(tpe)(tpe^{CF3})]^{2+}$ in the DFT-optimized triplet and quintet states $^3[Cr]^{2+}$ / $^5[Cr]^{2+}$. Gibbs free energies G [H] and relative energies $E_{rel} = G(^2[Cr]^{3+}) - G(^4[Cr]^{3+})$ and $E_{rel} = G(^3[Cr]^{2+}) - G(^5[Cr]^{2+})$ [eV]. Mulliken spin density at Cr.

	$^4[Cr]^{3+}$	$^2[Cr]^{3+}$	$^3[Cr]^{2+}$	$^5[Cr]^{2+}$
**Cr-N1**	2.056	2.049	2.058	2.098
**Cr-N2**	2.056	2.048	2.056	2.100
**Cr-N3**	2.064	2.059	1.987	2.287
**Cr-N4**	2.050	2.044	2.049	2.245
**Cr-N5**	2.055	2.048	2.061	2.075
**Cr-N6**	2.055	2.048	2.062	2.112
**N1-Cr-N2**	86.85	87.03	87.18	84.52
**N1-Cr-N3**	86.71	86.67	87.37	89.35
**N1-Cr-N4**	86.57	93.26	92.61	97.16
**N1-Cr-N5**	93.08	92.92	93.02	95.23
**N1-Cr-N6**	179.81	179.80	179.14	179.9
**N2-Cr-N3**	86.57	86.55	87.38	80.34
**N2-Cr-N4**	93.40	93.44	92.87	95.65
**N2-Cr-N5**	179.65	179.64	179.31	178.26
**N2-Cr-N6**	93.22	93.04	93.11	95.54
**N3-Cr-N4**	179.93	179.93	179.75	172.03
**N3-Cr-N5**	93.08	93.10	93.29	97.94
**N3-Cr-N6**	93.12	93.15	93.45	90.75
**N4-Cr-N5**	86.95	86.92	86.46	86.09
**N4-Cr-N6**	86.94	86.92	86.56	82.75
**N5-Cr-N6**	86.85	87.01	86.69	84.71
***G* / H**	–3034.330	–3034.293	–3034.473	–3034.466
**E_{rel} / eV**	0	1.01	0	0.19
**Spin density at Cr**	3.22	1.13	2.64	3.87

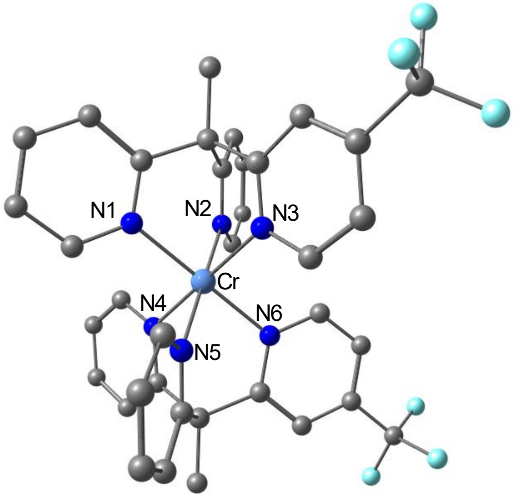

Figure S41. DFT-optimized structure of *cis*-^{4}[Cr(tpe^{CF3})$_2$]$^{3+}$ with atom labels for Table S4.

Table S4. Bond lengths [Å] and angles [deg] of *cis*-[Cr(tpe^{CF3})$_2$]$^{3+}$ in the DFT-optimized quartet and doublet states ^{4}[Cr$_2$]$^{3+}$ / ^{2}[Cr]$^{3+}$ and of *cis*-[Cr(tpe^{CF3})$_2$]$^{2+}$ in the DFT-optimized triplet and quintet states ^{3}[Cr]$^{2+}$ / ^{5}[Cr]$^{2+}$. Gibbs free energies G [H] and relative energies $E_{rel} = G(^{2}[Cr]^{3+}) - G(^{4}[Cr]^{3+})$ and $E_{rel} = G(^{3}[Cr]^{2+}) - G(^{5}[Cr]^{2+})$ [eV]. Mulliken spin density at Cr.

	^{4}[Cr]$^{3+}$	^{2}[Cr]$^{3+}$	^{3}[Cr]$^{2+}$	^{5}[Cr]$^{2+}$
**Cr-N1**	2.049	2.042	2.048	2.262
**Cr-N2**	2.056	2.048	2.070	2.079
**Cr-N3**	2.064	2.059	2.023	2.125
**Cr-N4**	2.051	2.046	2.048	2.090
**Cr-N5**	2.054	2.047	2.068	2.105
**Cr-N6**	2.063	2.056	2.022	2.312
**N1-Cr-N2**	86.96	87.15	86.75	86.12
**N1-Cr-N3**	86.81	86.79	86.78	82.07
**N1-Cr-N4**	93.34	93.37	92.80	98.94
**N1-Cr-N5**	93.33	93.17	93.11	95.88
**N1-Cr-N6**	179.85	179.83	179.46	171.73
**N2-Cr-N3**	86.67	86.63	87.08	84.26
**N2-Cr-N4**	93.17	93.22	92.98	95.51
**N2-Cr-N5**	179.65	179.62	179.78	177.96
**N2-Cr-N6**	93.07	92.88	93.06	97.71
**N3-Cr-N4**	179.77	179.78	179.57	178.95
**N3-Cr-N5**	93.15	93.18	93.09	95.67
**N3-Cr-N6**	93.04	93.05	93.71	90.99
**N4-Cr-N5**	87.01	86.97	86.85	84.52
**N4-Cr-N6**	86.81	86.79	86.71	88.02
**N5-Cr-N6**	86.63	86.80	87.08	80.25
***G* / Eh**	−3371.858	−3371.821	−3372.005	−3371.996
**E_{rel} / eV**	0	1.01	0	0.24
**Spindensity at Cr**	3.22	1.13	2.61	3.91

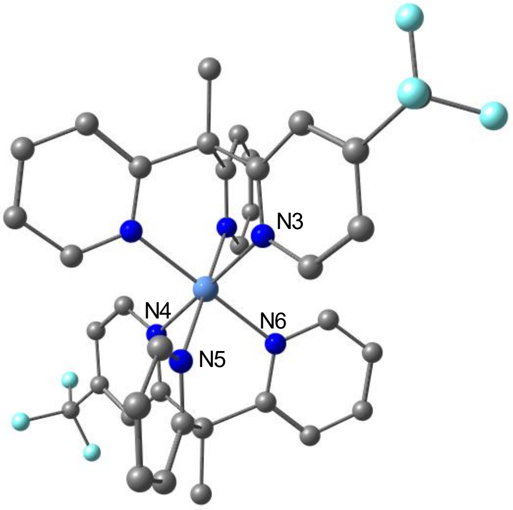

Figure S42. DFT-optimized structure of *trans*-^{4}[Cr(tpe^{CF3})$_2$]$^{3+}$ with atom labels for Table S5.

Table S5. Bond lengths [Å] and angles [deg] of *trans*-[Cr(tpe^{CF3})$_2$]$^{3+}$ in the DFT-optimized quartet and doublet states ^{4}[Cr$_2$]$^{3+}$ / ^{2}[Cr]$^{3+}$ and of *trans*-[Cr(tpe^{CF3})$_2$]$^{2+}$ in the DFT-optimized triplet and quintet states ^{3}[Cr]$^{2+}$ / ^{5}[Cr]$^{2+}$. Gibbs free energies *G* [H] and relative energies E_{rel} = *G*(^{2}[Cr]$^{3+}$) – *G*(^{4}[Cr]$^{3+}$) and E_{rel} = *G*(^{3}[Cr]$^{2+}$) – *G*(^{5}[Cr]$^{2+}$) [eV]. Mulliken spin density at Cr.

	^{4}[Cr]$^{3+}$	^{2}[Cr]$^{3+}$	^{3}[Cr]$^{2+}$	^{5}[Cr]$^{2+}$
**Cr-N1**	2.055	2.048	2.060	2.080
**Cr-N2**	2.055	2.047	2.061	2.114
**Cr-N3**	2.059	2.054	2.018	2.277
**Cr-N4**	2.060	2.055	2.018	2.287
**Cr-N5**	2.055	2.048	2.061	2.086
**Cr-N6**	2.055	2.048	2.059	2.108
**N1-Cr-N2**	86.83	87.00	86.83	84.52
**N1-Cr-N3**	86.86	86.84	86.90	87.59
**N1-Cr-N4**	93.32	93.33	93.07	98.10
**N1-Cr-N5**	93.06	92.89	93.11	95.36
**N1-Cr-N6**	179.92	179.95	179.94	178.58
**N2-Cr-N3**	86.73	86.69	86.87	81.18
**N2-Cr-N4**	93.48	93.52	93.28	92.20
**N2-Cr-N5**	179.79	179.72	179.84	179.40
**N2-Cr-N6**	93.19	93.02	93.22	95.58
**N3-Cr-N4**	179.93	179.73	179.84	170.86
**N3-Cr-N5**	93.02	93.05	92.98	98.22
**N3-Cr-N6**	93.07	93.11	93.13	93.83
**N4-Cr-N5**	86.77	86.74	86.86	88.40
**N4-Cr-N6**	86.75	86.72	86.91	80.49
**N5-Cr-N6**	86.92	87.09	86.83	84.55
***G* / Eh**	–3371.860	–3371.8203	–3372.005	–3371.998
**E_{rel} / eV**	0	1.07	0	0.19
**Spindensity at Cr**	3.22	1.13	2.62	3.90

Table S6. Energies and depictions of the orbitals used in the active space of the CASSCF(7,12)-SC-NEVPT2 calculation of $[Cr(tpe)_2]^{3+}$ (contour value of 0.03 a.u.; hydrogen atoms omitted for clarity).

#	*E* / H	orbital	#	*E* / H	orbital
146	–0.5944	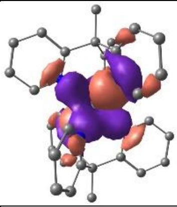	152	0.0859	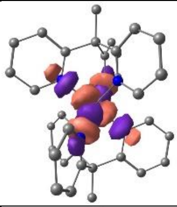
147	–0.5944	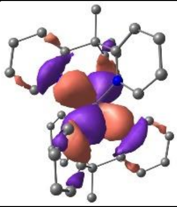	153	0.9639	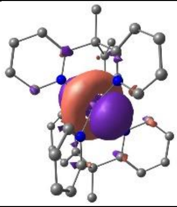
148	–0.1346	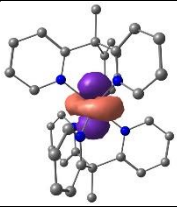	154	0.9551	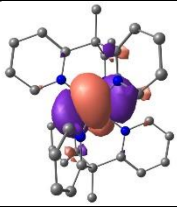
149	–0.1181	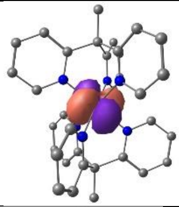	155	1.0248	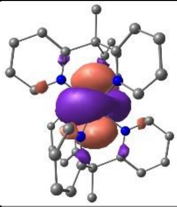
150	–0.1159	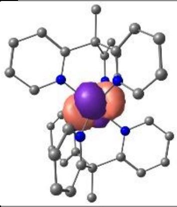	156	1.6681	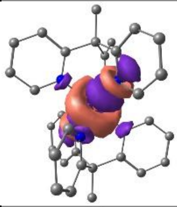
151	0.0832	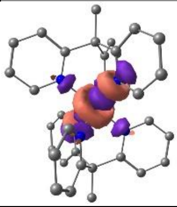	157	1.6689	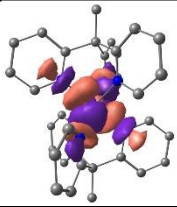

Table S7. Energies and depictions of the orbitals used in the active space of the CASSCF(7,12)-SC-NEVPT2 calculation of [Cr(tpe)(tpe^{CF3})]$^{3+}$ (contour value of 0.03 a.u.; hydrogen atoms omitted for clarity).

#	*E* / H	orbital	#	*E* / H	orbital
162	–0.5992	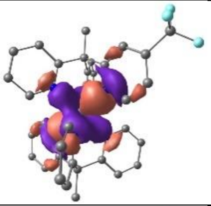	168	0.0852	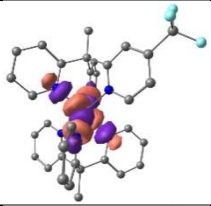
163	–0.5964	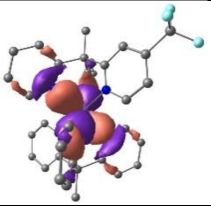	169	0.9589	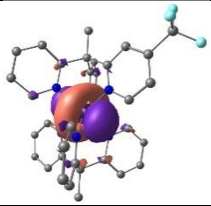
164	–0.1381	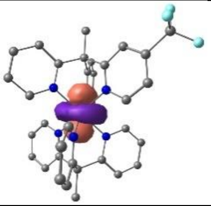	170	0.9447	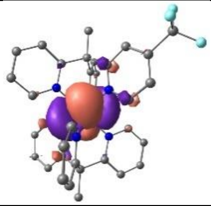
165	–0.1240	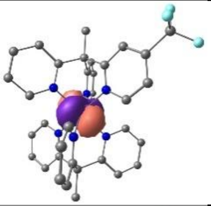	171	1.0271	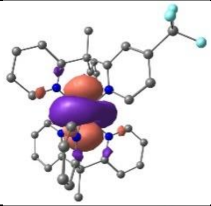
166	–0.1172	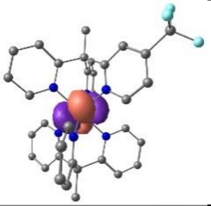	172	1.6628	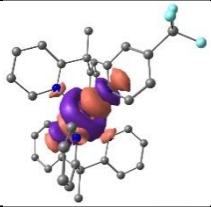
167	0.0767	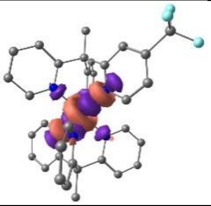	173	1.6679	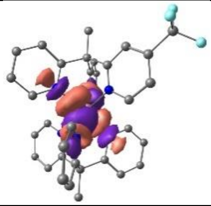

Table S8. Energies and depictions of the orbitals used in the active space of the CASSCF(7,12)-SC-NEVPT2 calculation of *cis*-$[Cr(tpe^{CF3})_2]^{3+}$ (contour value of 0.03 a.u.; hydrogen atoms omitted for clarity).

#	*E* / H	orbital	#	*E* / H	orbital
178	–0.6025	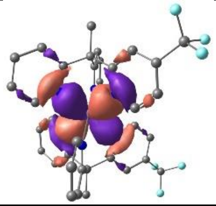	184	0.0796	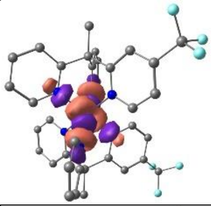
179	–0.6000	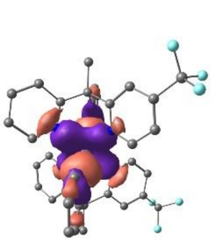	185	0.9482	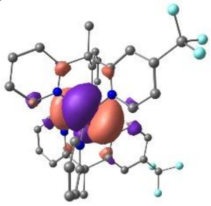
180	–0.1420	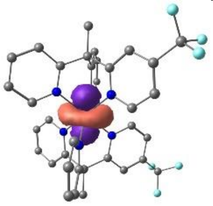	186	0.9497	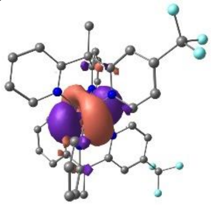
181	–0.1259	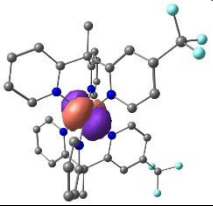	187	1.0205	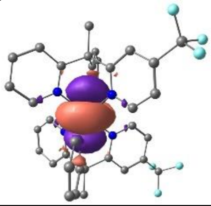
182	–0.1225	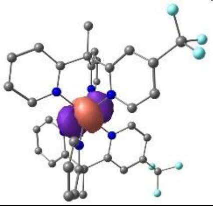	188	1.6603	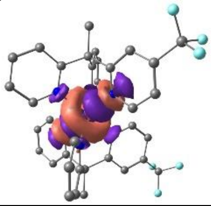
183	0.0748	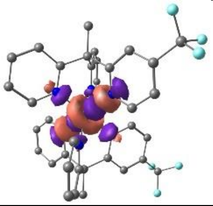	189	1.6636	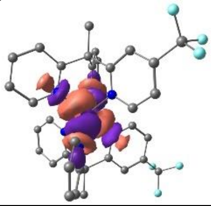

Table S9. Energies and depictions of the orbitals used in the active space of the CASSCF(7,12)-SC-NEVPT2 calculation of *trans*-[Cr(tpe^{CF3})$_2$]$^{3+}$ (contour value of 0.03 a.u.; hydrogen atoms omitted for clarity).

#	*E* / H	orbital	#	*E* / H	orbital
178	–0.6042	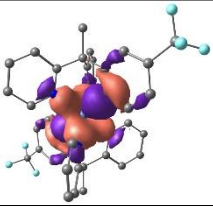	184	0.0839	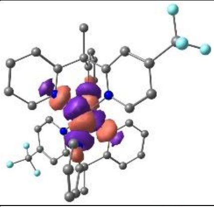
179	–0.5986	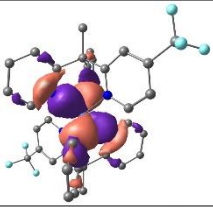	185	0.9555	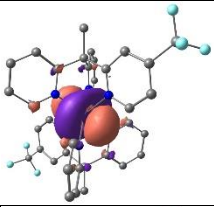
180	–0.1419	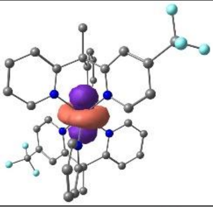	186	0.9360	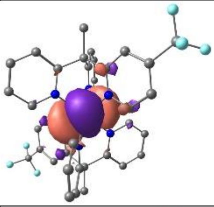
181	–0.1294	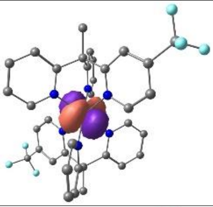	187	1.0269	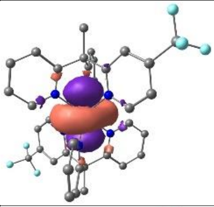
182	–0.1193	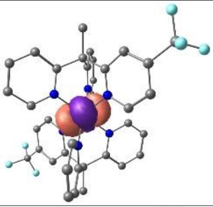	188	1.6566	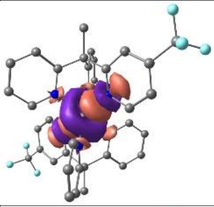
183	0.0703	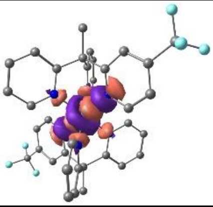	189	1.6671	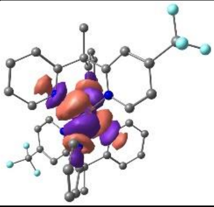

Table S10. CASSCF(7,12)-SC-NEVPT2 results of $[Cr(tpe)_2]^{3+}$ (orange) and $[Cr(tpe)(tpe^{CF3})]^{3+}$ (red); energies in cm^{-1} relative to the $^4A_2$ ground state; energies of doublet states scaled by 0.89[44,49] (bold values indicate the lowest state of each multiplicity). Light gray: doublet character, dark gray: quartet character.

Term Symbol	*E* / cm^{-1}	*E* / cm^{-1} (scaled with 0.89)	Term Symbol	*E* / cm^{-1}	*E* / cm^{-1} (scaled with 0.89)
**$^2T_1(1)$**	**15225**	**13550**	**$^2T_1(1)$**	**15219**	**13545**
$^2E(1)$	15231	13556	$^2E(1)$	15230	13555
$^2T_1(2)$	15640	13920	$^2T_1(2)$	15618	13900
$^2T_1(3)$	15644	13923	$^2T_1(3)$	15660	13937
$^2E(2)$	16571	14748	$^2E(2)$	16578	14754
$^2T_2(1)$	22729	20229	$^2T_2(1)$	22735	20234
**$^4T_2(1)$**	**24250**		**$^4T_2(1)$**	**24242**	
$^2T_2(2)$	24851	22117	$^2T_2(2)$	24805	22076
$^2T_2(3)$	24858	22124	$^2T_2(3)$	24906	22166
$^4T_2(2)$	24999		$^4T_2(2)$	24947	
$^4T_2(3)$	25001		$^4T_2(3)$	25030	
$^4T_1(1)$	30104		$^4T_1(1)$	30106	
$^4T_1(2)$	32530		$^4T_1(2)$	32413	
$^4T_1(3)$	32554		$^4T_1(3)$	32690	
$^2A_1$	37253	33155	$^2A_1$	37261	33162
$^2A_2$	38105	33913	$^2A_2$	38064	33877
$^4T_1(1)$	52119		$^4T_1(1)$	52130	
$^4T_1(2)$	52466		$^4T_1(2)$	52393	
$^4T_1(3)$	52473		$^4T_1(3)$	52520	

Table S11. CASSCF(7,12)-SC-NEVPT2 results of *cis*-$[Cr(tpe^{CF3})_2]^{3+}$ (purple) and *trans*-$[Cr(tpe^{CF3})_2]^{3+}$ (blue); energies in cm^{-1} relative to the $^4A_2$ ground state; of doublet states scaled by 0.89[44,49] (bold values indicate the lowest state of each multiplicity). Light gray: doublet character, dark gray: quartet character.

Term Symbol	*E* / cm^{-1}	*E* / cm^{-1} (scaled with 0.89)	Term Symbol	*E* / cm^{-1}	*E* / cm^{-1} (scaled with 0.89)
**$^2T_1(1)$**	**15235**	**13559**	**$^2T_1(1)$**	**15228**	**13553**
$^2E(1)$	15247	13570	$^2T_1(2)$	15239	13563
$^2T_1(2)$	15620	13902	$^2E(1)$	15597	13881
$^2T_1(3)$	15671	13947	$^2T_1(3)$	15678	13953
$^2E(2)$	16582	14758	$^2E(2)$	16581	14757
$^2T_2(1)$	22737	20236	$^2T_2(1)$	22747	20245
**$^4T_2(1)$**	**24239**		**$^4T_2(1)$**	**24200**	
$^2T_2(2)$	24798	22070	$^2T_2(2)$	24740	22019
$^2T_2(3)$	24898	22160	$^4T_2(2)$	24844	
$^4T_2(2)$	24956		$^2T_2(3)$	24948	22204
$^4T_2(3)$	25022		$^4T_2(3)$	25077	
$^4T_1(1)$	30105		$^4T_1(1)$	30059	
$^4T_1(2)$	32387		$^4T_1(2)$	32269	
$^4T_1(3)$	32700		$^4T_1(3)$	32836	
$^2A_1$	37259	33161	$^2A_1$	25077	33144
$^2A_2$	38076	33888	$^2A_2$	37960	33784
$^4T_1(1)$	52121		$^4T_1(1)$	52132	
$^4T_1(2)$	52343		$^4T_1(2)$	52267	
$^4T_1(3)$	52497		$^4T_1(3)$	52533	

References

[71] STOE & Cie, X-Area, STOE & Cie GmbH: Darmstadt, Germany.

[72] R. H. Blessing, *Acta Crystallogr. Sect. A* **1995**, *51*, 33–38.

[73] A. L. Spek, *Acta Crystallogr. Sect. D* **2009**, *65*, 148–155.

[74] G. M. Sheldrick, *Acta Crystallogr. Sect. A* **2015**, *71*, 3–8.

[75] G. M. Sheldrick, *Acta Crystallogr. Sect. C* **2015**, *71*, 3–8.

[76] G. M. Sheldrick, *Acta Crystallogr. Sect. A* **2008**, *64*, 112–122.

[77] C. B. Hübschle, G. M. Sheldrick, B. Dittrich, *J. Appl. Crystallogr.* **2011**, *44*, 1281–1284.

[78] P. J. Larkin, Infrared and Raman Spectroscopy, Elsevier Inc., 2018, 2nd ed..

[79] G. R. Fulmer, A. J. M. Miller, N. H. Sherden, H. E. Gottlieb, A. Nudelman, B. M. Stoltz, J. E. Bercaw, K. I. Goldberg, *Organometallics* **2010**, *29*, 2176–2179.

[80] F. Neese, F. Wennmohs, U. Becker, C. Riplinger, *J. Chem. Phys.* **2020**, *152*, 224108.

[81] F. Neese, *Wiley Interdiscip. Rev. Comput. Mol. Sci.* **2022**, *12*, e1606.

[82] A. D. Becke, *J. Chem. Phys.* **1993**, *98*, 5648–5652.

[83] B. Miehlich, A. Savin, H. Stoll, H. Preuss, *Chem. Phys. Lett.* **1989**, *157*, 200–206.

[84] F. Neese, F. Wennmohs, A. Hansen, U. Becker, *Chem. Phys.* **2009**, *356*, 98–109.

[85] R. Izsák, F. Neese, *J. Chem. Phys.* **2011**, *135*, 144105.

[86] D. A. Pantazis, X.-Y. Chen, C. R. Landis, F. Neese, *J. Chem. Theory Comput.* **2008**, *4*, 908–919.

[87] S. Miertuš, E. Scrocco, J. Tomasi, *Chem. Phys.* **1981**, *55*, 117–129.

[88] V. Barone, M. Cossi, *J. Phys. Chem.* **1998**, *102*, 1995–2001.

[89] A. Schäfer, H. Horn, R. Ahlrichs, *J. Chem. Phys.* **1992**, *97*, 2571–2577.

[90] A. Schäfer, C. Huber, R. Ahlrichs, *J. Chem. Phys.* **1994**, *100*, 5829–5835.

[91] S. Grimme, J. Antony, S. Ehrlich, H. Krieg, *J. Chem. Phys.* **2010**, *132*, 154104.

[92] S. Grimme, S. Ehrlich, L. Goerigk, *J. Comput. Chem.* **2011**, *32*, 1456–1465.

[93] B. O. Roos, P. R. Taylor, P. E. Sigbahn, *Chem. Phys.* **1980**, *48*, 157–173.

[94] P. E. M. Siegbahn, J. Almlöf, A. Heiberg, B. O. Roos, *J. Chem. Phys.* **1981**, *74*, 2384–2396.

[95] F. Weigend, M. Kattannek, R. Ahlrichs, *J. Chem. Phys.* **2009**, *130*, 164106.

[96] S. Kossmann, F. Neese, *Chem. Phys. Lett.* **2009**, *481*, 240–243.

[97] C. Angeli, R. Cimiraglia, S. Evangelisti, T. Leininger, J.-P. Malrieu, *J. Chem. Phys.* **2001**, *114*, 10252–10264.

[98] C. Angeli, R. Cimiraglia, J.-P. Malrieu, *J. Chem. Phys.* **2002**, *117*, 9138–9153.

[99] K. Pierloot, *Int. J. Quantum Chem.* **2011**, *111*, 3291–3301.
